# Derivatization of Microcystins Can Increase Target Inhibition while Reducing Cellular Uptake

**DOI:** 10.1021/acs.jnatprod.4c00688

**Published:** 2024-10-20

**Authors:** Laura L. Sallandt, Clemens A. Wolf, Sabine Schuster, Heike Enke, Dan Enke, Gerhard Wolber, Timo H. J. Niedermeyer

**Affiliations:** ⊥ Department of Pharmaceutical Biology, Institute of Pharmacy, Freie Universität Berlin, 14195 Berlin, Germany; § Department of Pharmaceutical Chemistry (Molecular Drug Design), Institute of Pharmacy, Freie Universität Berlin, 14195 Berlin, Germany; ‡ Simris Biologics GmbH, 12489 Berlin, Germany

## Abstract

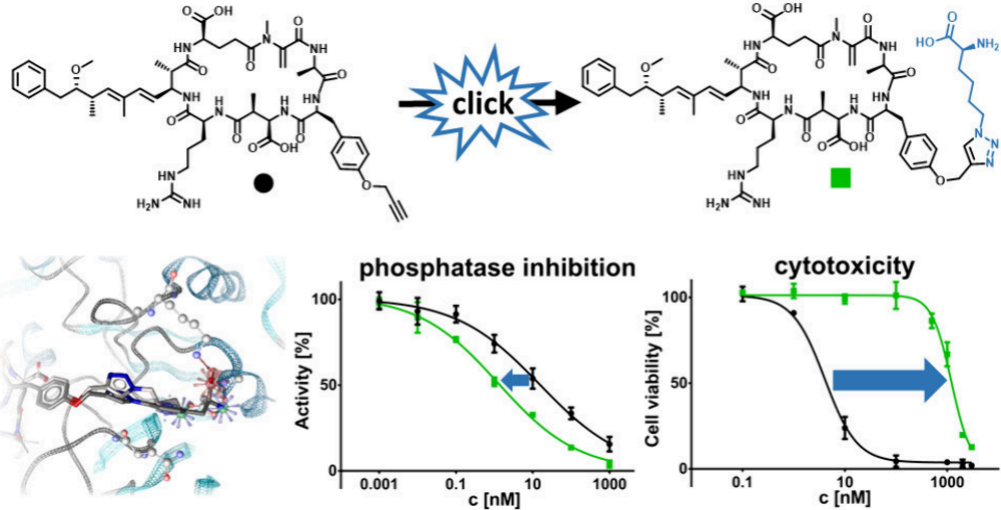

Microcystins, a large family of nonribosomal cyclic heptapeptides known for their hepatotoxicity, are among the best-studied cyanobacterial toxins. Recently, they have been discussed as leads for the development of anticancer drug substances. Their main mode-of-action is inhibition of the eukaryotic serine/threonine protein phosphatases 1 and 2A. Unlike many cytotoxins that can cross cell membranes by passive diffusion, microcystins depend on active uptake via organic anion transporting polypeptides 1B1 or 1B3. Both phosphatase inhibition and transportability strongly depend on the structure of the individual microcystin. Here, we present how chemical modification of positions 2 and 4 of the microcystin core structure can alter these two properties. Aiming to reduce transportability and increase phosphatase inhibition, we used pharmacophore modeling to investigate the phosphatase inhibition potential of microcystins derivatized with small molecules containing a variety of functional groups. The respective derivatives were synthesized using click chemistry. We discovered that some derivatized microcystins can address a yet undescribed subpocket of the protein phosphatase 1. The derivatized microcystins were tested for phosphatase 1 inhibition and cytotoxicity on transporter-expressing cell lines, revealing that target inhibition and transportability of microcystins can independently be influenced by the physicochemical properties, especially of the residue located in position 2 of the microcystin. Derivatization with small acids or amino acids resulted in microcystins with a favorable ratio of inhibition to transportability, making these derivatives potentially suitable for drug development.

Microcystins (MCs) are specialized metabolites produced by freshwater cyanobacteria such as *Microcystis*, *Nostoc*, and *Planktothrix*.^1−3^ These cyclic nonribosomal heptapeptides contain the characteristic amino acid Adda ((2*S*,3*S*,8*S*,9*S*)-3-amino-9-methoxy-2,6,8-trimethyl-10-phenyldeca-4*E*,6*E*-dienoic acid) or derivatives of Adda in position 5 of the MC core structure (see structure of MC-LR, **1**). Positions 2 and 4 have a higher amino acid diversity than the other positions, resulting in a large structural diversity of MCs.^1^ MC-LR (**1**), featuring leucine in position 2 and arginine in position 4 of the MC core structure, is the most widely found natural MC derivative. Today, more than 300 naturally occurring MCs have been described.^1,4^ They are well studied and known for their environmental toxicity.^5−8^ The cytotoxic mode-of-action of MCs is mainly based on the inhibition of the ubiquitously expressed serine/threonine protein phosphatases (PP) 1 and 2A,^9^ and structures of PP1 and PP2A in complex with a MC have been determined by X-ray crystallography.^10−17^ The high affinity of MCs for PP1 and 2A can be explained by the fit of the Adda side chain into the hydrophobic pocket of the catalytic subunit of the PP, which has also been supported by molecular modeling approaches.^12,18^ The free carboxylic acid groups of d-glutamic acid in position 6 as well as of *N*-methyl-d-aspartic acid in position 3 also contribute to PP1 and 2A binding. Based on these findings, researchers have designed PP1 and 2A inhibitors that may be of interest for drug development.^13,19−21^
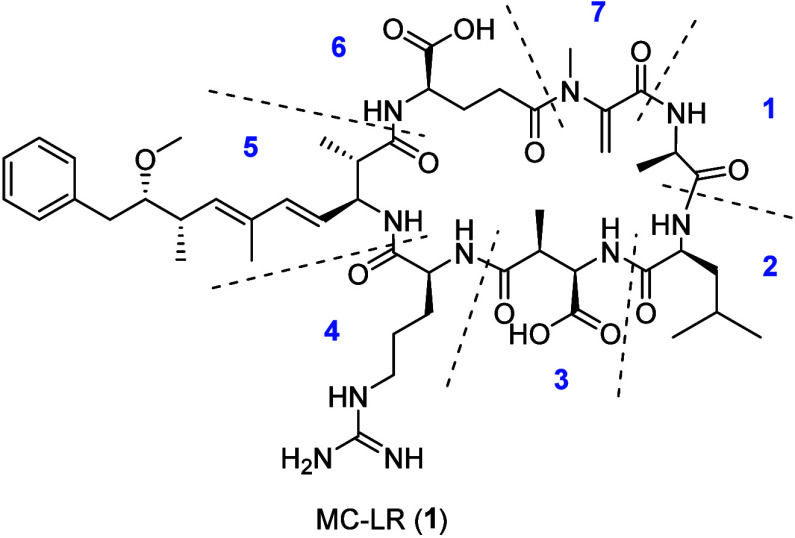


Although PPs are essential for all living cells, MCs show selective toxicity against liver cells in vivo: MCs depend on active transport across cell membranes by organic anion transporting polypeptides (OATPs), mainly OATP1B1 and OATP1B3,^22^ which are expressed by liver cells. We previously showed that the amino acids located in positions 2 and 4 of the MC core structure have a profound effect on the transportability of MCs.^23^ MCs have a net negative charge under physiological conditions. However, although the name “OATP” suggests that organic anions are preferred substrates, neutral and cationic compounds are also known to be transported.^24^ OATP1B1, encoded by the gene *SLCO1B1*, is responsible for the uptake of a wide range of substrates such as different statins, methotrexate, and bilirubin. OATP1B3, encoded by the gene *SLCO1B3*, shares overlapping substrate specificity with OATP1B1, but is also capable of transporting substrates that are unique to OATP1B3, e.g., amanitin.^25^ Attempts have been made to categorize substrates and inhibitors of OATP1B1 and OATP1B3 using modeling approaches.^26−28^ Recently, Shan et al. determined the cryo-EM structure of OATP1B1, and Ciutǎ et al. studied the cryo-EM structures of both OATP1B1 and OATP1B3, revealing similarities but also differences between these two transporters, e.g., the binding pocket of OATP1B1 is characterized by a polyspecific nature, whereas the binding pocket of OATP1B3 is narrower and contains a bicarbonate ion bound to a conserved signature motif.^29,30^ MCs also inhibit other phosphoprotein phosphatases,^31,32^ induce oxidative stress and DNA damage,^33−35^ and chemokine production,^36,37^ leading to a deadly cascade for the cell involving cytoskeletal modification and disruption,^38^ and induction of apoptosis.^39^ Exposure to MCs, e.g., through contaminated water or food,^3^ can result in chronic liver damage or acute liver failure,^40^ or to liver cancer due to DNA damage.^35^ Due to their cytotoxic properties, MCs have been discussed as leads for the development of drug substances against cancer.^23,41^ However, this has not yet resulted in the development of suitable derivatives. To lay the foundation for further exploitation of MCs as leads in drug substance development, we have recently developed a strategy for the production of clickable MCs using precursor-directed biosynthesis.^42^ Click chemistry is a useful tool for simple, fast, and selective reactions with high yields and almost no side products, and the described clickable MCs can be derivatized in bioorthogonal reactions such as copper(I)-catalyzed azide–alkyne cycloadditions (CuAAC) or strain-promoted azide–alkyne click reactions. To date, knowledge on the impact of derivatization at positions 2 and 4 of the MC core structure on the biological activity of MCs is scarce. Precursor-directed biosynthesis and subsequent derivatization by click chemistry can be used to close this knowledge gap.

Thus, here we studied the influence of a derivatization at positions 2 and 4 of the MC core structure on both target inhibition and transportability. Our objective was to identify MC derivatives that strongly inhibit PP1 while showing low uptake by OATP1B1/1B3. Such derivatives might be of interest in drug development, particularly as payloads in antibody-drug conjugates (ADCs). Molecular modeling of the PP1 interaction of MCs with azide or alkyne functional groups and of MCs derivatized with small molecules at position 2 or 4 revealed an additional potentially accessible binding site on the PP1 surface. Hypothesizing that addressing this additional binding site might result in stronger PP1 inhibition, we synthesized a series of structurally modified MCs, which we subsequently assessed in both a protein phosphatase 1 inhibition (PP1 inhibition) assay and a cytotoxicity assay using OATP1B1- or OATP1B3-expressing cell lines. We found that derivatization of MCs indeed had an influence on these two properties. Introduction of amino acids and small acids at position 2 resulted in a strongly reduced uptake via OATP1B1/1B3, while PP1 inhibition was only moderately increased.

## Results and Discussion

### Pharmacophore Modeling of MCs

It is well-known that positions 3, 5, and 6 of the MC core structure are rather conserved and that especially positions 5 and 6 are essential for PP1 and PP2A inhibition, but that positions 2 and 4 are highly variable.^10,12,13,43,44^ Thus, we hypothesized that the amino acids at positions 2 and 4 are less relevant for the interaction with the PP1, and might therefore be suitable for targeted derivatization of MCs. Based on our previous studies on the production of clickable MCs using precursor-directed biosynthesis,^42^ we selected four clickable MC-LR derivatives for this study. The selected MCs differ from **1** in only one position each, leucine being exchanged by l-propargyltyrosine (MC-PrtyrR, **2**), l-azidonorvaline (MC-AznvaR, **3**), or l-azidonorleucine (MC-AznleR, **4**), or arginine being exchanged by l-azidonorleucine (MC-LAznle, **5**). The terminal azide or alkyne functional groups were then used for derivatization by CuAAC reactions.
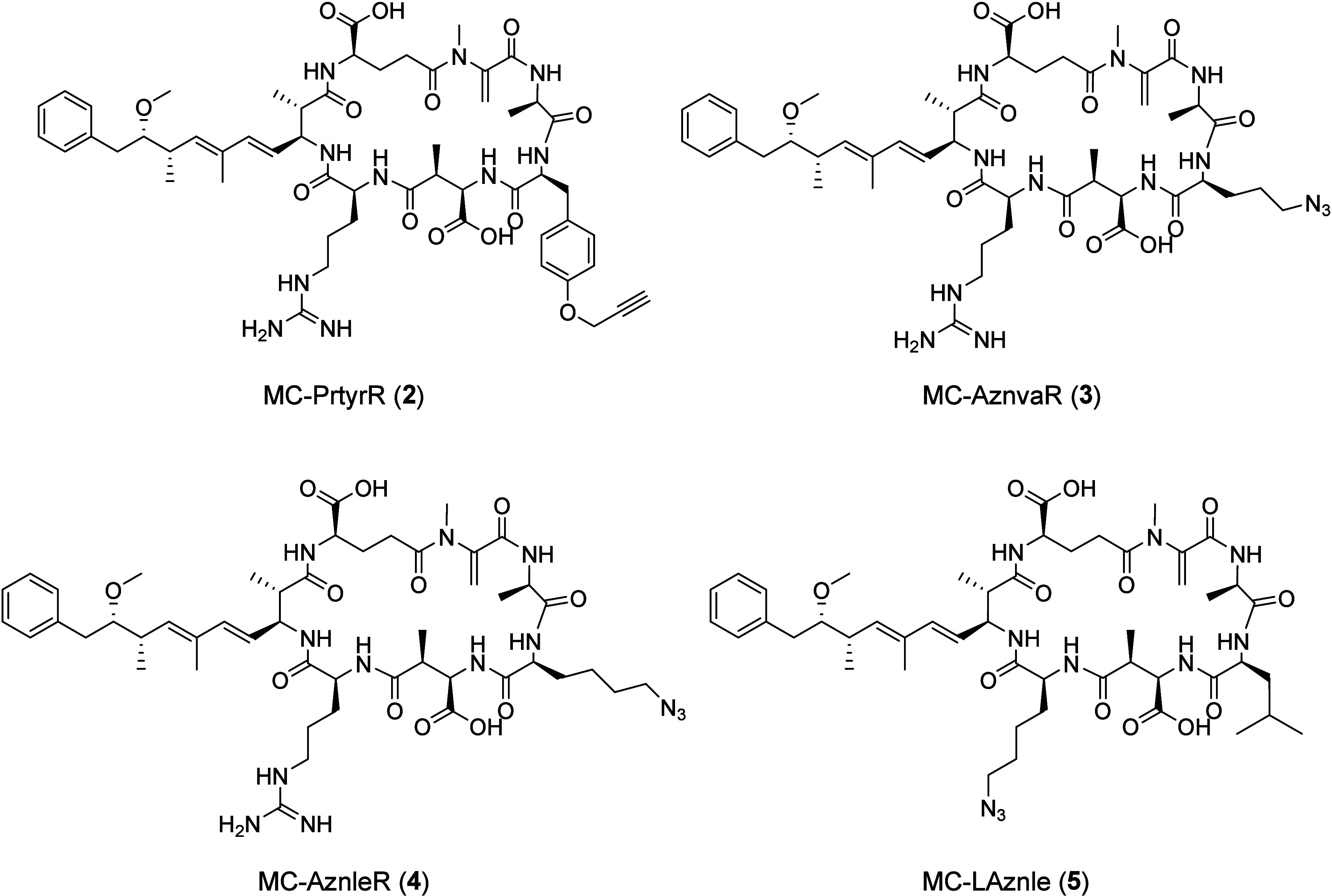


**Figure 1:**
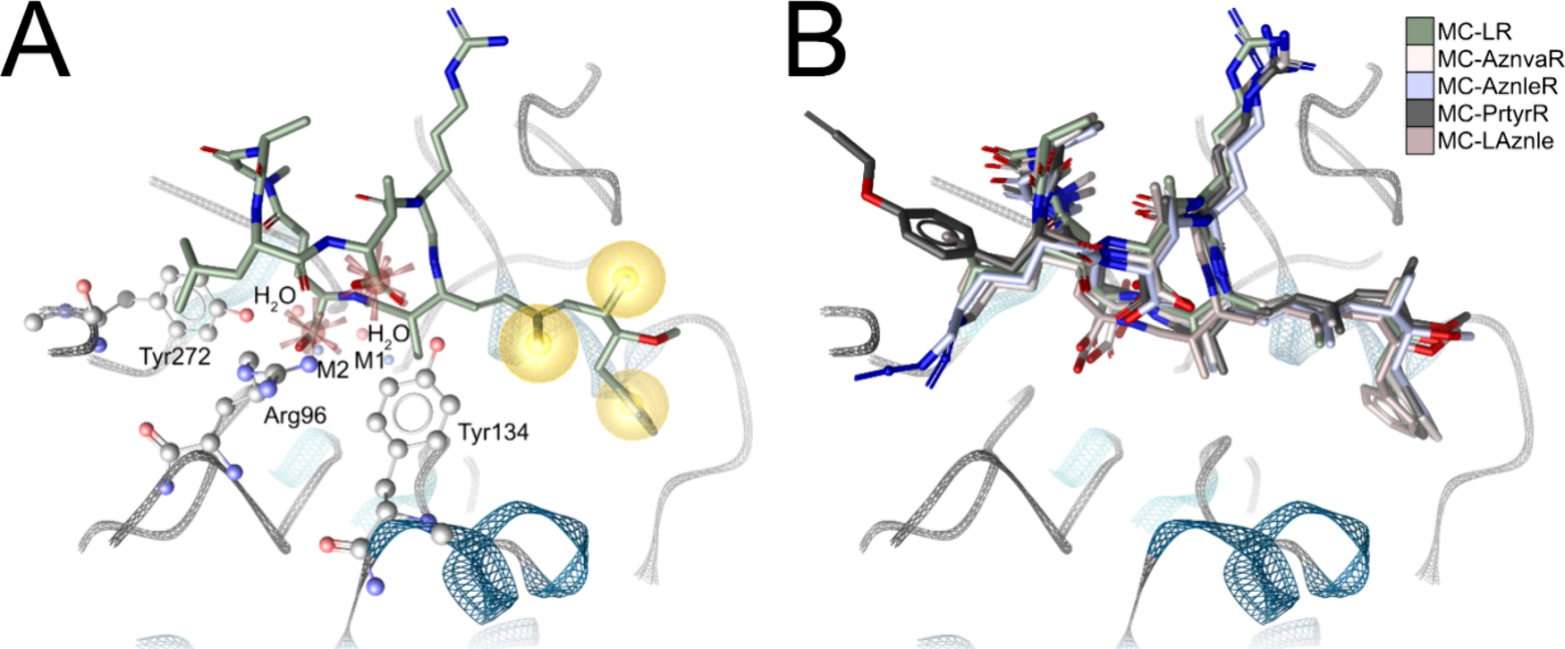
A. **1** in its PP1 binding site (PDB code 2BDX). M1, M2: Mn^2+^ ions. H_2_O: stable water molecules in the binding site. Red stars: carboxylates acting as anions and hydrogen bond acceptors; yellow spheres: hydrophobic contacts. Adda^5^ is embedded in a lipophilic subpocket; d-Glu^6^ and d-Masp^3^ point toward positively charged Arg96; d-Glu^6^ also forms ionic interactions with one of the Mn^2+^; l-Leu^2^, l-Arg^4^, and d-Ala^1^ are solvent-exposed. B. The binding conformations of **1** to **5** obtained from the MD simulations show good alignment in the pocket.

To get an initial insight into how the clickable functional groups introduced by precursor-directed biosynthesis might influence the binding behavior of the derivatives to the PP1 binding site, we explored the structural binding properties of **1** to PP1 in silico. In order to understand MC interaction patterns, we modeled **1** into the crystal structures of PP1 with cocrystallized dihydromicrocystin-LA (PDB entries 2BDX and 6OBQ).^17,45^ Compound **1** was modeled based on the cocrystallized dihydromicrocystin-LA by replacing Ala^4^ by Arg^4^ following dehydration (Figure 1A). Known interaction patterns for MCs, in particular hydrogen bonds and ionic interactions from the carboxylates d-Glu^6^ and d-Masp^3^ as well as lipophilic contacts formed by Adda^5^ could be reproduced in our model.^13,18,21,43,46−49^ A detailed description of shared interactions in the observed binding modes in our model is provided in Table S1.

Our model confirmed our hypothesis that the side chains of l-Leu^2^ and l-Arg^4^ are indeed not involved in the interaction with the PP1: They, and in addition d-Ala^1^ are arranged in a solvent-exposed manner (Figure 1B). Thus, derivatization of MCs in these positions should not negatively affect PP1 inhibition.

In order to identify subtle differences in binding modes, we investigated the molecular dynamics of these noncovalent interactions. We simulated the PP1/MC complexes of **1** (3 × 100 ns) before and after Michael addition of Mdha^7^ to Cys273 of the enzyme, i.e., in covalent and noncovalent form. Notably, the formation of this covalent bond is not important for PP1 inhibition by MCs, as shown by Holmes and co-workers.^11,17^ In line with this, no substantial changes in the frequencies of the interactions of d-Masp^3^, d-Glu^6^, or Adda^5^ with the enzyme were found after formation of the covalent bond. The dynamic interaction pattern (dynophore model)^50−54^ is shown in Figure S1A for the noncovalent complex, and in Figure S1B for the covalent form. We observed that the ionic interactions and hydrogen bonds by the carboxylates of d-Masp^3^ and d-Glu^6^ and the lipophilic contacts between Adda^5^ and the lipophilic groove of PP1 are stable throughout the simulations (Table S2).

We used the model for **1** in PP1 to fit the clickable MCs **2** to **5** into the PP1 binding pocket. Direct comparison in silico revealed highly similar interaction profiles between PP1 and each MC. All four PP1–MC complexes were subjected to MD simulations of 100 ns in three replicas, as in the previous case of **1** to ensure comparability. We found that **2** to **5** show the same interactions as **1** with comparable frequency (Figure S1C–F, Table S2). All compounds showed a highly similar, stable binding conformation in the pocket (Figure 1B). The terminal alkyne or azide groups of **2** to **5** do not engage in additional interactions with PP1. This agrees well with our experimental data showing that MCs **1** to **5** inhibit PP1 to a comparable extent, with the IC_50_ ranging from 5.5 nM (**1**) to 11.3 nM (**4**) (Table 2). We found a slightly stronger PP1 inhibition by **1** compared to **5**, which might be due to the slightly more hydrophilic Arg^4^ in **1** compared to Aznle^4^ in **5**.^55^

### Pharmacophore Modeling Revealed an Additional PP1 Binding Site Reachable for MC Derivatives

**Figure 2:**
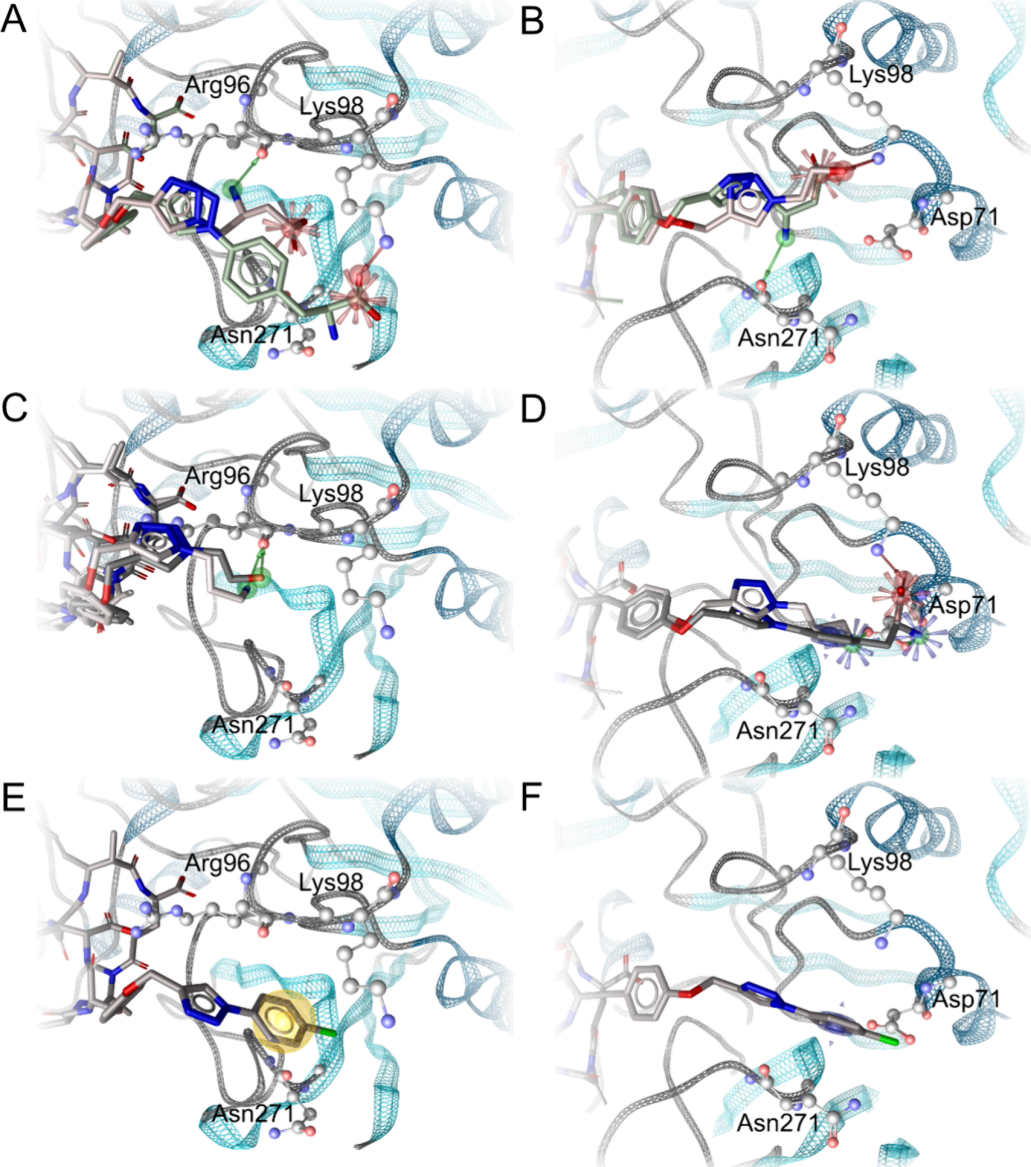
A, C: Suggested binding modes of **2i** (light gray), **2f** (dark gray), **2g** (green-gray), and **2s** (red-gray) in protein conformation 2BDX. B, D: Suggested binding modes of **2i**, **2f**, **2g**, and **2s** in protein conformation 6OBQ. E, F: Suggested binding modes of **2u** in protein conformations 2BDX and 6OBQ. Green arrows: hydrogen bonds with the ligand acting as donor. Red arrows: hydrogen bonds with the ligand acting as acceptor. Red stars: ionic interactions with anionic ligand moiety. Blue stars: ionic interactions with cationic ligand moiety. Blue donut: cation−π interactions. Yellow spheres: hydrophobic contacts.

Further exploration of the protein surface around the main MC binding site revealed an extended subpocket adjacent to position 2 of the MC core structure (Figure S1G, H). This canyon-like superficial pocket is potentially well-suited to accommodate residues attached to the clickable functional groups in **2** to **4**. The molecular environment near position 4 of the MC core appears shallower, suggesting that derivatives of **5** might form fewer additional beneficial interactions with the PP1. Due to the limited depth of the latter binding pocket and its suboptimal characteristics for strong drug interactions, this shallow pocket can be considered as less druggable.^56^ We wondered whether addressing these additional subpockets might enhance the interaction between MC and PP1. Thus, 26 clickable small molecules were obtained, containing different functional groups including carboxylic acids and basic amines, under physiological conditions uncharged hydroxy, amide, biotin, halogen containing groups, and a variety of amino acids (Table 1), and the 34 MCs **2a** to **5z** listed in Table 2 were synthesized and tested for PP1 inhibition and transportability via OATP1B1 and OATP1B3. In parallel, all MC derivatives were docked into the PP1 binding pockets, allowing us to rationalize the experimentally obtained PP1 inhibition data. We used two distinct protein structures (PDB 2BDX and 6OBQ) to analyze this subpocket,^45^ differing in the conformation of the loop 272–277. In 6OBQ, there is a covalent bond between Cys273 and Mdha^7^, while there is no such bond in2BDX. The loop 272–277 is fixed into position after formation of this covalent bond, and the loop needs to bend slightly toward Mdha^7^ before bond formation. This indicates that the loop 272–277 is flexible. Bending of the loop toward Mdha^7^ makes the additional binding site more accessible. The MCs synthesized in this study are sterically more demanding in position 2 than natural congeners like MC-LR. This could impede binding to PP1 without rearrangement of loop 272–277. As the loop is presumably flexible, the following binding mode rationalization is based on protein conformation 6OBQ, since the more accessible binding site allows for more plausible binding conformations. A comparison of the binding pockets is provided in Figure S1: Figure 2A, C, and E show plausible binding modes in the protein conformation of PDB 2BDX. Figure 2B, D, and F show plausible binding modes in the protein conformation of PDB 6OBQ.

**Table 1:**
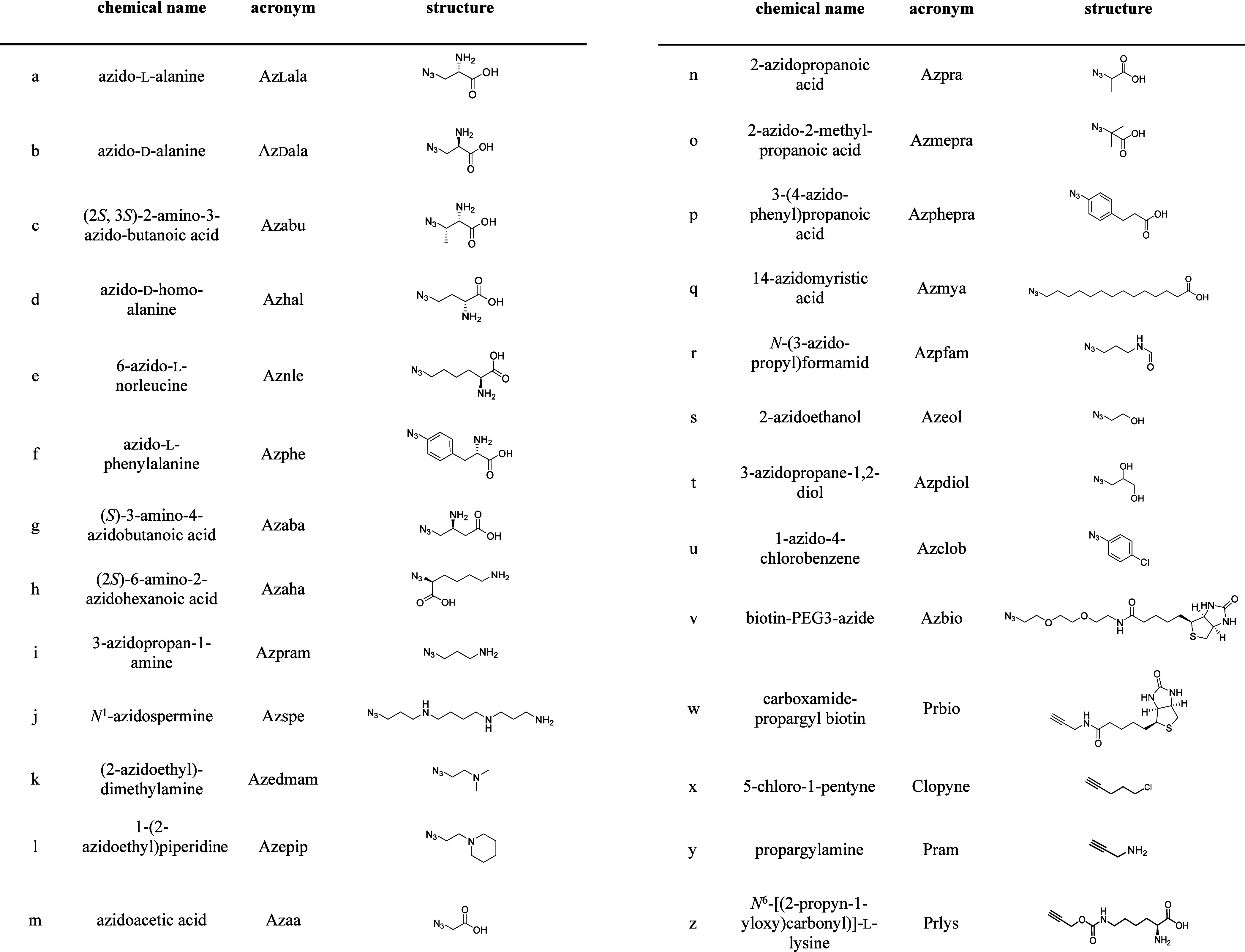
Clickable Small Molecules Used for MC Derivatization

**Table 2:** IC_50_ Values in the PP1 Inhibition Assay and EC_50_ Values in the Cytotoxicity Assay of Underivatized MCs **1** to **5** and Their Associated Derivatives; PP1 Inhibition Assay: Two Independent Quadruplicate Experiments, OATP Assay: Two Independent Triplicate Experiments

	IC_50_ PP1 Inhibition (mean ± SE; nM)	Ratio IC_50_ PP1 inhibition MC derivative/underivatized MC	EC_50_OATP1B1 (mean ± SE; nM)	Ratio EC_50_ OATP1B1MC derivative/underivatized MC	Ratio EC_50_ OATP1B1/PP1 inhibition	EC_50_ OATP1B3 (mean ± SE; nM)	Ratio EC_50_ OATP1B3MC derivative/underivatized MC	Ratio EC_50_ OATP1B3/PP1 inhibition	Selectivity index OATP1B1/OATP1B3
**1**	5.5 (±1)	–	8.8 (±9)	–	1.6	9.3 (±3)	–	1.7	1.0
**2**	8.4 (±5)	–	7.6 (±6)	–	0.9	11 (±5)	–	1.3	0.7
**2a**	8.2 (±1)	1.0	1100 (±200)	150	130	590 (±90)	54	72	1.9
**2b**	8.6 (±5)	1.0	580 (±10)	76	67	280 (±30)	25	33	2.1
**2c**	17 (±2)	2.0	150 (±70)	20	8.8	210 (±50)	19	12	0.7
**2d**	6.5 (±7)	0.8	290 (±60)	38	45	820 (±80)	75	130	0.4
**2e**	1.9 (±1)	0.2	1600 (±200)	211	840	1400 (±200)	127	740	1.1
**2f**	5.1 (±3)	0.6	250 (±8)	33	49	130 (±10)	12	24	1.9
**2g**	6.5 (±3)	0.8	2100 (±200)	276	320	460 (±20)	42	71	4.6
**2h**	1.1 (±0.2)	0.1	3300 (±200)	434	3000	1100 (±40)	100	1000	3.0
**2i**	1.3 (±0.4)	0.2	190 (±20)	25	150	110 (±50)	10	85	1.7
**2j**	2.8 (±1)	0.3	540 (±100)	71	190	1100 (±30)	100	390	0.5
**2k**	1.1 (±0.8)	0.1	150 (±40)	20	140	130 (±20)	12	120	1.2
**2l**	6.5 (±2)	0.8	4.1 (±2)	0.5	0.6	83 (±20)	7.6	13	0.05
**2m**	13 (±5)	1.5	1000 (±200)	130	77	290 (±90)	26	22	3.4
**2n**	1.2 (±2)	0.2	1500 (±200)	170	1300	130 (±40)	12	110	12
**2o**	1.1 (±0.3)	0.1	790 (±90)	100	720	130 (±20)	12	120	6.1
**2p**	4.2 (±2)	0.5	4.3 (±3)	0.6	1.0	12.4 (±2)	1.2	3.0	0.4
**2q**	140 (±40)	17	23 (±4)	3.0	0.16	36 (±6)	3.3	0.26	0.6
**2r**	1.0 (±0.4)	0.1	160 (±40)	21	160	370 (±5)	34	370	0.4
**2s**	0.45 (±0.05)	0.1	440 (±20)	58	980	370 (±20)	34	820	1.2
**2t**	1.5 (±0.6)	0.2	170 (±40)	22	110	160 (±20)	15	110	1.1
**2u**	7.0 (±3)	0.8	3.7 (±3)	0.5	0.5	11 (±4)	1.0	1.6	0.3
**2v**	1.0 (±0.2)	0.1	130 (±6)	17	130	290 (±10)	26	290	0.4
**3**	8.1 (±3)	–	24 (±10)	–	3.0	27 (±2)	–	3.3	0.8
**3w**	16 (±0.9)	2.0	130 (±6)	5.4	8.1	120 (±60)	4.4	7.5	1.1
**3x**	16 (±5)	1.9	20 (±6)	0.8	1.3	13 (±5)	0.5	0.8	1.5
**3y**	6.4 (±2)	0.8	410 (±70)	17	64	100 (±2)	3.7	16	4.1
**3z**	15 (±4)	1.8	550 (±70)	23	37	420 (±20)	16	28	1.3
**4**	11 (±3)	–	7.6 (±8)	–	0.7	8.9 (±0.4)	–	0.81	0.9
**4w**	3.1 (±3)	0.3	260 (±20)	34	84	250 (±50)	28	80	1.0
**4x**	1.1 (±0.7)	0.1	35 (±10)	4.6	32	11 (±2)	1.2	10	3.2
**4y**	3.0 (±1)	0.4	1300 (±300)	171	430	490 (±100)	55	160	2.7
**4z**	6.2 (±2)	0.8	2100 (±60)	276	340	2300 (±300)	258	370	0.9
**5**	8.3 (±0.1)	–	4.2 (±3)	–	0.5	2.4 (±2)	–	0.3	1.8
**5w**	8.9 (±1)	1.1	4.4 (±4)	1.0	0.5	4.7 (±4)	2.0	0.5	0.9
**5x**	8.5 (±2)	1.0	2.4 (±3)	0.6	0.3	5.0 (±5)	2.1	0.6	0.5
**5y**	2.8 (±2)	0.3	23 (±20)	5.5	8.2	4.1 (±0.1)	1.7	1.5	5.6
**5z**	6.2 (±2)	0.8	27 (±10)	6.4	4.4	3.6 (±2)	1.5	0.6	7.5

### PP1 Inhibition Was Only Moderately Affected by MC Derivatization at Positions 2 and 4

For a better comparison of the inhibition potential of derivatized MCs with the respective underivatized MCs **2**–**5**, the ratio of the IC_50_ value of the derivatized MC to the IC_50_ value of the underivatized MC was calculated (Table 2). In our docking experiments, all **2**-based substituents sufficiently occupy the new subpocket in well-aligned conformations (with the notable exception of **2q**), as the tube-like shape of the side pocket allows for stretched-out, flexible substituents. Residues repeatedly suggested to contribute to ligand stabilization include Lys98, Asp71, and Asn271, which form a polar interaction site (Figure 2). Binding of ligands to PP1 has been demonstrated to be facilitated by interactions with Asp71 before for the protein spinophilin.^57^

Derivatization of **2** with amino acids (**a**–**h**) had a moderate effect on the PP1 inhibition (ratios from 0.1–2.0), with derivatives **2d**–**2h** showing slightly better inhibition (ratio <0.8). All amino acid derivatives in our study are indicated to form ionic interactions with Lys98 (Figure 2B and D, Table S3) by our model, and **2a** and **2e**–**2h** additionally act as acceptors engaging in hydrogen bonds to Lys98, mainly to N_α_ or N_ζ_ (Figure 2B and D). Furthermore, **2a**–**2c** and **2g** are suggested to form hydrogen bonds to Asn271 by modeling, mainly to the backbone carbonyl moiety (Figure 2B, Table S3). **2a**, **2b**, **2d**–**2f**, and **2h** are suggested to form ionic interactions with Asp71 (Figure 2C), while **2d**–**2f** and **2h** act as hydrogen bond donors to Asp71, mainly to O_δ_ (Figure 2D, Table S3).

All MCs derivatized with small molecules containing amino groups, **2i**–**2l,** showed increased PP1 inhibition compared to the underivatized MC **2** (ratios 0.1–0.8). Our in silico model suggests that these derivatives form charged interactions with Asp71 (Figure 2D), and that in addition **2i**–**2j** act as hydrogen bond donors to Asp71, mainly to O_δ_ (Figure 2D, Table S3).

The results of the PP1 inhibition assay in the group of acidic derivatives (**2m**–**2q**) were heterogeneous. Derivatives **2m**–**2p** have similar IC_50_ as **2**. The slightly stronger inhibition of some of the derivatives might be explained by additional ionic interactions to Lys98 and also by their substituents acting as acceptors engaging in hydrogen bonds to Lys98 indicated by our model, mainly to N_α_ or N_ζ_ (Table S3). Remarkably, **2q** showed significantly lower activity, with an IC_50_ value about 17-fold higher than **2**. Compound **2q** is the only tested derivative that does not have a suitable substructure for forming hydrogen bonds or ionic interactions with any of the previously mentioned residues (Lys98, Asp71, or Asn271). This inability may hinder the Azmya residue from occupying the newly discovered lengthy subpocket or at least lead to less stabilization in the side pocket, which might result in the observed increased IC_50_.

Derivatization with uncharged functional groups (**2r**–**2v**) decreased the IC_50_ values in the PP1 inhibition assay of the derivatized MCs. This could be explained by **2r**–**2t** and **2v** acting as acceptors in hydrogen bonds with Lys98, mainly N_α_ or N_ζ_, (Figure 2B, Table S3) according to our model. The lipophilic substituent of **2u** is indicated to engage in cation−π interactions (phenyl group with Lys98, Figure 2D, Table S3). Additionally, hydrogen bond-like interactions between the terminal N_δ_ of Asn271 and chlorine, acting as weak hydrogen bond acceptor, may contribute to the stabilization of the 4-chlorobenzene substructure. There is some evidence for the relevance of polar out-of-plane interactions between uncharged NH groups in amide structures and the belt-like formation of high electron density around the C–Cl bond.^58,59^

The IC_50_ values in the PP1 inhibition assay for **3w**, **3x**, and **3z** are up to 2-fold higher compared to the underivatized MC, while the data for **3y** suggests slightly stronger inhibition (ratio 0.8). In contrast, all derivatives of **4** inhibit the PP1 in a stronger manner with ratios up to 0.1. The derivatives of **3** and **4** can occupy the same novel additional subpocket as identified for the **2** derivatives, with similar binding modes. Compounds **3w**, **4w**, **3z**, and **4z** are assumed to engage in hydrogen bonds with Lys98 or Asp71, as well. Compounds **3x** and **4x** may be stabilized by similar polar interactions involving Asn271 as discussed above (Figure 2F). Although the side-chains of **3y** and **4y** are too short to reach Lys98 or Asp71, they may occupy the same additional binding site forming polar interactions with Asn271.

In contrast to the derivatives of **2**–**4**, the derivatizable functional group of **5** is located at a different position of the MC core. This should lead to an arrangement appropriate to occupy a different subpocket on that side of the MC macrocycle. This subpocket is shorter and shallower than the pocket previously analyzed. Compounds **5w** and **5z** are expected to be stabilized by hydrogen bonds to polar residues, just like their **3w**, **4w**, **3z**, and **4z** counterparts. Compound **5y** can be stabilized by hydrogen bonds acting as donor and possibly charged interactions, much like derivatives **2i**, **3y**, and **4y**. Derivative **5x** is likely stabilized by halogen bonds or the hydrogen-bond like interactions previously discussed for **2u**, **3x**, and **4x**.

In summary, for some of the derivatives (**2h**, **2i**, **2k**, **2n**, **2o**, **2r**, **2s**, **2t**, **2v**), a slightly stronger inhibition (ratios 0.1–0.2) is observed. This is suggested to be due to interactions with Lys98, Asp71, or Asn271. More importantly, derivatization at position 2 or 4 usually does not have a negative impact on the PP1 inhibition, with most MC derivatives displaying a PP1 inhibition ratio between 0.5 and 2.0. This is consistent with previous findings,^18,60,61^ and can, especially with derivatization at position 2, be easily explained by the fact that this position is as far as possible away from the pharmacophore of MCs (Adda^5^-Glu6^6^).^43^

### OATP1B1/1B3 Transportability Was Strongly and Differentially Affected by MC Derivatization at Position 2

The cytotoxicity of the MCs was assessed in cell viability assays using HEK293 cells stably transfected with OATP1B1 and OATP1B3, and used as a surrogate parameter for transportability.^42,62^ A high cell viability in combination with an improved or unaffected PP1 inhibition indicates that the OATP transportability is reduced. Again, to facilitate comparison of the underivatized MCs **2** to **5** with their derivatives, the respective transportability ratio was calculated based on the determined EC_50_ values. A ratio below 1 indicates improved uptake, while a ratio above 1 indicates impaired uptake. The transportability of all four underivatized MCs can be considered as high, as their EC_50_ values against both cell lines were in the low nanomolar range (Table 2, Figure S2).

In general, we observed that derivatization at position 2 often reduced uptake, whereas derivatization at position 4 only had a minor impact. MCs derivatized at position 2 differed strongly in their cytotoxicity. Some derivatives retained the cytotoxicity of the underivatized MC, while others were more than 400-fold less active. It was difficult to deduce unambiguous structure-transportability relationships, but some groups of small molecules had a higher impact than others.

A selectivity index was calculated to facilitate comparison of the EC_50_ values against cells expressing OATP1B1 or OATP1B3. Interestingly, for most of the semisynthetic MCs, no significant difference could be detected between uptake into the two cell lines expressing either OATP1B1 or 1B3 (selectivity indices between 0.5 and 2.0, Table 2). While Ciutǎ et al. found distinct differences between the orthosteric binding sites of the two transporters,^30^ which could help to elucidate the variations in the uptake of some of our functionalized MCs,^25,27,29^ Shan et al. suggested that there are some similarities between the two transporters.^29^ These similarities contribute to the large overlap of transporter substrate specificity and can explain the similarities in the uptake of our MC derivatives. Niedermeyer et al. investigated 23 naturally occurring MC congeners with regard to their selectivity of OATP1B3 uptake over OATP1B1 uptake, and observed various MCs with a preferred uptake by OATP1B3.^23^ In the present study, we also observed more frequently a preferred uptake via OATP1B3 over OATP1B1 than vice versa, resulting in selectivity indices above 1.

### Derivatization with Amino Acids Resulted in the Most Pronounced Uptake Inhibition

The zwitterionic character of amino acids may contribute to the reduced uptake of these derivatives (**a**–**h**, **z**). Our results indicate that in addition to the zwitterionic nature of the amino acid residues, properties like the size of the amino acid or its side chain can contribute to uptake differences. Also, the configuration (d or l) of the attached amino acids seems to have an effect on uptake: Both **2a** and **2b** showed decreased uptake by both OATPs with selectivity indices around 2, but **2a** was approximately three times less cytotoxic than **2b**. Compound **2d**, which has only one methylene group more than **2b**, exhibited lower uptake in the cell line expressing OATP1B3. However, the OATP selectivity was reversed. We also observed an influence on OATP uptake depending on the used amino acids. The MC derivative with a β-amino acid, **2g**, displayed a lower uptake into OAPT1B1 expressing cells (selectivity index 4.6), while the related MC with an α-amino acid, **2c,** had a reversed selectivity with a lower uptake into OATP1B3 expressing cells, and also an overall higher cytotoxic effect on both cell lines. Compound **2h** (ε-amino acid) exhibited the lowest overall uptake into the OATP1B1 cell line. In the OATP1B3 cell line, the ε-amino acid derivative showed a slightly better uptake compared to the α-amino acid derivative **2e** (1100 ± 40 nM vs 1400 ± 200 nM), although both groups had a significant impact on uptake.

Introducing an amine at position 2 also influenced transportability, likely by rendering the MCs net neutral. All tested MCs with a primary amine (**2i**, **3y**, **4y**) showed reduced uptake, similar to the amino acids with differences within the group: The cytotoxicity of the tested primary amine derivatives was lower for the OATP1B1 cell line compared to the OATP1B3 cell line (selectivity indices >2.5), except for **2i**, which showed the lowest impact on both cell lines within the group of the primary amines, and had a selectivity index of 1.6. Besides, the spermine modification in **2j** also had a significant influence on uptake. An explanation might be the extra charges from the multiple amino groups, giving the molecule a strongly cationic character, making it less likely to be taken up by OATP1B1 or OATP1B3, which preferably transport anions.^29^ The tested tertiary amines did not give consistent results. **2k** had an impact on uptake by both cell lines with ratios greater than 10. Only **2l** had similar EC_50_ values to the underivatized MC, maybe due to its higher lipophilicity compared to the other amines.

MCs derivatized with small acidic compounds like **m** to **o** were among the compounds showing the worst transportability in cells expressing OATP1B1 (highest absolute EC_50_ values), along with some of the amino acid derivatives. We found a distinction between the uptake into the OATP1B1 and OATP1B3 expressing cell lines, as the acidic function only had an intermediate effect on the OATP1B3 expressing cells (e.g., **2m**, EC_50_ 290 nM). For the larger acidic compounds **p** and **q**, we observed that the EC_50_ values did not significantly differ from the underivatized MC. The high uptake of **2q** might arise from the fact that, at least for OATP1B1, Shan et al. determined a favored uptake of large organic anions, as these molecules bind more effectively in the cavity of OATP1B1.^29^ Izumi et al. described how the position and type of individual functional groups in fluorescein derivatives can affect the transport by OATP1B1 and OATP1B3, and how carboxylic acid groups can influence the uptake.^63^ Even though **2p** is structurally similar to **2f**, it was observed that the presence of the acidic function alone is not sufficient to impact the uptake. These results do not indicate that the presence of an acidic function, and thus a negative charge, results in a high uptake in general.

In addition to the charged functional groups discussed above, we also synthesized and tested derivatives with functional groups without a net charge, like amides, alcohols, biotin groups, or halogenated small molecules. The MC derivative with an amide group, **2r**, showed an impaired uptake by both cell lines, but in contrast to most of the other derivatives with a preferred uptake by OATP1B1. Hydroxy groups (**s**, **t**) also seemed to have an impact on transportability. Compound **2s**, featuring one hydroxy group, was transported less efficiently than **2t** with two hydroxy groups. Both derivatives had no major selectivity preference. Surprisingly, although it has previously been reported that attaching biotin to a MC inhibits its uptake,^64^ in our hands, the presence of biotin groups at position 2 only had a slight influence on the uptake of MCs into OATP-expressing cells. Within the group of biotin derivatives, **4w** was less well transported than **3w**, even though the difference between these two MCs is only a methylene group. Additionally, the cytotoxicity of **2v** was cell-line-dependent, with lower EC_50_ values for the OATP1B1-expressing cell line compared to the OATP1B3-expressing cell line. In contrast to the other uncharged small molecules, the only group without any effect on the uptake by the two cell lines was the halogen in the small molecules **u** and **x**. This might be due to the overall hydrophobic character of these groups contributing to a preferred uptake, at least by OATP1B1.^29^

### OATP1B1/1B3 Transportability Is Only Moderately Affected by Derivatization at Position 4

Compared with derivatization at position 2, derivatization at position 4 interestingly had much less impact on transportability. Our results indicate that amino acids and amine groups again had an influence on the transportability at least on the OATP1B1-expressing cell line, but compared to position 2, it was only a minor effect. However, it is difficult to directly compare the MCs modified at position 2 or 4: All MCs derivatized at position 2 contain Arg in position 4, whereas in **5**, the Arg is replaced by the clickable amino acid. The presence of this Arg, which is protonated under physiological conditions, might have an influence on transportability on its own. Niedermeyer et al. also observed a lower cytotoxicity of MCs containing Arg.^23^ While Leu in position 2 was suspected to contribute to a lower OATP1B3 transportability,^23^ the current study does not confirm this hypothesis.

### Decreased PP1 Inhibition Does Not Necessarily Impact Cytotoxicity of Derivatized MCs

Interestingly, MC derivatization mostly had a stronger impact on OATP-mediated uptake than on PP1 inhibition. Only one MC derivative, **2q**, showed a reduced PP1 inhibition while still being highly active in the cytotoxicity assay. This suggests that the uptake of this MC is unhindered and that a PP1 IC_50_ of 140 nM is still potent enough to cause cytotoxicity. A better OATP1B1/1B3 uptake caused by the lipophilic side chain might compensate for the lower PP1 inhibition.^65^ Moreover, besides PP1 inhibition, other cytotoxic mechanisms of the MCs can be of importance for cell death.^31,32^ It has been found before that MCs exhibiting higher IC_50_ values in PP1 inhibition assays were still highly cytotoxic in cell models.^66,67^

### Potential Use of Derivatized MCs in Drug Development

Due to their potent inhibition of protein phosphatases, MCs have been discussed as leads for anticancer drugs.^23,41^ Protein phosphatases are attractive, but not yet exploited, targets for anticancer drugs because functional protein phosphatases are essential for cell survival, and resistance development is unlikely.^20^ However, MCs cannot be used for this purpose due to their high hepatotoxicity, which is due to OATP1B1- and OATP1B3-mediated uptake of the MCs into liver cells. Our study suggests that MCs could potentially be rendered nontransportable while retaining their potent PP1 inhibition, making them attractive for the development of payloads for ADC. The identification of payloads with low general side effects after unintended release is one of the goals of current ADC development that has not yet been achieved.^68^ Given that MCs cannot enter cells by passive diffusion, with reduced uptake by OATPs after derivatization, optimized MC derivatives would solely be transported into target cells through endocytosis after antigen–antibody interaction, but not by passive diffusion after accidental release of the MC from the antibody or after the death of affected cells. MC derivatives of interest for potential payloads in ADCs are thus those with a favorable ratio of uptake to inhibition, e.g., **2h**, which shows high PP1 inhibition but strongly impaired uptake into both cell lines (Table 2). However, even the least cytotoxic derivatives presented in this study still show EC_50_ values in the micromolar concentration range, making further studies into structure-transportability studies necessary to be able to even further reduce uptake of MC derivatives.

## Experimental Section

### Chemicals and Reagents

Azidopropylformamide (Azpfam) was provided by Prof. B. Westermann, Leibniz Institute for Plant Biochemistry (Halle, Germany). *p*-Azido-l-phenylalanine (Azphe), 2-(*R*)-amino-3-azidopropanoic acid hydrochloride (Azdala), 2-(*S*)-amino-3-azidopropanoic acid hydrochloride (Azlala), 4-azido-3-aminobutyric acid (Azaba), *N*^1^-azido-spermine hydrochloride (Azspe), and *N*^6^-[(2-Propyn-1-yloxy)carbonyl]-l-lysine (Prlys) were from BAPEKS Ltd. 4-Azido-d-alanine hydrochloride (Azhal), (2*S*, 3*S*)-2-amino-3-azido-l-butyric acid hydrochloride (Azabu), *N*-^ε^-azido-l-lysine hydrochloride (Aznle), azido-phenylpropionic acid (Azphepra), 14-azidomyristic acid (Azmya), and (2*S*)-6-(*tert*-butyloxycarbonyl)amino-2-azidohexanoic acid (Azaha(Boc)) were from Iris Biotech GmbH. 2-Azidoethanol (Azeol), 2-piperidinoethylazide (Azepip), and 5-chloro-1-pentyne (Clopyne) were from abcr GmbH. Carboxamide-propargyl biotin (Prbio) and biotin-PEG3-azide (Azbio) were from Lumiprobe GmbH. (2-Azidoethyl)dimethylamine (Azdmam), 3-azidopropane-1,2-diol (Azpdiol), 2-azido-2-methylpropanoic acid (Azmepra), and 2-azidopropanoic acid (Azpra) were from Enamine Ltd. Azidoacetic acid (Azaa) was from TCI Deutschland GmbH. Sodium ascorbate, tris(3-hydroxypropyltriazolylmethyl)amine (THPTA), and copper(II) sulfate were from Carl Roth GmbH + Co. KG. Propargylamine (Pram), 1-azido-4-chlorobenzene (Azclob), 3-azido-1-propanamine (Azpram), and aminoguanidine hydrochloride were from Merck KGaA. Acetonitrile (MeCN) in HPLC and MS purity grade as well as trifluoracetic acid (TFA) were from Honeywell Specialty Chemicals Seelze GmbH, water in MS purity grade was from AppliChem GmbH.

The Boc-group of Azaha(Boc) (20 mg, 0.973 mmol) was cleaved by adding 1 mL of 33% TFA in MeCN/water (4:1, v/v) and stirring at room temperature. After 2 h, the solvent was removed in vacuo yielding Azaha as a white solid (quant.).

### Modeling of Microcystins in the Binding Site of PP1

Co-crystallized ligands from PDB entries 2BDX and 6OBQ were modified using the Protein builder module of MOE 2022.02. For 2BDX, Ala^4^ was replaced by Arg^4^ following dehydration to build **1**. Subsequently, all MCs were manually built by extending or modifying atoms of **1** with subsequent local minimization of the modified parts within the protein environment using the MMFF94 force field.^69^

### Protein Preparation, Molecular Dynamics (MD) Simulations, and Dynophore Generation

PDB entry 2BDX(17) (resolution: 2.3 Å), containing PP1 with cocrystallized dihydromicrocystin-LA, and PDB entry 6OBQ(45) (resolution 1.84 Å), representing PP1 in complex with covalently bound **1**, were chosen as a basis for this study. Other publicly available crystal structures contained mutations with higher proximity to the MC binding site (6OBR, 6OBU). The PDB files were processed and curated in MOE (Molecular Operating Environment 2022.02; Chemical Computing Group ULC). Protonate3D^70^ was applied at pH 7.4. **2** to **5** were manually derived from the intermediately obtained representation of **1** by transforming monomers l-Leu^2^ or l-Arg^4^ into their respective counterparts in MOE (2BDX only). Subsequently, the newly generated derived azide or alkyne monomers underwent local energy minimization (using MMFF94).^69^

The resulting protein–ligand complexes were prepared with Maestro v. 13.1.137 (Schrödinger Release 2022-1: Maestro, Schrödinger LLC) for molecular dynamics (MD) simulations. Termini were capped and disulfide bonds were added. Complexes were embedded in cubic TIP3P^71^ water boxes with 15 Å extension. All systems were electrically neutralized and subsequently isotonized by addition of NaCl. All-atom MD simulations were performed using Desmond v. 6.9^72^ on water-cooled Nvidia RTX 2080 Ti GPUs using the OPLS-AA^73^ force field. Temperature was kept constant at 300 K and the pressure at 1.01325 bar via Nose–Hoover chain method and Martyna–Tobias–Klein method, respectively. All PP1–MC complexes were treated as flexible during simulations of 100 ns in three replicas amounting to an overall analysis time of 300 ns (time step 300 ps) for each MC structure. After postprocessing with VMD v. 1.9.3.^74^ and conversion to DCD format interaction frequencies occurring between protein and ligand were analyzed by our dynamic 3D pharmacophores application (dynophores).^50−54^ Coordinates of representative protein–ligand complexes were extracted after the first step of the MD simulation for visualization after full system equilibration in VMD v. 1.9.3.^74^

The protein structure of PP1 with cocrystallized and covalently bound **1** (PDB 6OBQ) was prepared in the same manner in MOE including reversal of the artificial mutation H66K and manual addition of one Mn^2+^ ion. The structure was used for the comparison of the intermolecular interactions before and after the formation of the covalent bond between Cys273 and Mdha^7^ as well as for the covalent docking.

### Covalent Molecular Docking

Covalent molecular docking was performed in MOE (Molecular Operating Environment 2022.02; Chemical Computing Group ULC) with the integrated covalent docking module. In preparation for the docking operation, the protein structure of MC-(Prtyr)R was computationally transformed into its corresponding unsubstituted MC-YR analogue. In the MC-YR structure, the l-Tyr^2^ O_η_ was manually selected to define the reactive site for the covalent docking operation on the protein side. The protein was prepared and minimized using MMFF94.^69^ In the covalent docking module, docking poses were created utilizing MOE’s “transformation placement” procedure, resulting in covalent bond formation. The ligands were treated as flexible and the protein as rigid. A maximum of 30 conformations per ligand were generated and scored using London dG. The docking solutions resulting from the placement step were refined by applying the default minimization procedure following the “Rigid Receptor” postprocessing scheme. All other standard settings were kept. The poses were inspected and analyzed in Ligandscout v. 4.4.3^75,76^ and minimized using the MMFF94 force field.^69^

### Production and Isolation of **2** to **5**

*Microcystis* sp. strains CBT 275, CBT 480, and CBT 633 were cultivated in 20 L polycarbonate carboys in standard BG11 medium and supplemented with 5% CO_2_ (1 L h^–1^).^77^ Fluorescent tubes (Sylvania GroLux, F18W/GRO) were used as light source, providing an average light intensity of 35 μE m^–2^ s^–1^. Cultivation temperature was 28 °C. Cultures were continually harvested and supplemented with 60 μM Prtyr (production of **2**; CBT 480) or Aznle (production of **4**, CBT 480), 50 μM Aznva (production of **3**; CBT 633) or 60 μM Aznle (production of **4**; CBT 633), or 60 μM Aznle (production of **5**, CBT 275) over several months. Between 0.4 and 0.7 g/L of dried biomass could be obtained every week from a 20 L culture. Freeze-dried biomasses were extracted with 80% MeOH/H_2_O (v/v) (60 mg dry biomass/mL) in two cycles consisting of sonication for 1 min with a sonotrode (100% power; 100% cycle duty) on ice, shaking at room temperature for 20 min, and centrifugation (13,000*g*; 20 min). After each cycle, the supernatant was collected, and fresh solvent was added to the pellet. The combined supernatants were concentrated to dryness in vacuo using a rotary evaporator. The resulting extracts were dissolved in 20% MeOH/H_2_O (v/v) (5 mg/mL), and loaded on C18 cartridges (Biotage Sfär C18 Duo, 100 Å, 30 μm). Subsequent to a washing step with one column volume 20% MeOH/H_2_O, fractionation was conducted by elution with 80% MeOH/H_2_O (v/v). The respective fraction containing the MC (monitored by HPLC) was dried in vacuo, redissolved in MeCN/H_2_O (80:20 (v/v) to a concentration of about 50 mg/mL, and subjected to semipreparative HPLC on an UltiMate 3000 (Thermo Fisher Scientific) using a Kinetex C18 column (5 μm, 100 Å, 150 × 10 mm, Phenomenex; **2**, **4**, **5**) or a Luna PFP column (5 μm, 100 Å, 250 × 10 mm, Phenomenex; **3**) and a linear gradient of aqueous MeCN with 0.1% TFA at 25 °C (**2**: 20% to 33% in 3 min, to 40% in 15 min, 5.0 mL/min, *t*_R_ 13.3–14.8 min; **3**: isocratic 36% for 16 min, 4.5 mL/min, *t*_R_ 12.9–14.0 min; **4**: 32% to 46% in 26 min, 5.0 mL/min, *t*_R_. 16.7–17.5 min; **5**: 20% to 38% in 3 min, to 40% in 14 min, to 55% in 0.1 min, to 56% in 26 min, flow 5.0 mL/min, *t*_R_ 23.6–24.2 min). Fractions containing the respective MC were combined and freeze-dried. On average, yields of clickable MCs ranged between 0.1 and 0.25% of cell dry weight. For NMR experiments, 5 mg of **5** were dissolved in 0.6 mL of DMSO-*d*_6_. Spectra were recorded using a Jeol ECZ600 spectrometer operating at 600 MHz (^1^H). NMR data were analyzed with Mnova 14.3.

### Derivatization of MCs via Copper-Catalyzed Azide–Alkyne Cycloaddition and Isolation of the Derivatives

Stock solutions of the clickable compounds listed in Table 1 were prepared in DMSO (100 mM) and stored at −20 °C. Stock solutions of **1** to **5** were dissolved in DMSO (10 mM) and stored at −20 °C. For the reaction, stock solution of copper(II) sulfate (100 mM in deionized water, stored at −20 °C) and THPTA (200 mM in deionized water, stored at −20 °C) were mixed in a ratio of 1:5 in deionized water. To prevent ascorbate byproducts, an aminoguanidine solution (100 mM in methanol, stored at 4 °C) was added to a final concentration of 5 mM. The optimal ratio of MC stock solution to clickable compound stock solution was 1:5. Sodium ascorbate solution (100 mM in deionized water, always prepared directly before use) was added to a concentration of 2.5 mM to start the reaction. The reaction mixture (final volume 1000 μL) was incubated at room temperature for at least 90 min. For the present study, 200 μM (about 200 μg) of each of the derivatized MCs **2a** to **5z** were synthesized.

After the reaction, the derivatized MCs were either purified using solid phase extraction (SPE) or HPLC. After activating a PolySpher RP18 cartridge (100 mg sorbent; Merck) with MeCN, equilibration with 5% MeCN in water, and sample loading, the system was washed with 5% and 10% MeCN in water. The MCs were eluted with 30%, 50% or 80% MeCN in water. HPLC isolation was performed on an UltiMate 3000 (Thermo Fisher Scientific) using a Kinetex C18 column (5 μm, 100 Å, 150 × 10 mm, Phenomenex). The MCs were eluted with a linear gradient of aqueous MeCN with 0.1% TFA, starting with either 25% MeCN, increasing to 50% MeCN in 18 min or starting with 30% MeCN, increasing to 100% MeCN in 18 min at 25 °C, followed by a plateau with 100% MeCN for 3 min, depending on the properties of the functionalized MCs. The purity after isolation of all MCs was ≥95% (HPLC-DAD, chromatographic conditions as described for quantification). The structures of the synthesized MC derivatives were confirmed by HPLC-HRMS/HRMS using a Q Exactive Plus mass spectrometer (Thermo Fisher Scientific) equipped with a heated ESI interface coupled to an UltiMate 3000 HPLC system (Thermo Fisher Scientific). Chromatographic conditions: aqueous MeCN with 0.1% FA, starting with 5% MeCN, increasing to 100% MeCN in 16 min, followed by a plateau with 100% MeCN for 4 min. Parameters for MS data acquisition: pos. ion mode, ESI spray voltage: 3.5 kV, resolution at *m*/*z* 200:280.000, scan range: *m*/*z* 150–2000.

### Quantification of Derivatized MCs

The concentration of the MC test solutions for the bioassays were quantified using HPLC-ELSD (Sedex 85, Sedere) as described previously.^42^ An analytical standard of MC-LR (10 μg/mL, Simris Biologics GmbH) was used to establish a calibration curve from 50 to 150 ng on-column. 5, 10, and 15 μL were injected in triplicate on a Kinetex C18 column (2.6 μm, 100 Å, 100 × 3 mm), and eluted with a gradient from 10–100% MeCN in H_2_O (0.1% TFA each) over 10 min at 0.65 mL/min. Settings of the ELSD were as follows: evaporation temperature 40 °C, gain 11, N_2_ pressure 3.5 bar. The calibration curve was generated as described by Adnani et al.^78^ In brief, the response areas were averaged, and log(ELSD response area) was plotted against log(amount in ng) to generate a linear calibration curve. The derivatized MCs were dissolved in 1 mL of MeOH and injected in duplicate under the same conditions.

### PP1 Inhibition Assay

As PP2A has not been commercially available while this study was conducted, the assay could only be performed with PP1, which was obtained from Merck KGaA. The assay was based on the procedure of Heresztyn et al.^79^ The final concentration of PP1 was changed to 0.8 units/mL. The sample or standard (4 μL) was combined with enzyme solution (4 μL) in a 384 well flat-bottomed microtiter plate. Each MC dilution was tested in quadruplicate. 40 μL of substrate solution was added to start the reaction. The final concentrations of MC dilutions were 0.001 nM to 1,000 nM. After 2 h, the absorbance was measured at 405 nm with an Infinite M200 Pro plate reader (Tecan Group AG). Data was analyzed with GraphPad Prism 6. Percentage of activity of PP1 was calculated as follows: PP1 activity (%) = ((Absorbance_sample_ – Absorbance_blank_)/(Absorbance_control_ – Absorbance_blank_)) × 100. A sigmoidal, four-parameter logistic curve, from which the IC_50_ were deduced, was fitted using the log_10_(*x*) values for the concentration. The model can also be described by *Y* = Bottom + (Top – Bottom)/(1 + 10^((LogIC_50_ – *X*) × HillSlope)^). Each measurement was repeated at least twice independently.

### Cell Culture and Cytotoxicity Assay

HEK293 cells, stably transfected with the expression vectors pcDNA3.1(+)-OATP1B1 and pcDNA3.1/Hygro(−)-OATP1B3, and the respective empty vectors pcDNA3.1(+) and pcDNA3.1/Hygro(−) as controls were provided by Prof. Dr. Joerg König (Friedrich-Alexander-Univeristät Erlangen-Nuernberg, Germany).^62^ All four cell lines were maintained in minimal essential medium, supplemented with 10% heat-inactivated fetal bovine serum, nonessential amino acid mix and 2 mM glutamine at 37 °C and 5% CO_2_, and routinely subcultured by trypsination. HEK293 OATP1B1+ and the corresponding control cell line were constantly selected with 800 μg/mL G418, while 250 μg/mL of hygromycin B were used for the selection of HEK293 OATP1B3+ and its empty vector control. Every 3 to 6 months, all cell lines were tested for mycoplasma contamination as described before.^80^ Roti-CELL glutamine solution was from Carl Roth GmbH + Co. KG. Hygromycin B solution was from InvivoGen. All other cell culture reagents were from Merck KGaA.

HEK293 OATP1B1 and control as well as OATP1B3 and control cells with a confluency of 70–90% were seeded with a concentration of 50,000 cells/well in 100 μL growth medium containing selection marker in uncoated 96-well flat plates. After 24 h incubation, sodium butyrate was added to a final concentration of 10 mM and the cells were incubated for another 24 h. The medium containing the inducer was removed and replaced with the MC dilutions. The MCs were diluted in growth medium to final concentrations of 0.01 μM to 3 μM per well. One % (v/v) DMSO was used as negative control. Actinomycin D with a final concentration of 50 μM was used as positive control. The dilutions were tested in triplicate and were incubated for 48 h. Afterward, the cells were fixed, washed, and stained as previously described,^81^ and the absorbance at 510 nm was measured with an Infinite M200 Pro plate reader. GraphPad Prism 6 was used to plot the data. Percentage of cell viability was calculated as follows: cell viability (%) = ((Absorbance_sample_ – Absorbance_blank_)/(Absorbance_control_ – Absorbance_blank_)) × 100. A sigmoidal, four-parameter logistic curve, from which the EC_50_ were deduced, was fitted using the log_10_(*x*) values for the concentration. The model can also be described by Y = Bottom + (Top – Bottom)/(1 + 10^((LogEC_50_ – *X*) × HillSlope)^). Each measurement was repeated at least twice independently.

## Data Availability

^1^H NMR data for **5** have been deposited at nmrXiv (DOI: 10.57992/nmrxiv.p81).

## Supporting Information Available

Additional data on the molecular modeling (relative frequencies of noncovalent interactions, interactions between MCs and PP1, dynophore representations), assay graphs, structures, chromatograms, HRMS, MS/MS spectra of all described MCs (PDF)
