# Supplementary material for: Derivatization of Microcystins Can Increase Target Inhibition while Reducing Cellular Uptake
Source: J Nat Prod. 2024 Oct 20;88(1):3–14. doi: 10.1021/acs.jnatprod.4c00688 (PMC11773564; doi:10.1021/acs.jnatprod.4c00688)
Supplement: Supplementary file 1 — np4c00688_si_001.pdf [file np4c00688_si_001.pdf]

Supporting Information

Derivatization of microcystins can increase target inhibition while reducing cellular uptake

Laura L. Sallandt[†⊥], *Clemens A. Wolf*[§], *Sabine Schuster*[‡⊥], *Heike Enke*[‡], *Dan Enke*[‡], *Gerhard Wolber*[§], *and Timo H. J. Niedermeyer*[†⊥,*]

[†]Department of Pharmaceutical Biology/Pharmacognosy, Institute of Pharmacy, Martin-Luther-University Halle-Wittenberg, 06120 Halle (Saale), Germany

[⊥]Department of Pharmaceutical Biology, Institute of Pharmacy, Freie Universität Berlin, 14195 Berlin, Germany

[§]Department of Pharmaceutical Chemistry, Institute of Pharmacy (Molecular Drug Design), Freie Universität Berlin, 14195 Berlin, Germany

[‡]Simris Biologics GmbH, 12489 Berlin, Germany

[*]Corresponding author

Table S1. Overview of shared interactions of the D-Masp[3], Adda[5], and D-Glu[6] residues of 1 with the protein phosphatase 1. 5
Table S2. Relative frequency of non-covalent interactions likely important for MCs 1-5 binding to the protein phosphatase 1 binding pocket. HBA – hydrogen bond between MC and protein with MC acting as acceptor. 6
Table S3. Overview over interactions of MC derivative substituents to PP residues present in the canyon-like binding pocket: Lys98, Asp71, Asn271, and Arg96. HBD = Hydrogen bond to protein with MC acting as donor. HBA = Hydrogen bond to protein with MC acting as acceptor. 7
Table S4. Annotation of the MS^2 spectrum of MC-(PrtyrAzLala)R (2a) from click reaction of 2 with azido-L-alanine (a). 14
Table S5. Detailed evaluation of the MS/MS spectrum of 3. Fragments indicated in bold are only explainable if the clickable amino acid is incorporated in the postulated position of the MC core structure. 37
Table S6. Detailed evaluation of the MS/MS spectrum of 4. Fragments indicated in bold are only explainable if the clickable amino acid is incorporated in the postulated position of the MC core structure. 44
Table S7. Detailed evaluation of the MS/MS spectrum of 5. Fragments indicated in bold are only explainable if the clickable amino acid is incorporated in the postulated position of the MC core structure. 51

Figure S1. Dynamic pharmacophore (dynophore) representations of the interactions between 1 before (A) and after (B) formations of the covalent bond between Cys273 and Mdha[7] in their PP1 binding site. 2 (C), 3 (D), 4 (E), and 5 (F) in their PP1 binding site. Each dot represents a spatiotemporal occurrence of an interaction over the course of the simulation. Additional sub-pocket in protein conformations 2BDX (G) and 6OBQ (G). 9
Figure S2. Graphs of PPI (two independent experiments, n=4 each) and cytotoxicity assays (two independent experiments, n=3 each) of MCs 1 – 5 11
Figure S3. Structure, MS, and MS^2 data of 2. Key fragment b5/y5 (Mdha-Ala-Prtyr-Masp-Arg) *m/z* 641.3029 (0.0010 Da). HPLC-DAD chromatogram at 210 nm. 12
Figure S4. Structure, MS, and MS^2 data of 2a. Key fragment b5/y5 (Mdha-Ala-PrtyrAzLala-Masp-Arg) *m/z* 771.3531 (0.0002 Da). HPLC-DAD chromatogram at 210 nm. 13
Figure S5. Structure, MS, and MS^2 data of 2b. Key fragment b5/y5 (Mdha-Ala-PrtyrAzDala-Masp-Arg) *m/z* 771.3521 (0.0011 Da). HPLC-DAD chromatogram at 210 nm. 15
Figure S6. Structure, MS, and MS^2 data of 2c. Key fragment b5/y5 (Mdha-Ala-PrtyrAzabu-Masp-Arg) *m/z* 785.3688 (0.0001 Da). HPLC-DAD chromatogram at 210 nm. 16
Figure S7. Structure, MS, and MS^2 data of 2d. Key fragment b5/y5 (Mdha-Ala-PrtyrAzhal-Masp-Arg) *m/z* 785.3659 (0.0030 Da). HPLC-DAD chromatogram at 210 nm. 17
Figure S8. Structure, MS, and MS^2 data of 2e. Key fragment b5/y5 (Mdha-Ala-PrtyrAznle-Masp-Arg) *m/z* 813.3998 (0.0004 Da). HPLC-DAD chromatogram at 210 nm. 18
Figure S9. Structure, MS, and MS^2 data of 2f. Key fragment b5/y5 (Mdha-Ala-PrtyrAzphe-Masp-Arg) *m/z* 847.3868 (0.0022 Da). HPLC-DAD chromatogram at 210 nm. 19
Figure S10. Structure, MS, and MS^2 data of 2g. Key fragment b5/y5 (Mdha-Ala-PrtyrAzaba-Masp-Arg) *m/z* 785.3714 (0.0025 Da). HPLC-DAD chromatogram at 210 nm. 20
Figure S11. Structure, MS, and MS^2 data of 2h. Key fragment b5/y5 (Mdha-Ala-PrtyrAzaha-Masp-Arg) *m/z* 813.3951 (0.0051 Da). HPLC-DAD chromatogram at 210 nm. 21

Figure S12. Structure, MS, and MS^2 data of 2i. Key fragment b5/y5 (Mdha-Ala-PrtyrAzpram-Masp-Arg) *m/z* 741.3840 (0.0049 Da). HPLC-DAD chromatogram at 210 nm. 22
Figure S13. Structure, MS, and MS^2 data of 2j. Key fragment b5/y5 (Mdha-Ala-PrtyrAzspe-Masp-Arg) *m/z* 869.5078 (0.0026 Da). HPLC-DAD chromatogram at 210 nm. 23
Figure S14. Structure, MS, and MS^2 data of 2k. Key fragment b5/y5 (Mdha-Ala-PrtyrAzdmam-Masp-Arg) *m/z* 755.3942 (0.0006 Da). HPLC-DAD chromatogram at 210 nm. 24
Figure S15. Structure, MS, and MS^2 data of 2l. Key fragment b5/y5 (Mdha-Ala-PrtyrAzepip-Masp-Arg) *m/z* 795.4245 (0.0015 Da). HPLC-DAD chromatogram at 210 nm. 25
Figure S16. Structure, MS, and MS^2 data of 2m. Key fragment b5/y5 (Mdha-Ala-PrtyrAzacac-Masp-Arg) *m/z* 742.3231 (0.0036 Da). HPLC-DAD chromatogram at 210 nm. 26
Figure S17. Structure, MS, and MS^2 data of 2n. Key fragment b5/y5 (Mdha-Ala-PrtyrAzpra-Masp-Arg) *m/z* 756.3420 (0.0004 Da). HPLC-DAD chromatogram at 210 nm. 27
Figure S18. Structure, MS, and MS^2 data of 2o. Key fragment b5/y5 (Mdha-Ala-PrtyrAzmepra-Masp-Arg) *m/z* 770.3531 (0.0049 Da). HPLC-DAD chromatogram at 210 nm. 28
Figure S19. Structure, MS, and MS^2 data of 2p. Key fragment b5/y5 (Mdha-Ala-PrtyrAzphepra-Masp-Arg) *m/z* 832.3671 (0.0066 Da). HPLC-DAD chromatogram at 210 nm. 29
Figure S20. Structure, MS, and MS^2 data of 2q. Key fragment b5/y5 (Mdha-Ala-PrtyrAzmya-Masp-Arg) *m/z* 910.5182 (0.0037 Da). HPLC-DAD chromatogram at 210 nm. 30
Figure S21. Structure, MS, and MS^2 data of 2r. Key fragment b5/y5 (Mdha-Ala-PrtyrAzpfam-Masp-Arg) *m/z* 769.3718 (0.0022 Da). HPLC-DAD chromatogram at 210 nm. 31
Figure S22. Structure, MS, and MS^2 data of 2s. Key fragment b5/y5 (Mdha-Ala-PrtyrAzeol-Masp-Arg) *m/z* 728.3457 (0.0017 Da). HPLC-DAD chromatogram at 210 nm. 32
Figure S23. Structure, MS, and MS^2 data of 2t. Key fragment b5/y5 (Mdha-Ala-PrtyrAzdiol-Masp-Arg) *m/z* 758.3531 (0.0049 Da). HPLC-DAD chromatogram at 210 nm. 33
Figure S24. Structure, MS, and MS^2 data of 2u. Key fragment b5/y5 (Mdha-Ala-PrtyrAzclob-Masp-Arg) *m/z* 794.3157 (0.0021 Da). HPLC-DAD chromatogram at 210 nm. 34
Figure S25. Structure, MS, and MS^2 data of 2v. Key fragment b5/y5 (Mdha-Ala-PrtyrAzbio-Masp-Arg) *m/z* 1041.4919 (0.0015 Da). HPLC-DAD chromatogram at 210 nm.................... 35
Figure S26. Structure, MS, and MS^2 data of 3. Key fragment b5/y5 – N_2 (Mdha-Ala-Aznva-Masp-Arg) *m/z* 552.2902 (0.0014 Da). HPLC-DAD chromatogram at 210 nm. 36
Figure S27. Structure, MS, and MS^2 data of 3w. Key fragment b5/y5 (Mdha-Ala-Aznvaprbio-Masp-Arg) *m/z* 861.4111 (0.0037 Da). HPLC-DAD chromatogram at 210 nm. 39
Figure S28. Structure, MS, and MS^2 data of 3x. Key fragment b5/y5 (Mdha-Ala-Aznvachlopyne-Masp-Arg) *m/z* 682.3156 (0.0030 Da). HPLC-DAD chromatogram at 210 nm. 40
Figure S29. Structure, MS, and MS^2 data of 3y. Key fragment b5/y5 (Mdha-Ala-Aznvapram-Masp-Arg) *m/z* 635.3378 (0.0006 Da). HPLC-DAD chromatogram at 210 nm. 41
Figure S30. Structure, MS, and MS^2 data of 3z. Key fragment b5/y5 (Mdha-Ala-Aznvaprlys-Masp-Arg) *m/z* 808.4102 (0.0042 Da). HPLC-DAD chromatogram at 210 nm. 42
Figure S31. Structure, MS, and MS^2 data of 4. Key fragment b5/y5 (Mdha-Ala-Aznle-Masp-Arg) *m/z* 594.3151 (0.0045 Da). HPLC-DAD chromatogram at 210 nm. 43
Figure S32. Structure, MS, and MS^2 data of 4w. Key fragment b5/y5 (Mdha-Ala-AznlePrbio-Masp-Arg) *m/z* 875.4282 (0.0022 Da). HPLC-DAD chromatogram at 210 nm. 46
Figure S33. Structure, MS, and MS^2 data of 4x. Key fragment b5/y5 (Mdha-Ala-AznleChlopyne-Masp-Arg) *m/z* 696.3376 (0.0033 Da). HPLC-DAD chromatogram at 210 nm. 47
Figure S34. Structure, MS, and MS^2 data of 4y. Key fragment b5/y5 (Mdha-Ala-AznlePram-Masp-Arg) *m/z* 649.3531 (0.0056 Da). HPLC-DAD chromatogram at 210 nm. 48

Figure S35. Structure, MS, and MS^2 data of 4z. Key fragment b5/y5 (Mdha-Ala-AznlePrlys-Masp-Arg) *m/z* 822.4296 (0.0080 Da). HPLC-DAD chromatogram at 210 nm. 49
Figure S36. Structure, MS, and MS^2 data of 5. Key fragment b5/y5 (Mdha-Ala-Leu-Masp-Aznle) *m/z* 551.2916 (0.0020 Da). HPLC-DAD chromatogram at 210 nm. 50
Figure S37. ^1H NMR spectrum of 5 at 600 MHz (DMSO). 53
Figure S38. Structure, MS, and MS^2 data of 5w. Key fragment b5/y5 (Mdha-Ala-Leu-Masp-AznlePrbio) *m/z* 832.4133 (0.0001 Da). HPLC-DAD chromatogram at 210 nm. 54
Figure S39. Structure, MS, and MS^2 data of 5x. Key fragment b5/y5 (Mdha-Ala-Leu-Masp-AznleChlopyne) *m/z* 653.3165 (0.0008 Da). HPLC-DAD chromatogram at 210 nm. 55
Figure S40. Structure, MS, and MS^2 data of 5y. Key fragment b5/y5 (Mdha-Ala-Leu-Masp-AznlePram) *m/z* 606.3354 (0.0004 Da). HPLC-DAD chromatogram at 210 nm. 56
Figure S41. Structure, MS, and MS^2 data of 5z. Key fragment b5/y5 (Mdha-Ala-Leu-Masp-AznlePrlys) *m/z* 696.3661 (0.0014 Da). HPLC-DAD chromatogram at 210 nm. 57

Table S1. Overview of shared interactions of the D-Masp[3], Adda[5], and D-Glu[6] residues of **1** with the protein phosphatase 1.

	D-Masp[3]	Adda[5]	D-Glu[6]
Ionic interactions (anionic)	Carboxylate moiety to Arg96		Carboxylate moiety to Arg96 or Mn^{2+}
Hydrogen bonds (acceptors)	Carboxylate oxygen atoms to Arg96 N_{η} or Tyr134 O_{η}		Carboxylate oxygen atoms to Tyr272 Oη, Arg96 N_{ε} or N_{η}, or Asn124 N_{δ}
Hydrophobic contacts		6-Methyl group to Trp206 or Ile130<br>8-Methyl group to Val223<br>Phenyl group to Val223, Tryp206, Ile130, or Val195	

Table S2. Relative frequency of non-covalent interactions likely important for MCs **1**-**5** binding to the protein phosphatase 1 binding pocket. HBA – hydrogen bond between MC and protein with MC acting as acceptor.

Interaction	**1**	**2**	**3**	**4**	**5**
HBA from carboxylate O1 of D-MeAsp[3]	94 %	96 %	97 %	82 %	86 %
HBA from carboxylate O2 of D-MeAsp[3]	91 %	95 %	95 %	94 %	91 %
Ionic interaction by carboxylate of D-MeAsp[3]	100 %	100 %	100 %	100 %	93 %
HBA from carboxylate O1 of D-Glu[6]	100 %	98 %	100 %	94 %	95 %
HBA from carboxylate O2 of D-Glu[6]	96 %	82 %	98%	76 %	95 %
Ionic interaction by carboxylate of D-Glu[6]	100 %	100 %	100 %	100 %	100 %
Hydrophobic contact by 6-methyl group of Adda[5]	99 %	99 %	99 %	99 %	100 %
Hydrophobic contact by 8-methyl group of Adda[5]	93 %	95 %	92%	92 %	85 %
Hydrophobic contact by phenyl group of Adda[5]	98 %	99 %	100 %	100 %	99 %

Table S3. Overview over interactions of MC derivative substituents to PP residues present in the canyon-like binding pocket: Lys98, Asp71, Asn271, and Arg96. HBD = Hydrogen bond to protein with MC acting as donor. HBA = Hydrogen bond to protein with MC acting as acceptor.

	Structure 2BDX		Structure 6OBQ		
	Lys98	Arg96	Lys98	Asp71	Asn271
**2a**	Ionic bond	HBD to backbone CO	Ionic bond<br>HBA to N_ζ	Ionic bond	HBD to backbone CO
**2b**	Ionic bond	HBD to backbone CO	Ionic bond	Ionic bond	HBD to backbone CO
**2c**	Ionic bond and HBA to N_ζ		Ionic bond	-	HBD to backbone CO
**2d**	Ionic bond	HBD to backbone CO	Ionic bond	Ionic bond<br>HBD to O_δ	-
**2e**	Ionic bond and HBA to N_α	-	Ionic bond<br>HBA to N_ζ	Ionic bond<br>HBD to O_δ	-
**2f**	Ionic bond<br>HBA to N_ζ	-	Ionic bond<br>HBA to N_ζ<br>Pi-cation interaction	Ionic bond<br>HBD to O_δ	-
**2g**	Ionic bond	HBD to backbone CO	Ionic bond<br>HBA to $N\alpha$, HBA to N_ζ	-	HBD to backbone CO
**2h**	Ionic bond	-	Ionic bond<br>HBA to $N\alpha$, HBA to N_ζ	Ionic bond<br>HBD to O_δ	-
**2i**	-	HBD to backbone CO	-	Ionic bond<br>HBD to O_δ	-
**2j**	-	HBD to backbone CO	-	Ionic bond<br>HBD to O_δ	-
**2k**	-	HBD to backbone CO	-	Ionic bond	-
**2l**	-	HBD to backbone CO	-	Ionic bond	-
**2m**	Ionic bond<br>HBA to N_ζ	-	Ionic bond<br>HBA to N_α, HBA to N_ζ	-	-
**2n**	Ionic bond<br>HBA to N_ζ	-	Ionic bond<br>HBA to N_α, HBA to N_ζ	-	-
**2o**	Ionic bond<br>HBA to N_ζ	-	Ionic bond<br>HBA to N_α, HBA to N_ζ	-	-
**2p**	Ionic bond<br>HBA to N_α	-	Ionic bond<br>HBA to N_ζ	-	-
**2q**	-	-	-	-	-
**2r**	HBA to N_α	HBD to backbone CO	HBA to N_ζ	-	-

**2s**	-	HBD to backbone CO	HBA to N_ζ	-	-
**2t**	-	HBD to backbone CO	HBA to N_ζ	-	-
**2u**	Hydrophobic contacts to alkyl chain of Lys	-	Pi-cation interaction	-	-
**2v**	HBA to N_ζ	-	2 HBA interactions to N_ζ	-	-

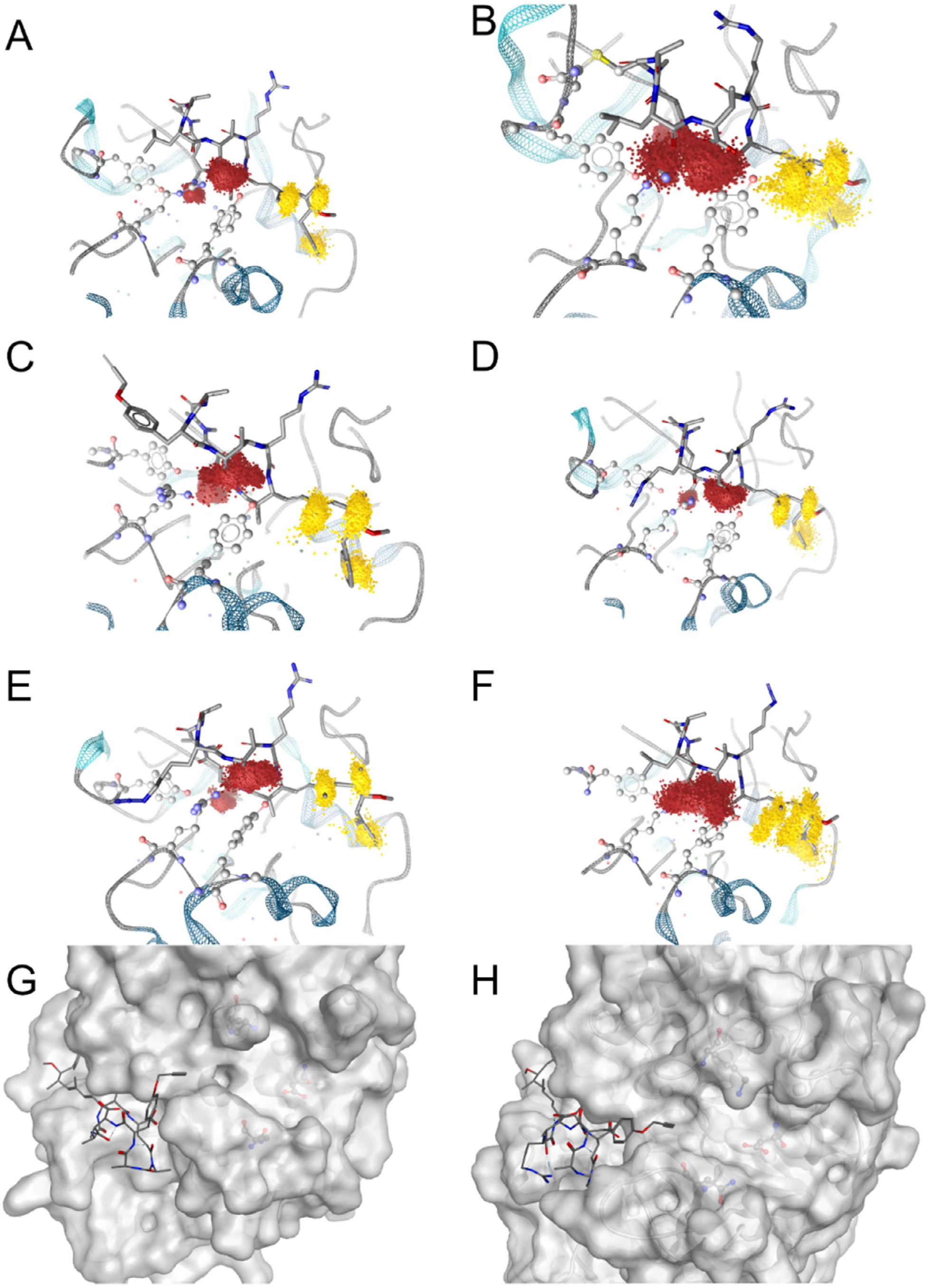

Figure S1. Dynamic pharmacophore (dynophore) representations of the interactions between **1** before (A) and after (B) formations of the covalent bond between Cys273 and Mdha[7] in their PP1 binding site. **2** (C), **3** (D), **4** (E), and **5** (F) in their PP1 binding site. Each dot represents a spatiotemporal occurrence of an interaction over the course of the simulation. Additional sub-pocket in protein conformations 2BDX (G) and 6OBQ (G).

<table>
<tr><th></th><th>PPI</th><th>Cytotoxicity (OATP1B1) assay</th><th>Cytotoxicity (OATP1B3) assay</th></tr>
<tr><td>underivatized</td>
<td>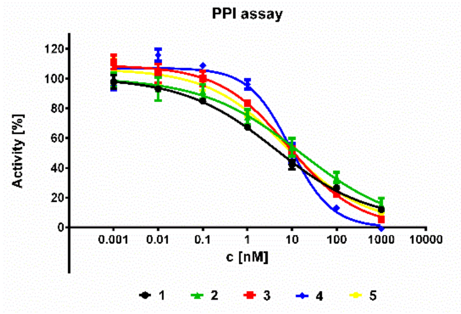
</td>
<td>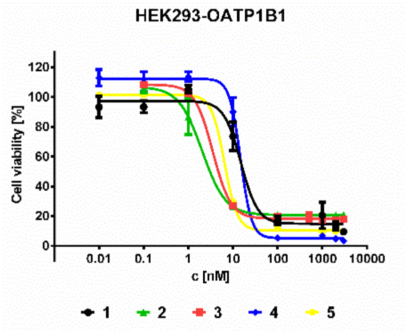
</td>
<td>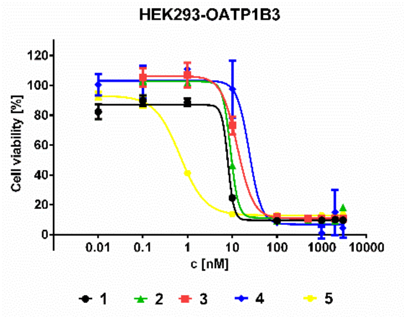
</td></tr>
<tr><td>Amino acids</td>
<td>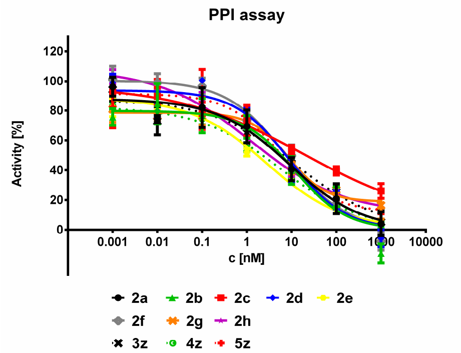
</td>
<td>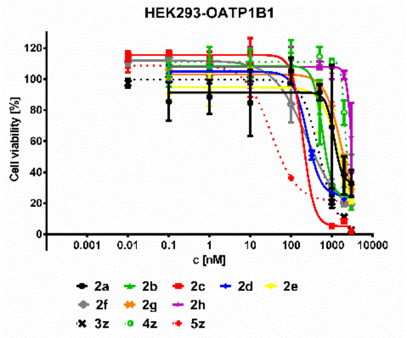
</td>
<td>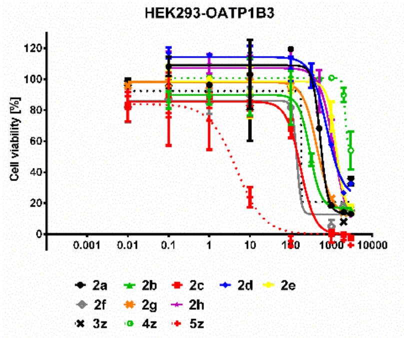
</td></tr>
<tr><td>Amines</td>
<td>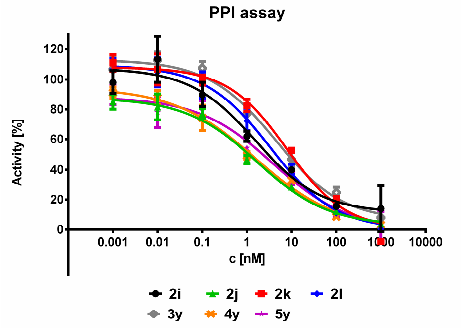
</td>
<td>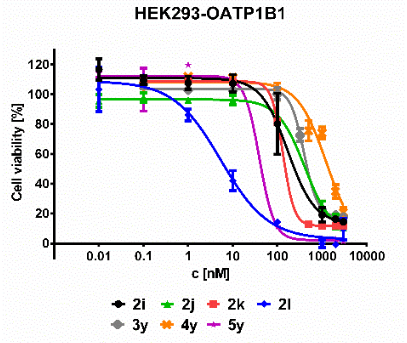
</td>
<td>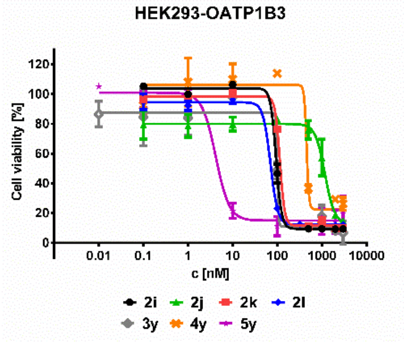
</td></tr>
</table>

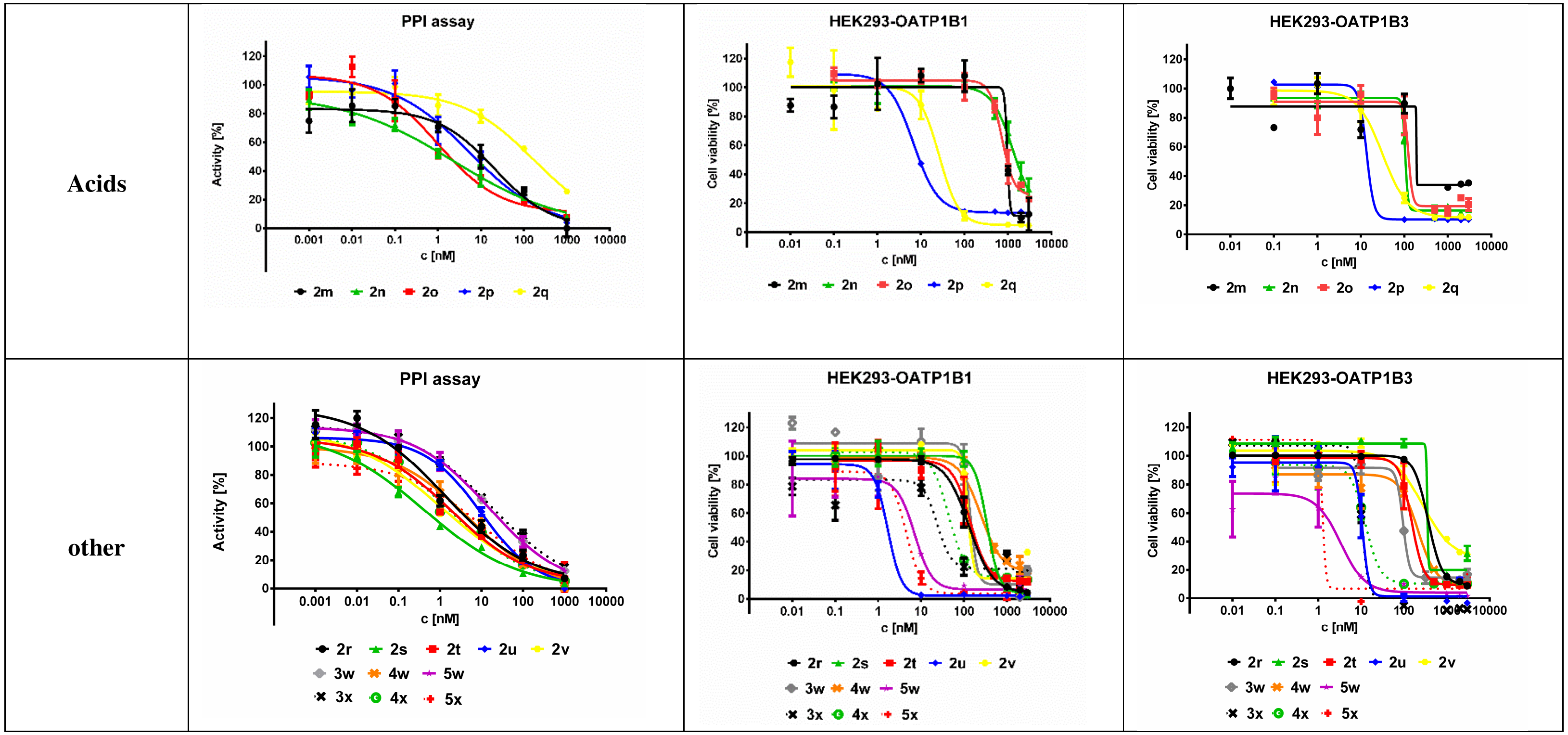

Figure S2. Graphs of PPI (two independent experiments, n=4 each) and cytotoxicity assays (two independent experiments, n=3 each) of MCs **1** – **5** (**1**: black circle, **2**: green triangle, **3**: red square, **4**: blue diamond, **5**: yellow hexagon), **2a** – **2v** (**2a**: black circle, **2b**: green triangle, **2c**: red square, **2d**: blue diamond, **2e**: yellow hexagon, **2f**: hollow grey diamond, **2g**: orange cross, **2h**: purple star, **2i**: black circle, **2j**: green triangle, **2k**: red square, **2l**: blue diamond, **2m**: black circle, **2n**: green triangle, **2o**: red square, **2p**: blue diamond, **2q**: yellow hexagon, **2r**: black circle, **2s**: green triangle, **2t**: red square, **2u**: blue diamond, **2v**: yellow hexagon), **3w** – **3z** (**3w**: hollow grey diamond, **3x**: dotted, black cross, **3y**: hollow grey diamond, **3z**: dotted, black cross), **4w** – **4z** (**4w**: hollow grey diamond, **4x**: dotted, black cross, **4y**: hollow grey diamond, **4z**: dotted, green hollow circle), and **5w** – **5z** (**5w**: purple star, **5x**: dotted, red cross, **5y**: purple star, **5z**: dotted, red cross)

Microcystin-[Propargyltyrosine]R (2)

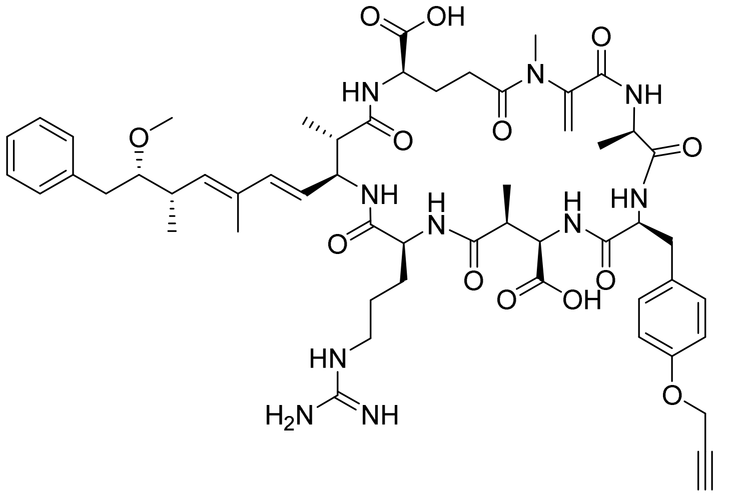

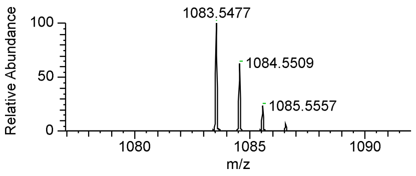

Sum formula

$C_{55}H_{74}N_{10}O_{13}$

pred. ***m/z*** **([M + H]⁺)**

1083.5510

meas. ***m/z*** **([M + H]⁺)**

1083.5477 (Δ 3.0 ppm)

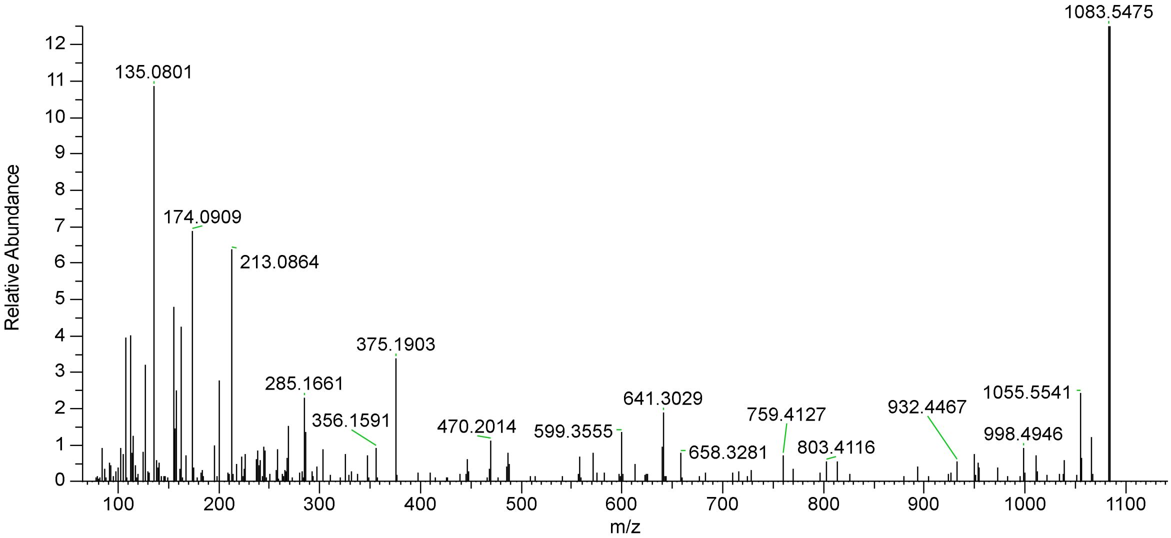

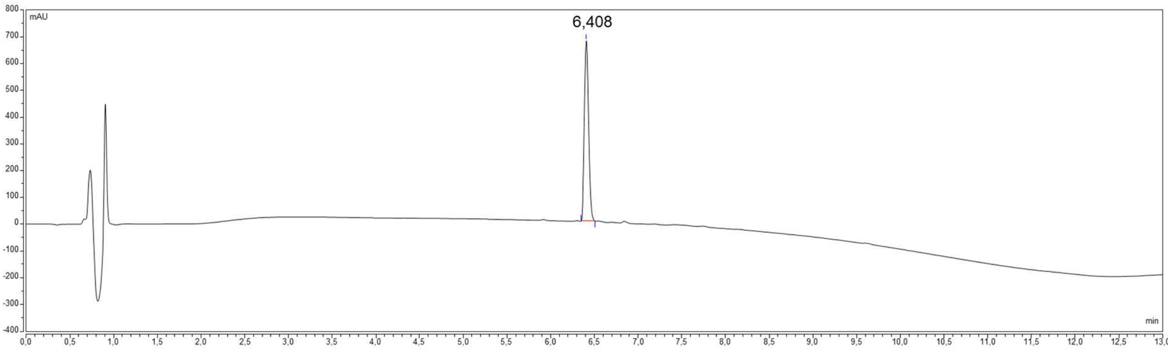

Figure S3. Structure, MS, and MS[2] data of **2**. Key fragment b5/y5 (Mdha-Ala-Prtyr-Masp-Arg) *m/z* 641.3029 (0.0010 Da). HPLC-DAD chromatogram at 210 nm.

Microcystin-[Propargyltyrosine | azido-L-alanine]R (2a)

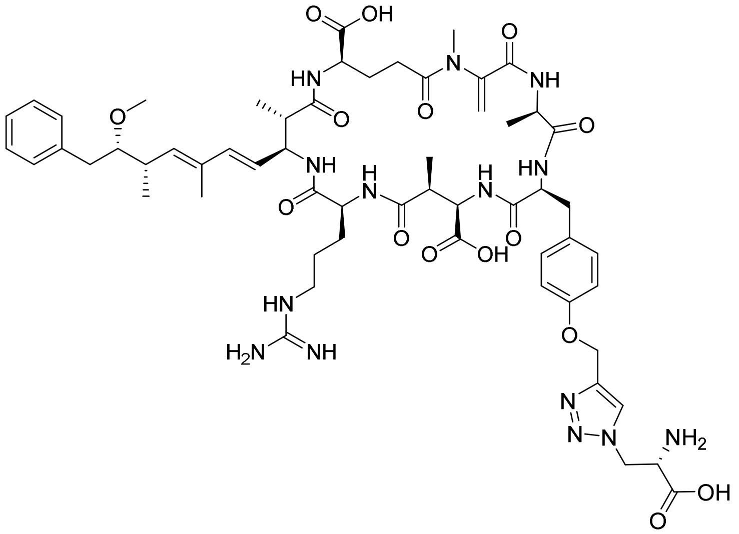

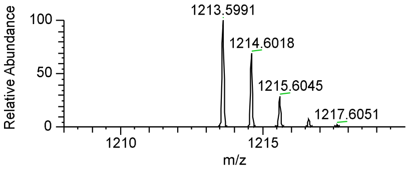

Sum formula

$C_{58}H_{80}N_{14}O_{15}$

pred. ***m/z*** **([M + H]$^+$)**

1213.6000

meas. ***m/z*** **([M + H]$^+$)**

1213.5989 (Δ 0.9 ppm)

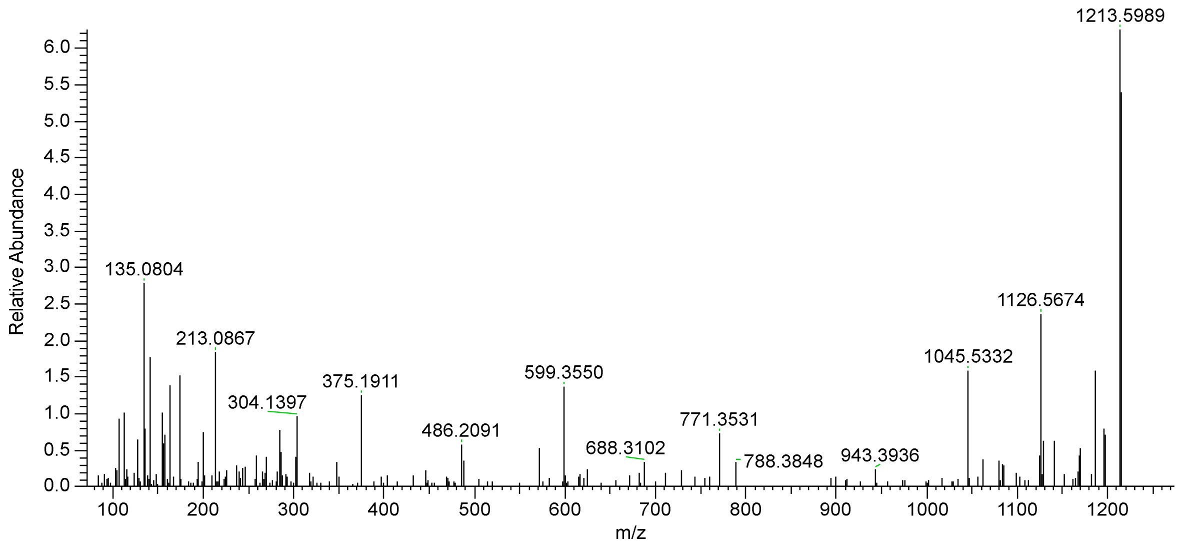

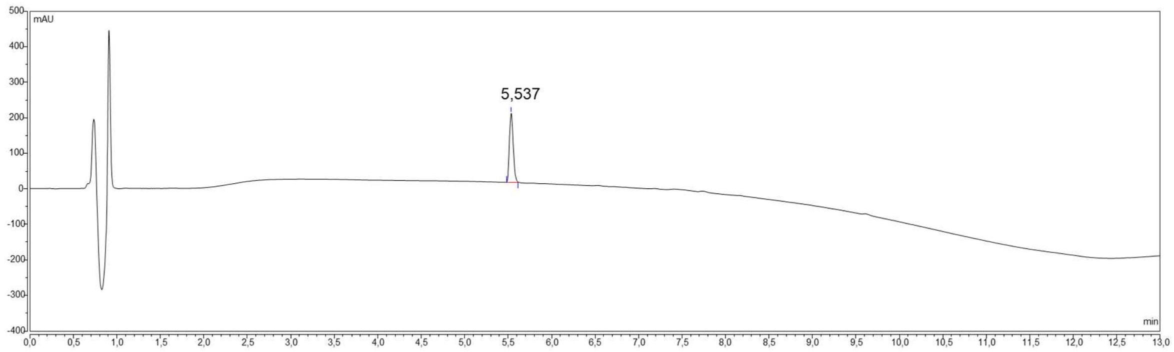

Figure S4. Structure, MS, and MS^2 data of **2a.** Key fragment b5/y5 (Mdha-Ala-PrtyrAzLala-Masp-Arg) *m/z* 771.3531 (0.0002 Da). HPLC-DAD chromatogram at 210 nm.

Table S4. Annotation of the MS^2 spectrum of MC-(PrtyrAzLala)R (**2a**) from click reaction of **2** with azido-L-alanine (**a**).

m/z	**annotation**	**ion**	**difference [Da]**
1213.5989	M + H$^+$	-	0.0012
1196.5753	M + H$^+$ - NH_3	-	0.0018
1195.5824	M + H$^+$ - H_2O	-	0.0071
1112.5585	Ala-PrtyrAzLala-Masp-Arg-Adda-Glu + H$^+$ - H_2O	b6/y6	0.0061
1084.5469	Mdha-Ala-PrtyrAzLala-Masp-Arg-Adda + H$^+$ or Arg-Adda-Glu-Mdha-Ala- PrtyrAzLala + H$^+$	b6/y6	0.0106
771.3531	Mdha-Ala-PrtyrAzLala-Masp-Arg + H$^+$	b5/y5	0.0002
710.3862	Masp-Arg-Adda-Glu + H$^+$ - H_2O	b4/y4	0.0010
688.3102	Ala-PrtyrAzLala-Masp-Arg + H$^+$	b4/y4	0.0006
682.3825	Arg-Adda-Glu-Mdha + H$^+$	b4/y4	0.0002
671.2884	Ala-PrtyrAzLala-Masp-Arg + H$^+$ - NH_3	b4/y4	0.0012
617.2823	PrtyrAzLala-Masp-Arg + H$^+$	b3/y3	0.0033
615.2575	Mdha-Ala-PrtyrAzLala-Masp + H$^+$ or Glu-Mdha-Ala- PrtyrAzLala + H$^+$	b4/y4	0.0054
600.2568	PrtyrAzLala-Masp-Arg + H$^+$ - NH_3	b3/y3	0.0044
599.3550	Arg-Adda-Glu + H$^+$ or Masp-Arg-Adda + H$^+$	b3/y3	0.0002
486.2091	Mdha-Ala- PrtyrAzLala + H$^+$	b3/y3	0.0004
470.3168	Arg-Adda + H$^+$	b2/y2	0.0043
403.1756	Ala-PrtyrAzLala + H$^+$	b2/y2	0.0032
375.1911	(Adda-$C_9H_{10}O$)-Glu-Mdha + H$^+$	z3	0.0034
286.1505	Masp-Arg + H$^+$	b2/y2	0.0005
269.1250	Masp-Arg + H$^+$ - NH_3	b2/y2	0.0006
213.0867	Glu-Mdha + H$^+$	b2/y2	0.0003
195.0762	Glu-Mdha + H$^+$ - H_2O	b2/y2	0.0002
157.1083	Arg + H$^+$	y1	0.0001
155.0815	Mdha-Ala + H$^+$	b2/y2	0.0001
140.0817	Arg + H$^+$ - NH_3	y1	0.0001
112.0402	Glu + H$^+$ - H_2O	y1	0.0009
84.0452	Mdha + H$^+$	y1	0.0008

Microcystin-[Propargyltyrosine | azido-D-alanine]R (2b)

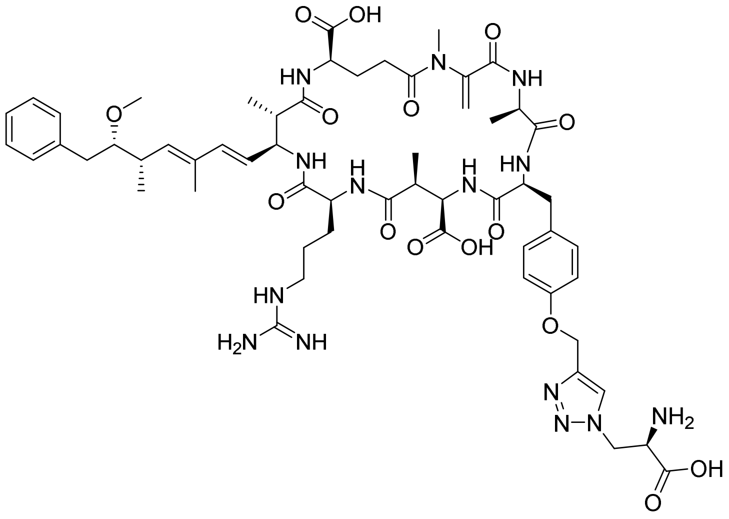

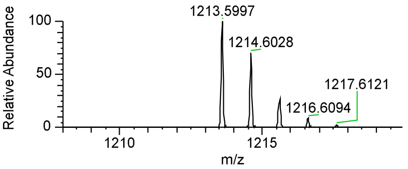

Sum formula

$C_{58}H_{80}N_{14}O_{15}$

pred. ***m/z*** **([M + H]⁺)**

1213.6000

meas. ***m/z*** **([M + H]⁺)**

1213.5997 (Δ 0.2 ppm)

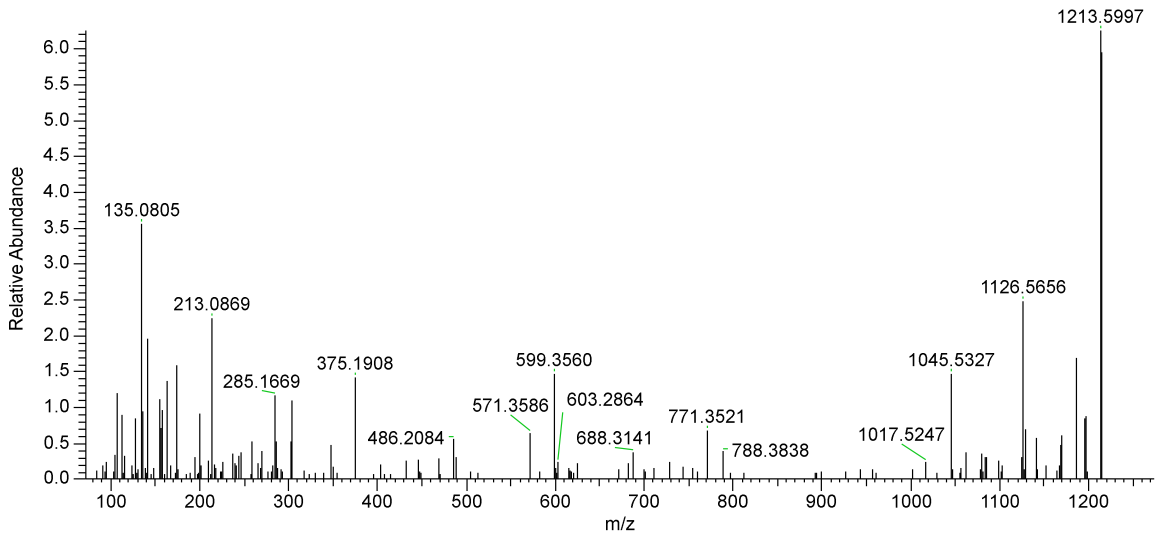

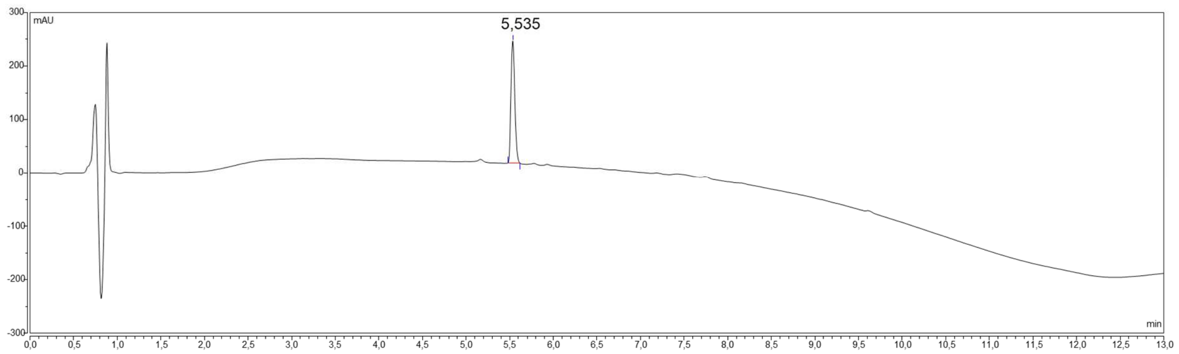

Figure S5. Structure, MS, and MS^2 data of **2b**. Key fragment b5/y5 (Mdha-Ala-PrtyrAzDala-Masp-Arg) *m/z* 771.3521 (0.0011 Da). HPLC-DAD chromatogram at 210 nm.

Microcystin-[Propargyltyrosine | 2-amino-3-azido-butanoic acid]R (2c)

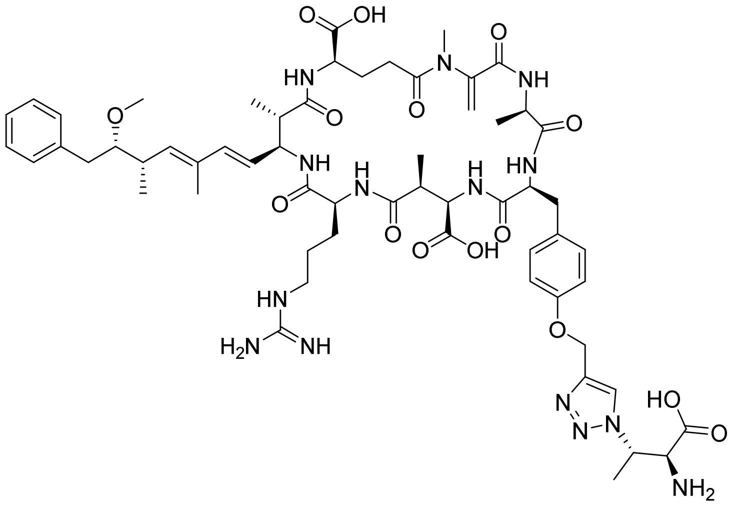

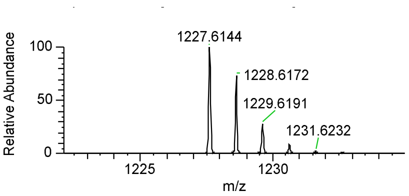

Sum formula

$C_{59}H_{82}N_{14}O_{15}$

pred. ***m/z*** **([M + H]⁺)**

1227.6157

meas. ***m/z*** **([M + H]⁺)**

1227.6145 (Δ 1.0 ppm)

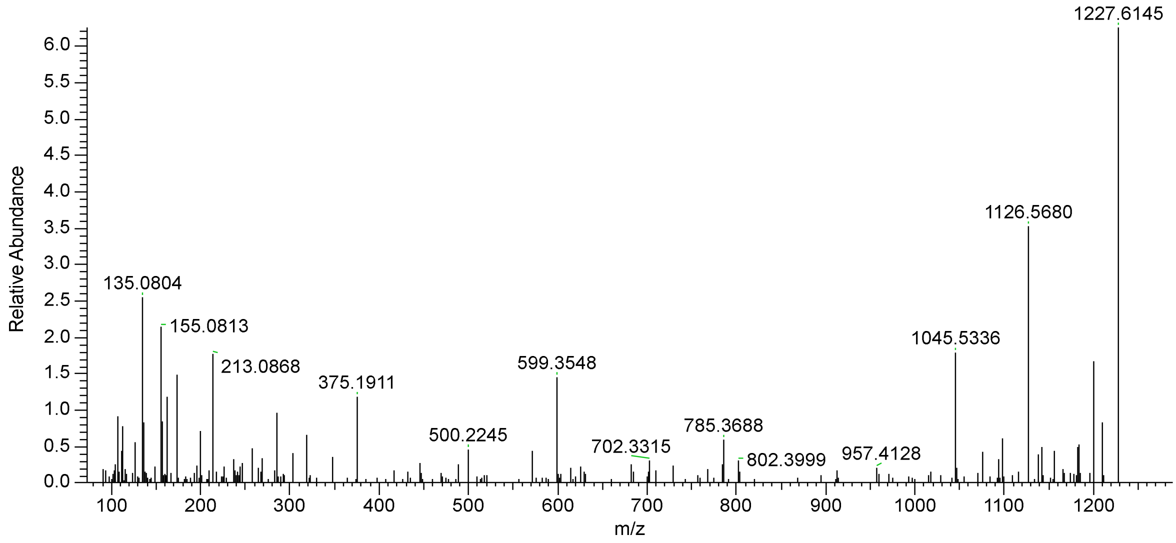

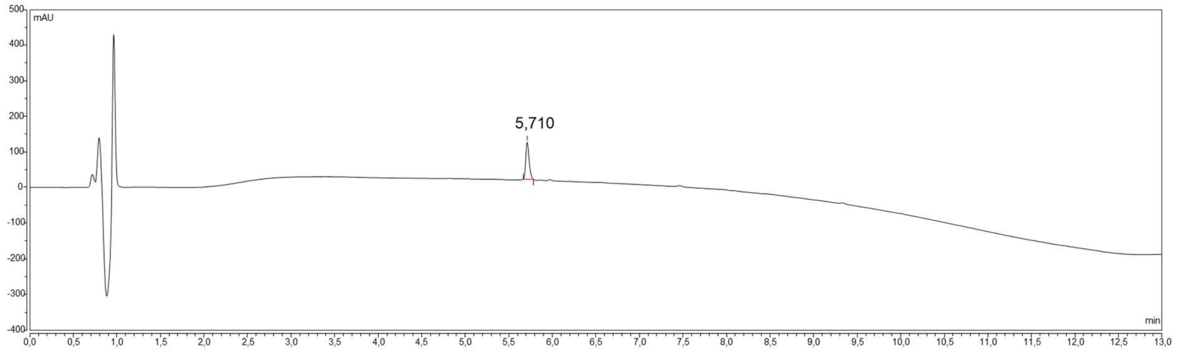

Figure S6. Structure, MS, and MS^2 data of **2c**. Key fragment b5/y5 (Mdha-Ala-PrtyrAzabu-Masp-Arg) *m/z* 785.3688 (0.0001 Da). HPLC-DAD chromatogram at 210 nm.

Microcystin-[Propargyltyrosine | azido-D-homoalanine]R (2d)

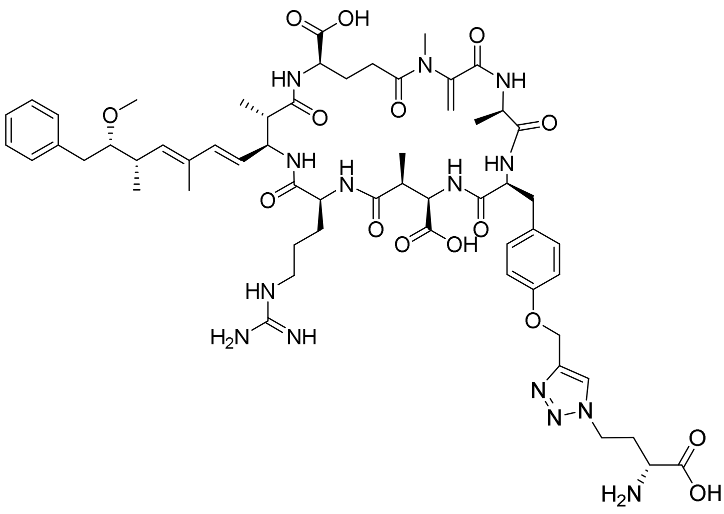

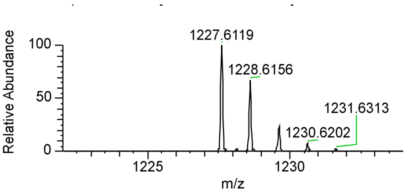

Sum formula

$C_{59}H_{82}N_{14}O_{15}$

pred. ***m/z*** **([M + H]⁺)**

1227.6157

meas. ***m/z*** **([M + H]⁺)**

1227.6119 (Δ 3.1 ppm)

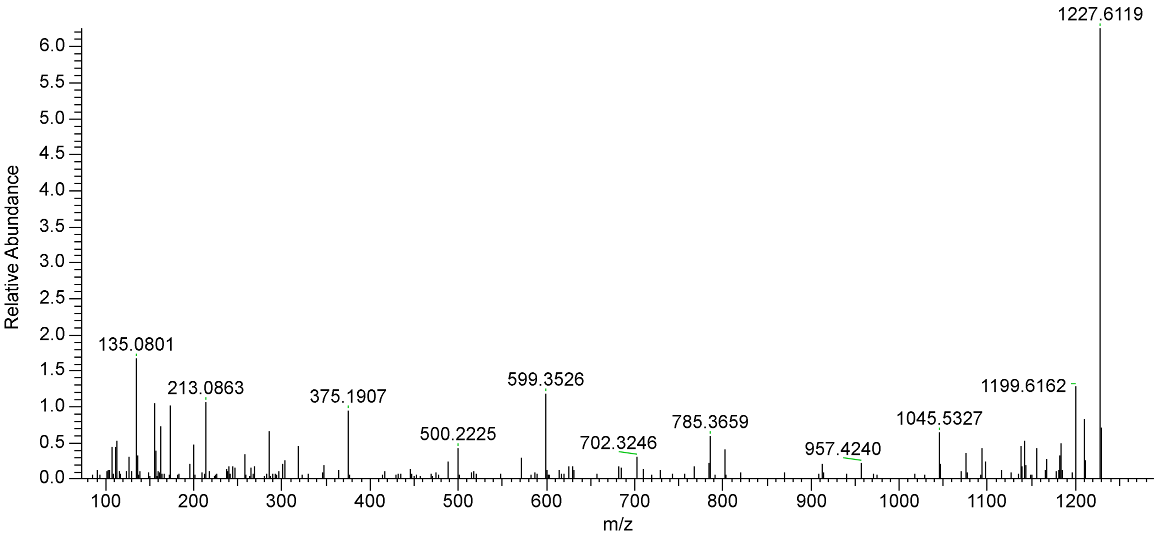

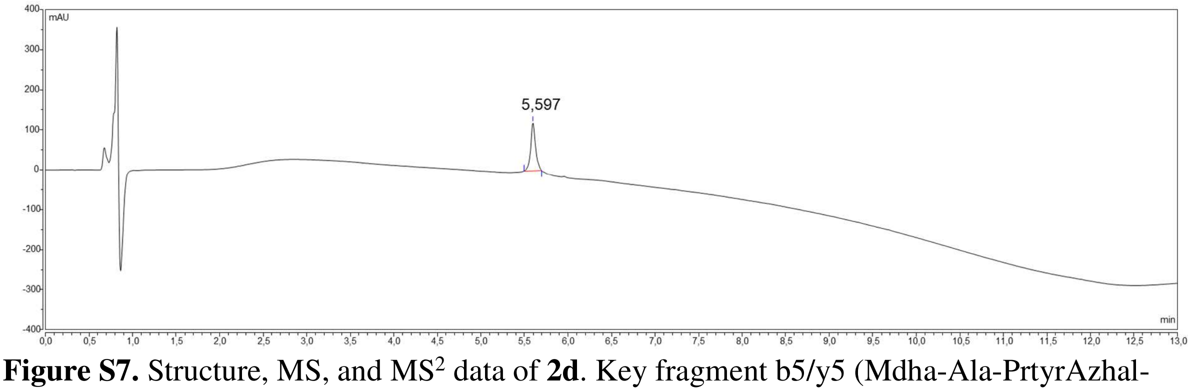

Figure S7. Structure, MS, and MS² data of **2d**. Key fragment b5/y5 (Mdha-Ala-PrtyrAzhal-Masp-Arg) *m/z* 785.3659 (0.0030 Da). HPLC-DAD chromatogram at 210 nm.

Microcystin-[Propargyltyrosine | azidonorleucine]R (2e)

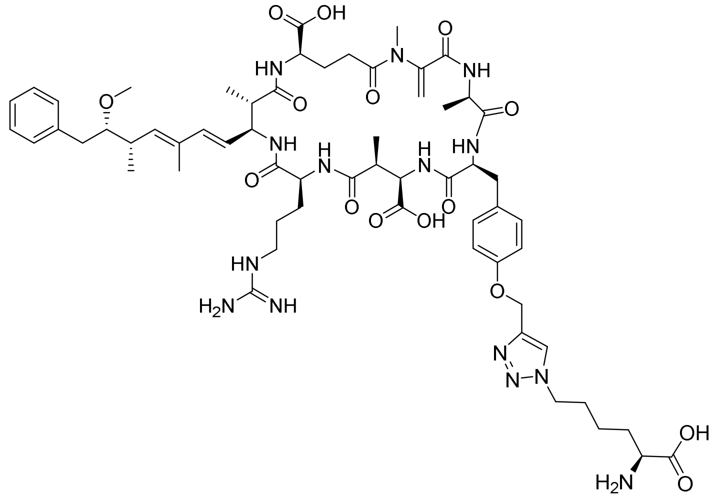

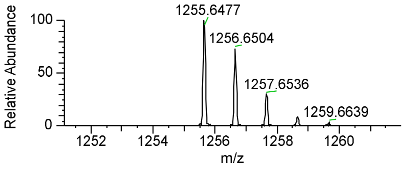

Sum formula

$C_{61}H_{86}N_{14}O_{15}$

pred. *m/z* ([M + H]$^+$)

1255.6470

meas. *m/z* ([M + H]$^+$)

1255.6477 (Δ 0.6 ppm)

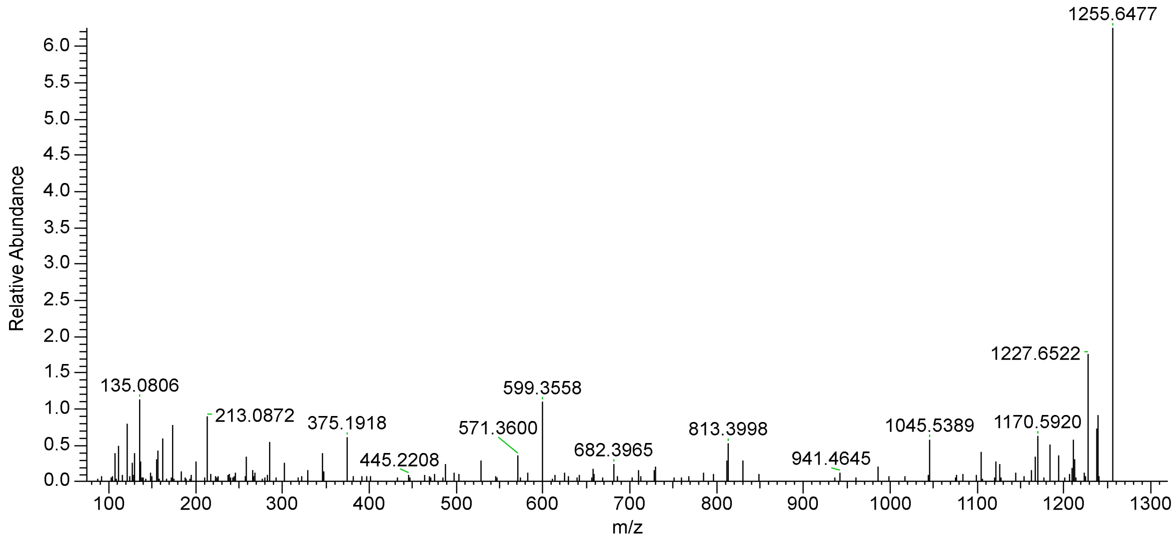

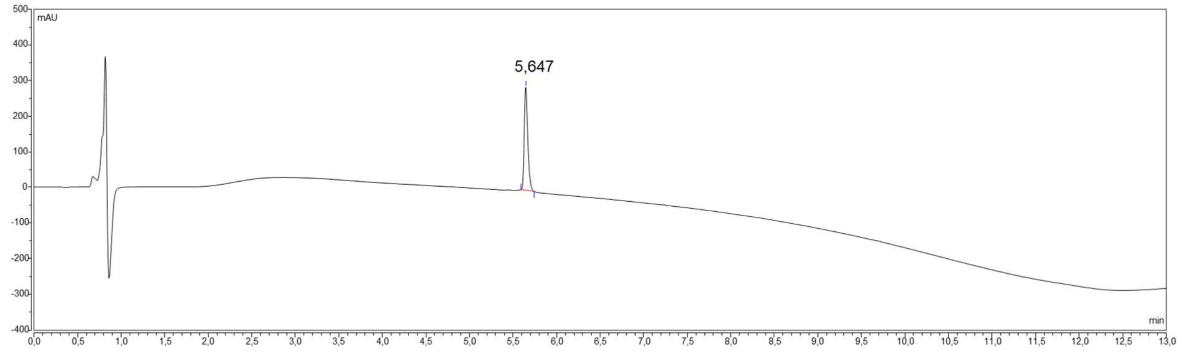

Figure S8. Structure, MS, and MS^2 data of **2e**. Key fragment b5/y5 (Mdha-Ala-PrtyrAznle-Masp-Arg) *m/z* 813.3998 (0.0004 Da). HPLC-DAD chromatogram at 210 nm.

Microcystin-[Propargyltyrosine | azidophenylalanine]R (2f))

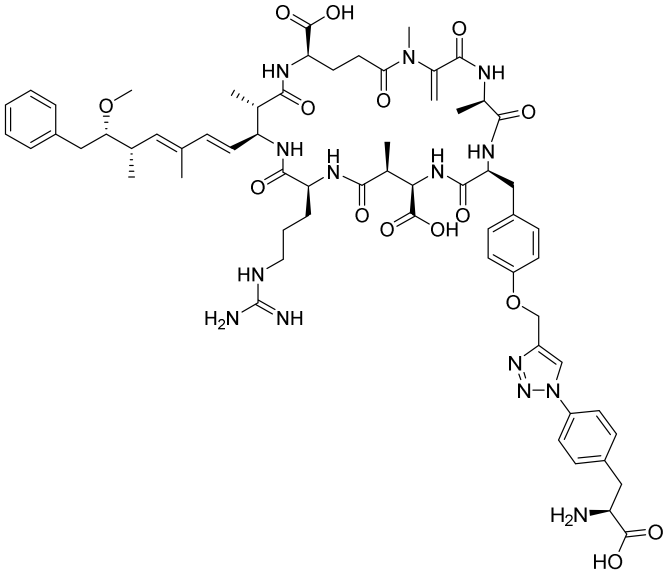

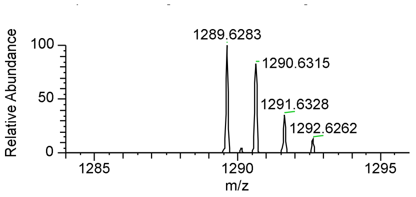

Sum formula

$C_{64}H_{84}N_{14}O_{15}$

pred. ***m/z*** **([M + H]$^+$)**

1289.6313

meas. ***m/z*** **([M + H]$^+$)**

1289.6283(Δ 2.3 ppm)

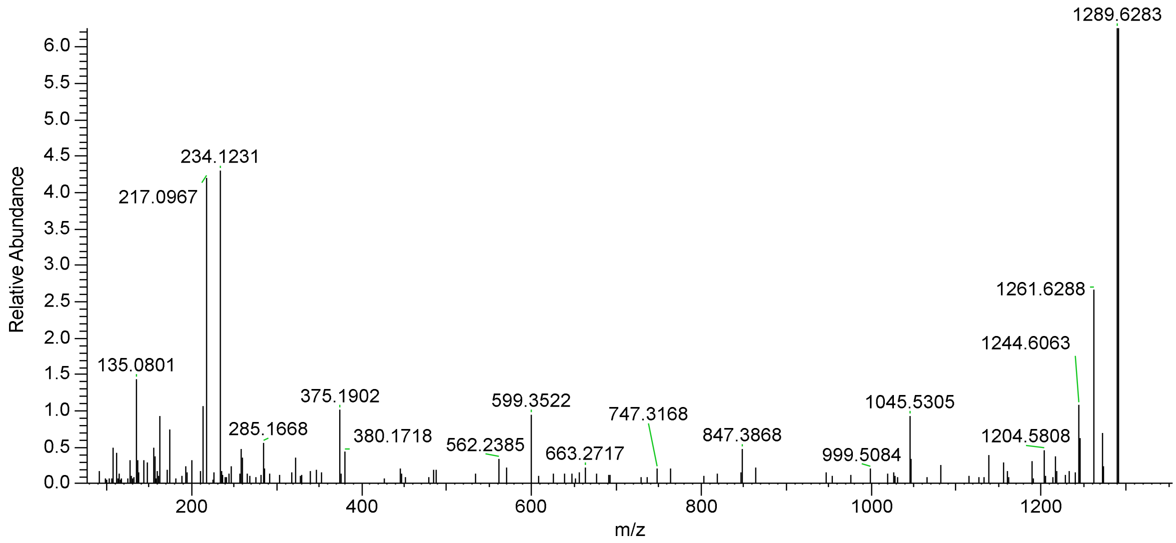

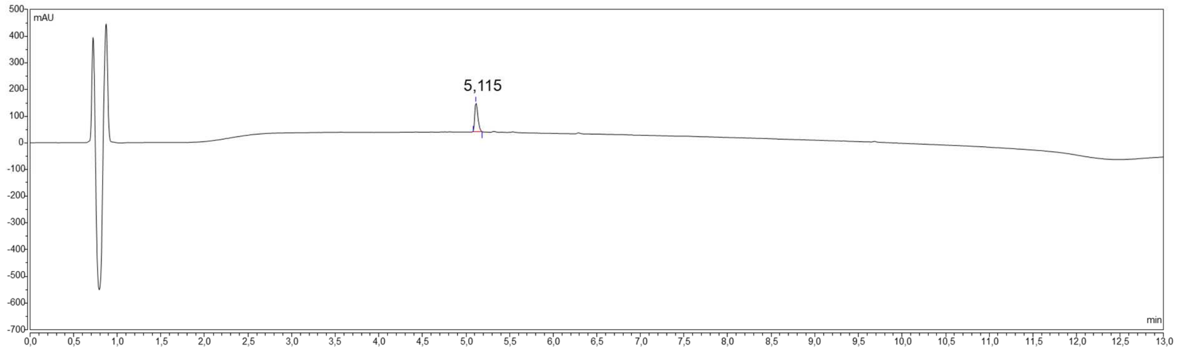

Figure S9. Structure, MS, and MS^2 data of **2f**. Key fragment b5/y5 (Mdha-Ala-PrtyrAzphe-Masp-Arg) *m/z* 847.3868 (0.0022 Da). HPLC-DAD chromatogram at 210 nm.

Microcystin-[Propargyltyrosine|3-amino-4-azidobutanoic acid]R (2g)

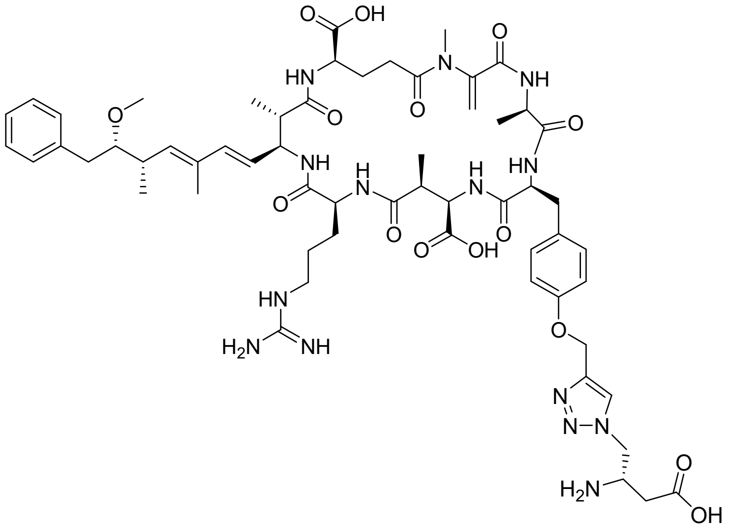

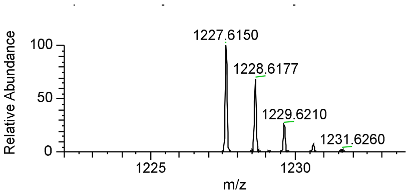

Sum formula

$C_{59}H_{82}N_{14}O_{15}$

pred. ***m/z*** **([M + H]$^+$)**

1227.6157

meas. ***m/z*** **([M + H]$^+$)**

1227.6150 (Δ 0.6 ppm)

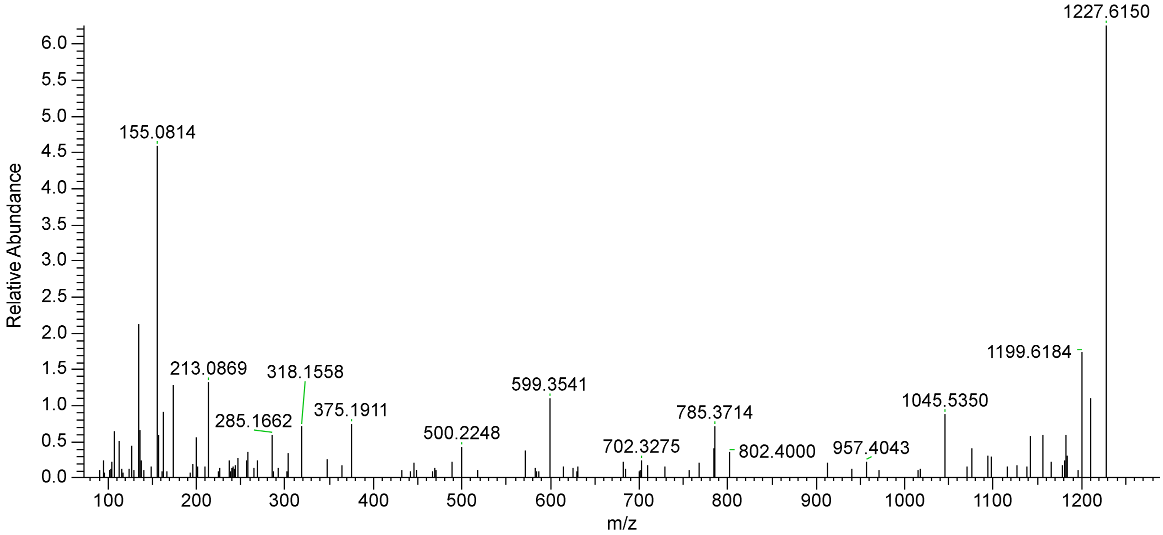

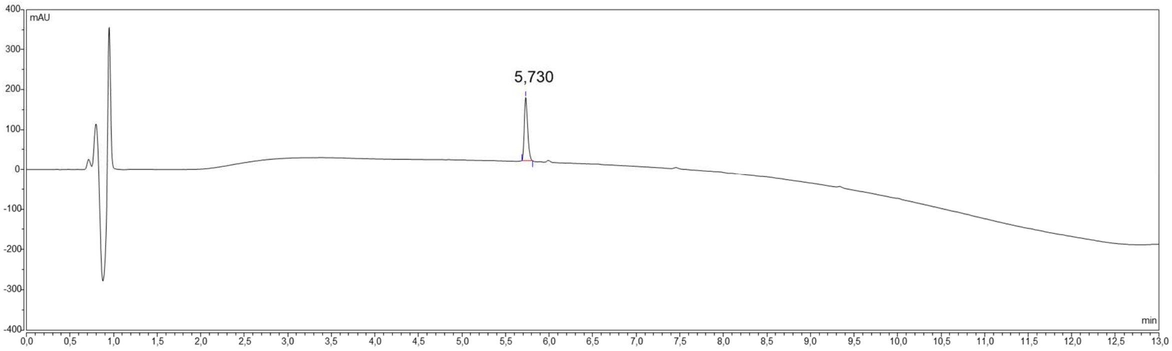

Figure S10. Structure, MS, and MS^2 data of **2g**. Key fragment b5/y5 (Mdha-Ala-PrtyrAzaba-Masp-Arg) *m/z* 785.3714 (0.0025 Da). HPLC-DAD chromatogram at 210 nm.

Microcystin-[Propargyltyrosine | (2*S*)-6-Amino-2-azidohexanoic acid]R (2h)

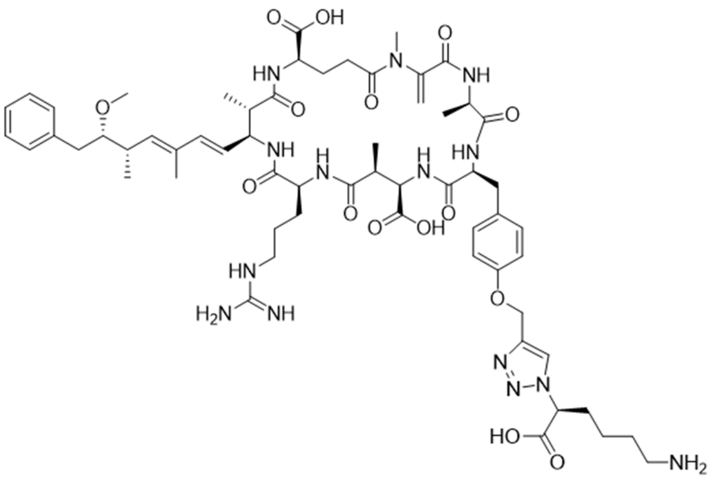

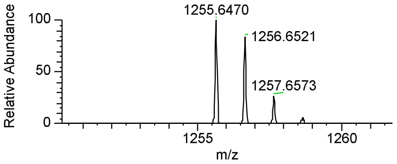

Sum formula

$C_{61}H_{86}N_{14}O_{15}$

pred. ***m/z*** **([M + H]$^{+}$)**

1255.6470

meas. ***m/z*** **([M + H]$^{+}$)**

1255.6470 (Δ 0.0 ppm)

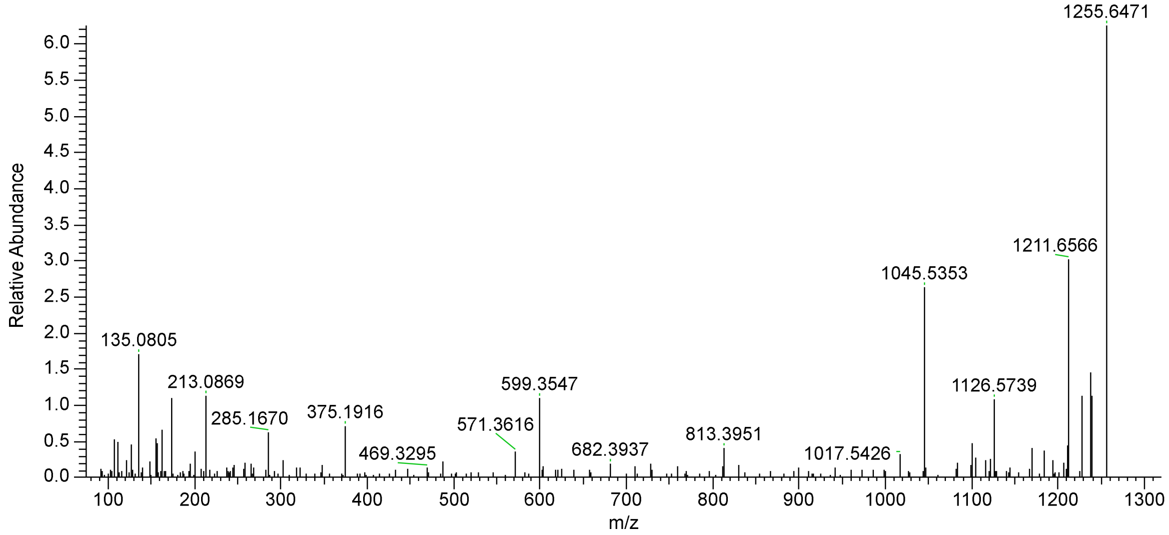

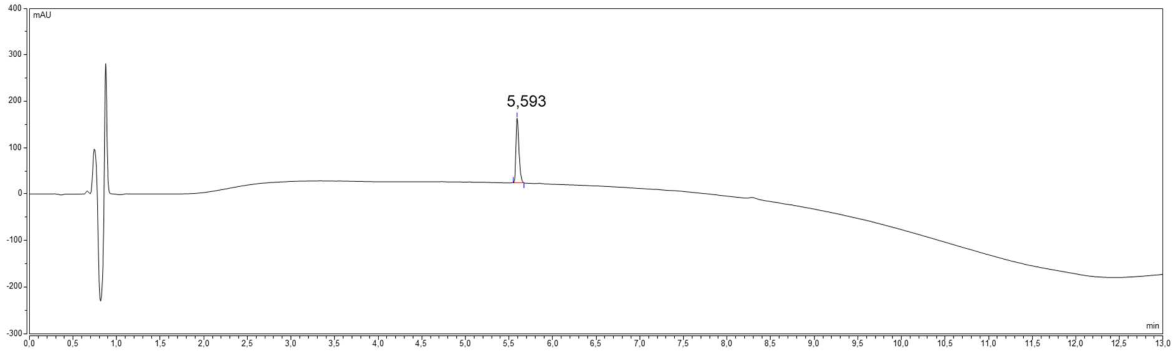

Figure S11. Structure, MS, and MS^2 data of **2h**. Key fragment b5/y5 (Mdha-Ala-PrtyrAzaha-Masp-Arg) *m/z* 813.3951 (0.0051 Da). HPLC-DAD chromatogram at 210 nm.

Microcystin-[Propargyltyrosine | 3-azidopropan-1-amine]R (2i)

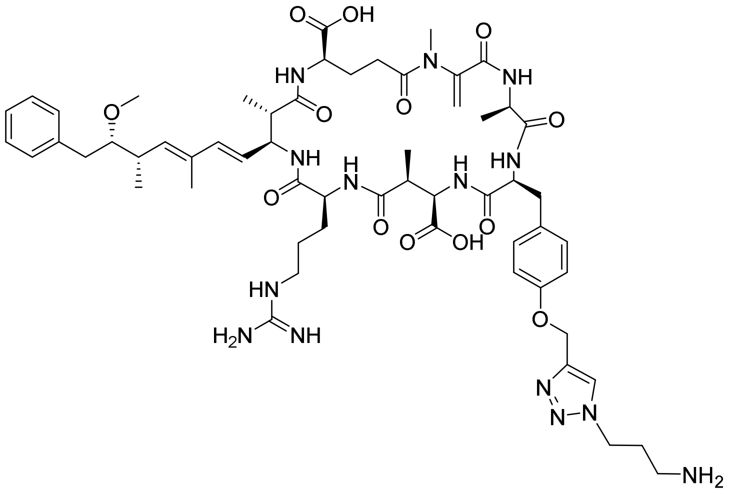

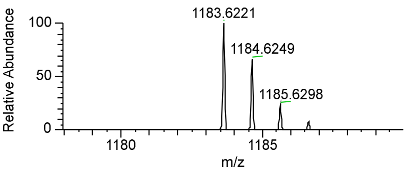

Sum formula

$C_{58}H_{82}N_{14}O_{13}$

pred. ***m/z*** **([M + H]⁺)**

1183.6259

meas. ***m/z*** **([M + H]⁺)**

1183.6222 (Δ 3.1 ppm)

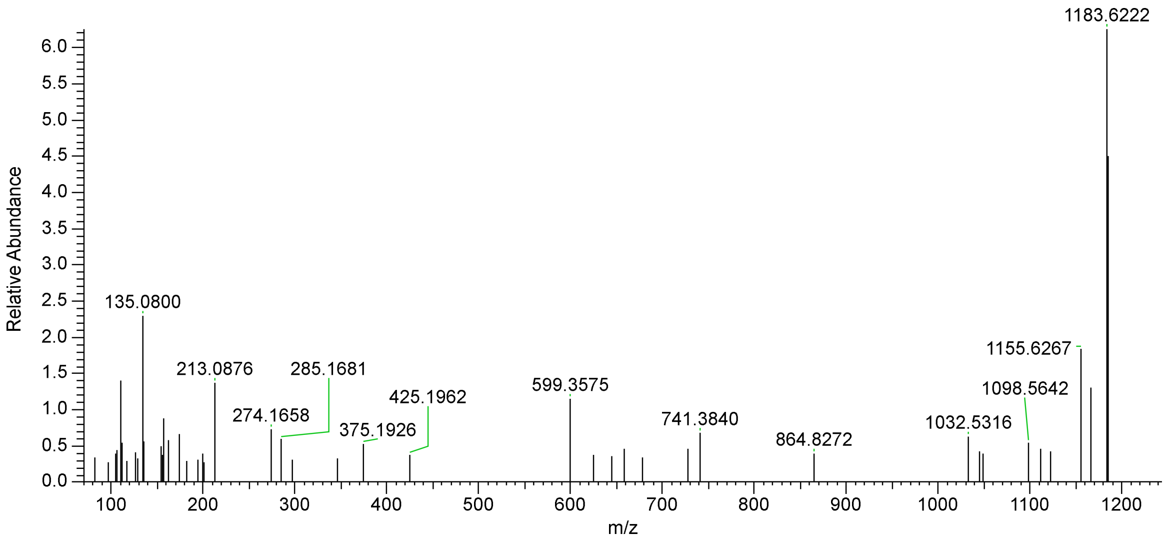

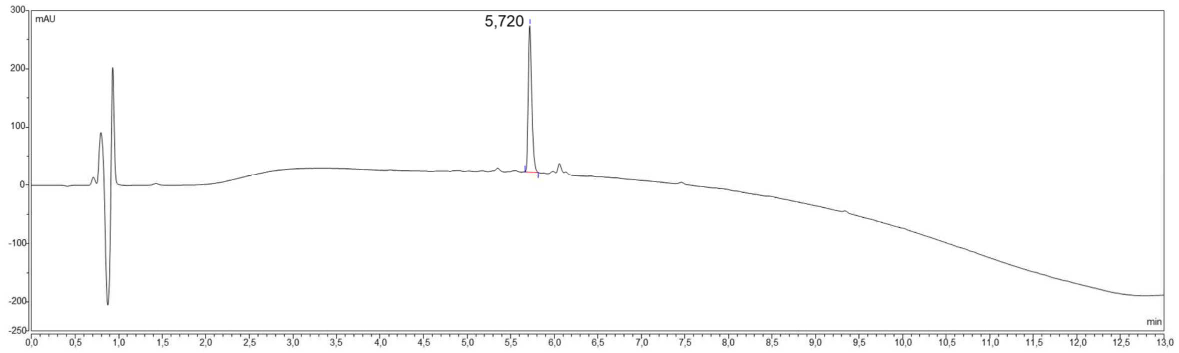

Figure S12. Structure, MS, and MS² data of **2i**. Key fragment b5/y5 (Mdha-Ala-PrtyrAzpram-Masp-Arg) *m/z* 741.3840 (0.0049 Da). HPLC-DAD chromatogram at 210 nm.

Microcystin-[Propargyltyrosine | *N*1-azidospermine]R (2j)

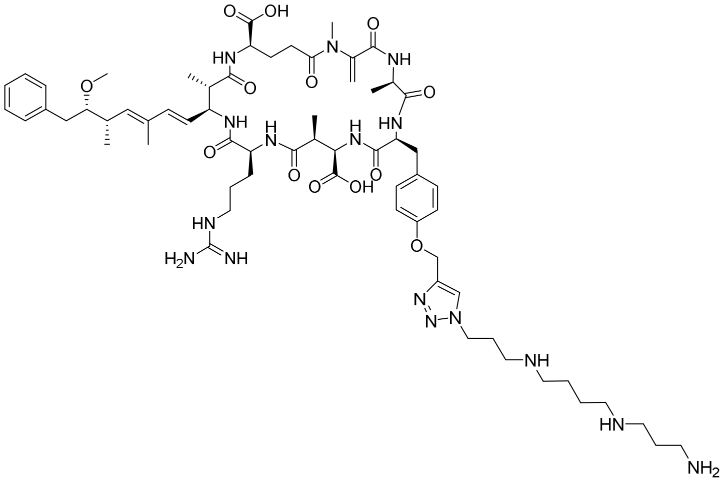

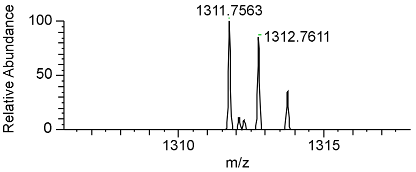

Sum formula

$C_{65}H_{98}N_{16}O_{13}$

pred. *m/z* ([M + H]$^+$)

1311.7572

meas. *m/z* ([M + H]$^+$)

1311.7562(Δ 0.8 ppm)

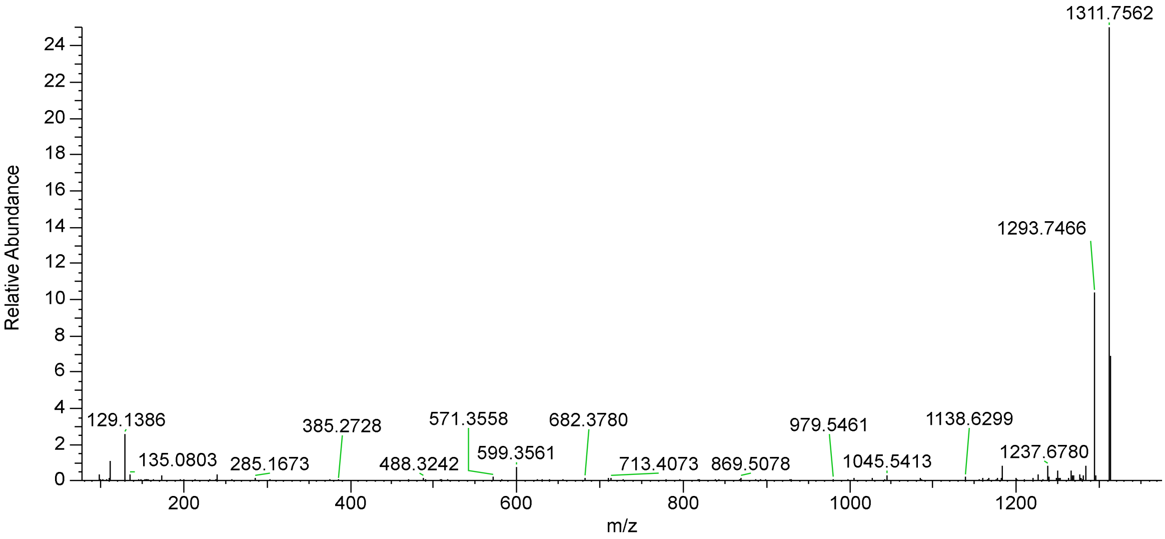

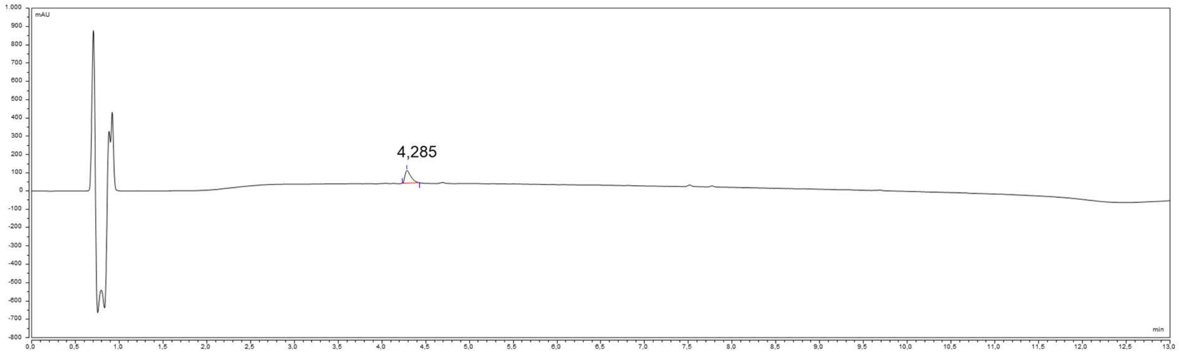

Figure S13. Structure, MS, and MS^2 data of **2j**. Key fragment b5/y5 (Mdha-Ala-PrtyrAzspe-Masp-Arg) *m/z* 869.5078 (0.0026 Da). HPLC-DAD chromatogram at 210 nm.

Microcystin-[Propargyltyrosine | 2-Azidoethyldimethylamine]R (2k)

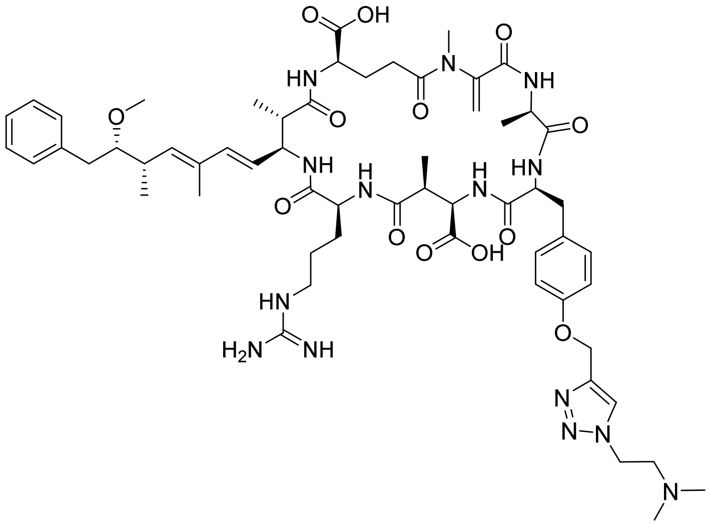

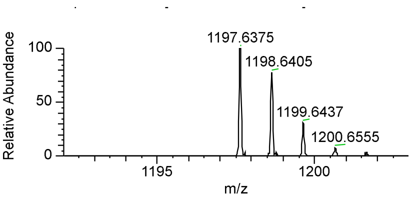

Sum formula

$C_{59}H_{84}N_{14}O_{13}$

pred. ***m/z*** **([M + H]$^+$)**

1197.6415

meas. ***m/z*** **([M + H]$^+$)**

1197.6375 (Δ 3.3 ppm)

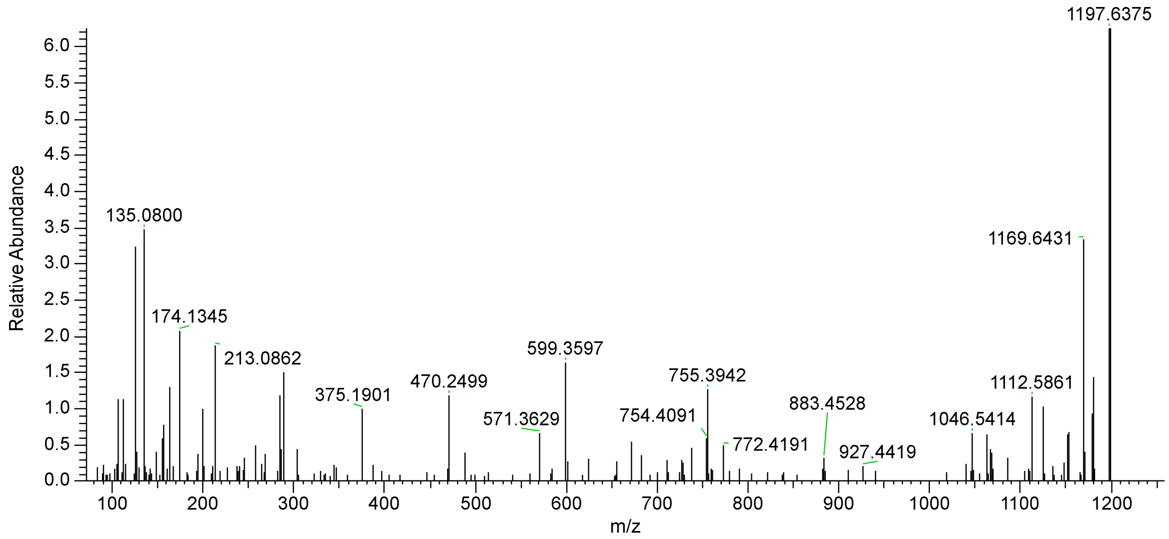

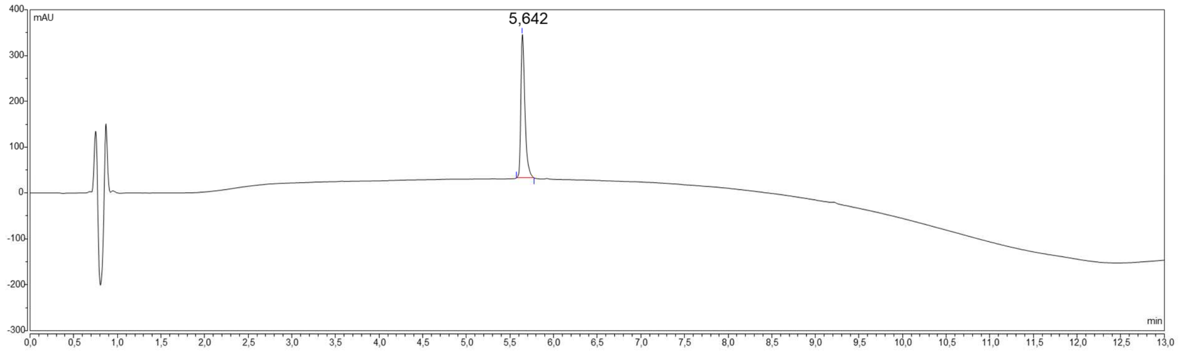

Figure S14. Structure, MS, and MS^2 data of **2k**. Key fragment b5/y5 (Mdha-Ala-PrtyrAzdmam-Masp-Arg) *m/z* 755.3942 (0.0006 Da). HPLC-DAD chromatogram at 210 nm.

Microcystin-[Propargyltyrosine | piperidinoethylazide]R (2l)

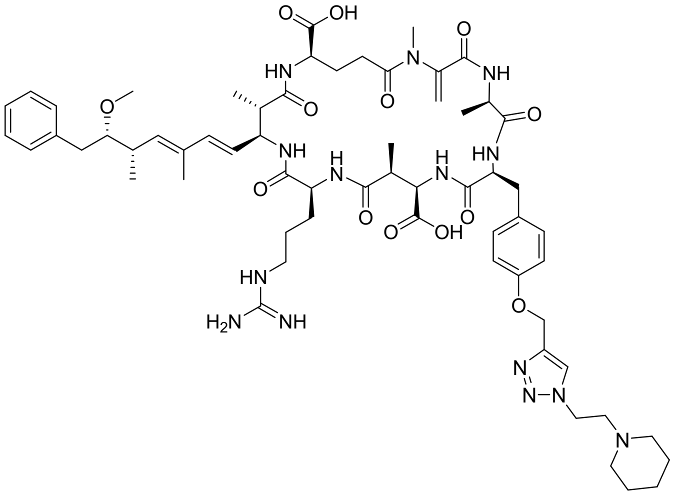

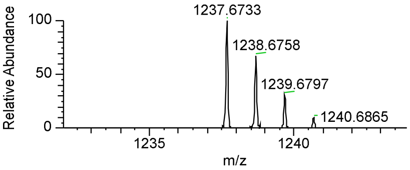

Sum formula

$C_{62}H_{88}N_{14}O_{13}$

pred. *m/z* ([M + H]⁺)

1237.6728

meas. *m/z* ([M + H]⁺)

1237.6733(Δ 0.4 ppm)

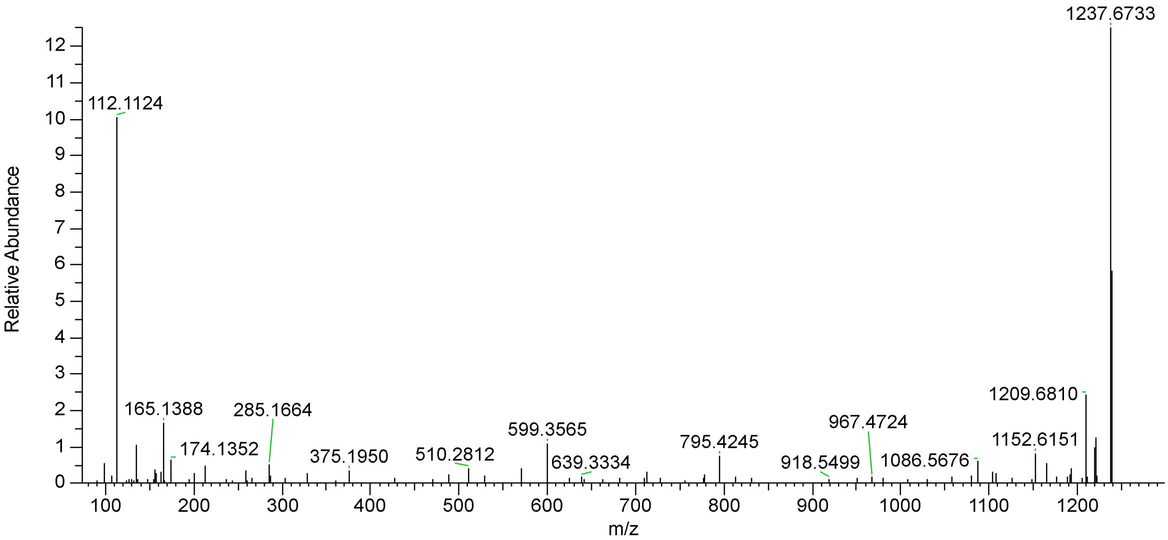

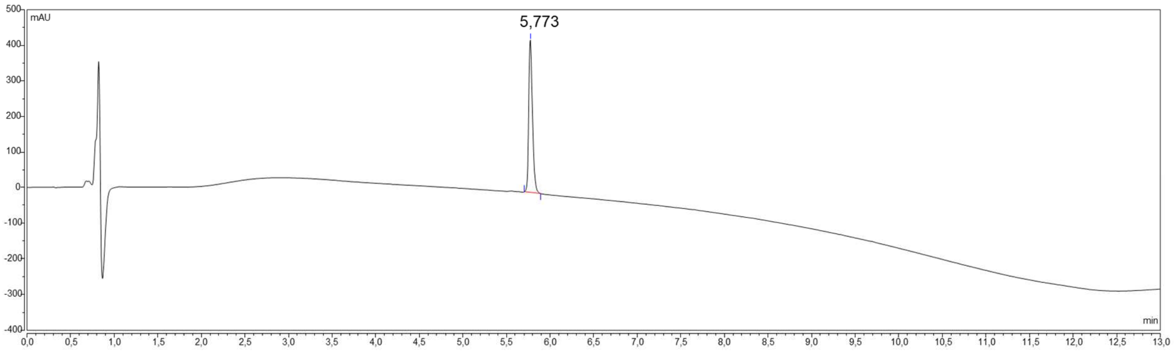

Figure S15. Structure, MS, and MS^2 data of **2l**. Key fragment b5/y5 (Mdha-Ala-PrtyrAzepip-Masp-Arg) *m/z* 795.4245 (0.0015 Da). HPLC-DAD chromatogram at 210 nm.

Microcystin-[Propargyltyrosine | azidoacetic acid]R (2m)

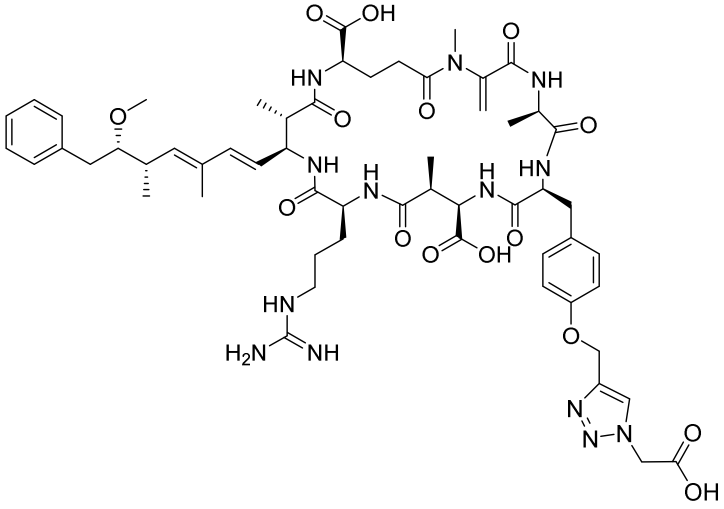

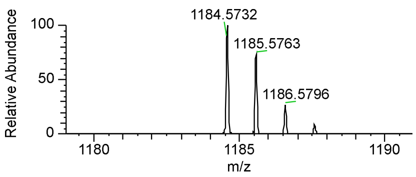

Sum formula

$C_{57}H_{77}N_{13}O_{15}$

pred. ***m/z*** **([M + H]⁺)**

1184.5735

meas. ***m/z*** **([M + H]⁺)**

1184.5732 (Δ 0.3 ppm)

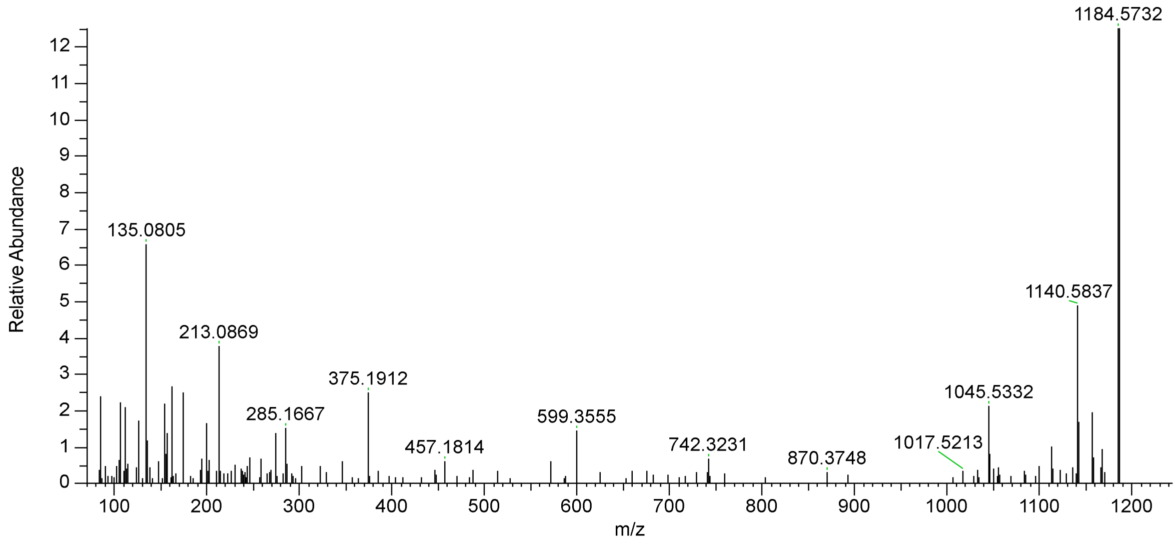

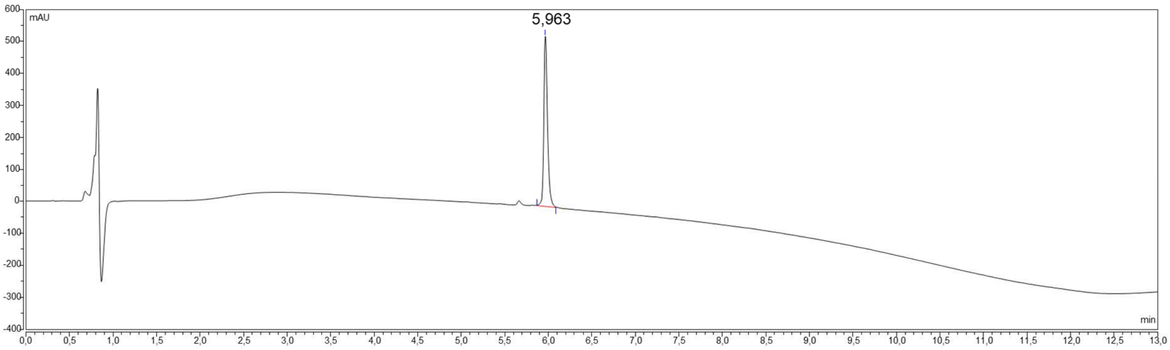

Figure S16. Structure, MS, and MS^2 data of **2m**. Key fragment b5/y5 (Mdha-Ala-PrtyrAzacac-Masp-Arg) *m/z* 742.3231 (0.0036 Da). HPLC-DAD chromatogram at 210 nm.

Microcystin-[Propargyltyrosine | 2-azidopropanoic acid]R (2n)

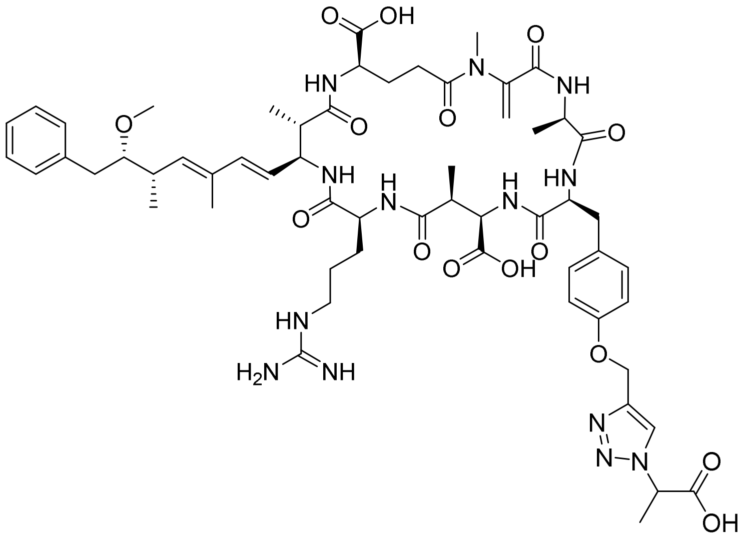

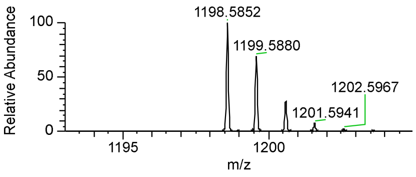

Sum formula

$C_{58}H_{79}N_{13}O_{15}$

pred. *m/z* ([M + H]$^+$)

1198.5891

meas. *m/z* ([M + H]$^+$)

1198.5852 (Δ 3.3 ppm)

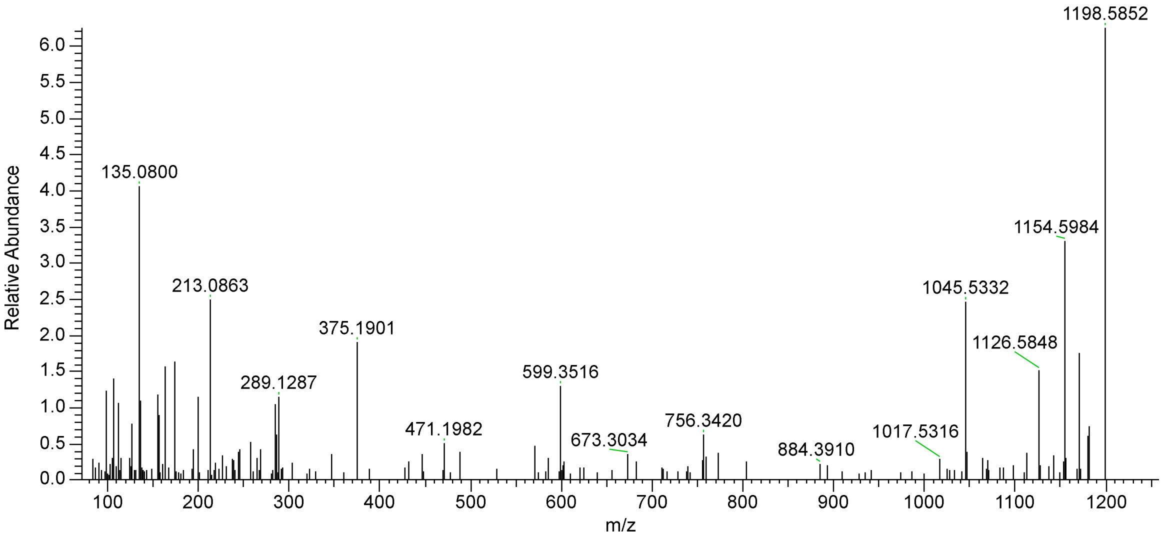

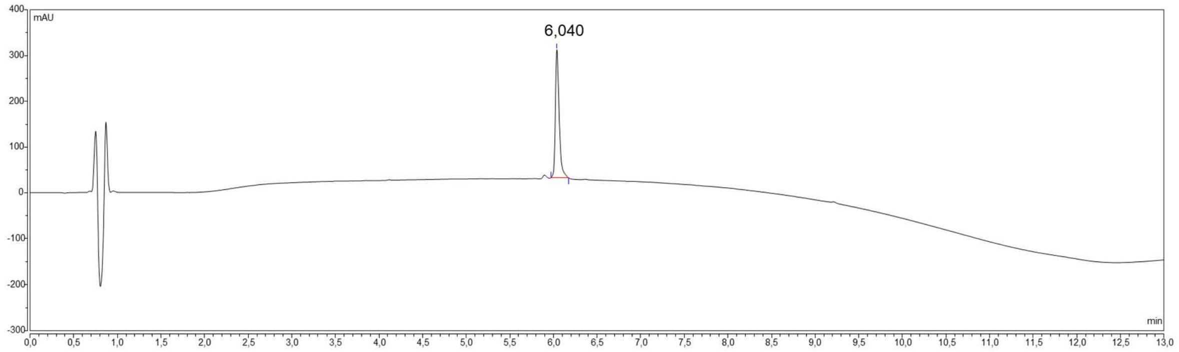

Figure S17. Structure, MS, and MS^2 data of **2n**. Key fragment b5/y5 (Mdha-Ala-PrtyrAzpra-Masp-Arg) *m/z* 756.3420 (0.0004 Da). HPLC-DAD chromatogram at 210 nm.

Microcystin-[Propargyltyrosine|2-azido-2-methylpropanoic acid]R (2o)

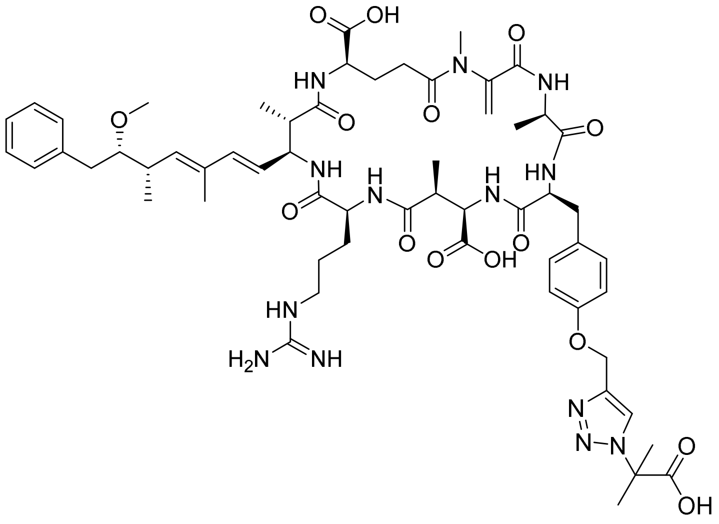

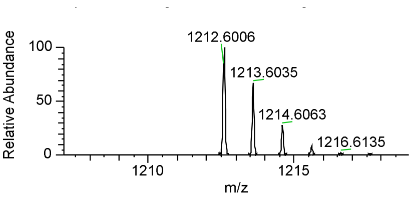

Sum formula

$C_{59}H_{81}N_{13}O_{15}$

pred. ***m/z*** **([M + H]$^+$)**

1212.6048

meas. ***m/z*** **([M + H]$^+$)**

1212.6006 (Δ 3.5 ppm)

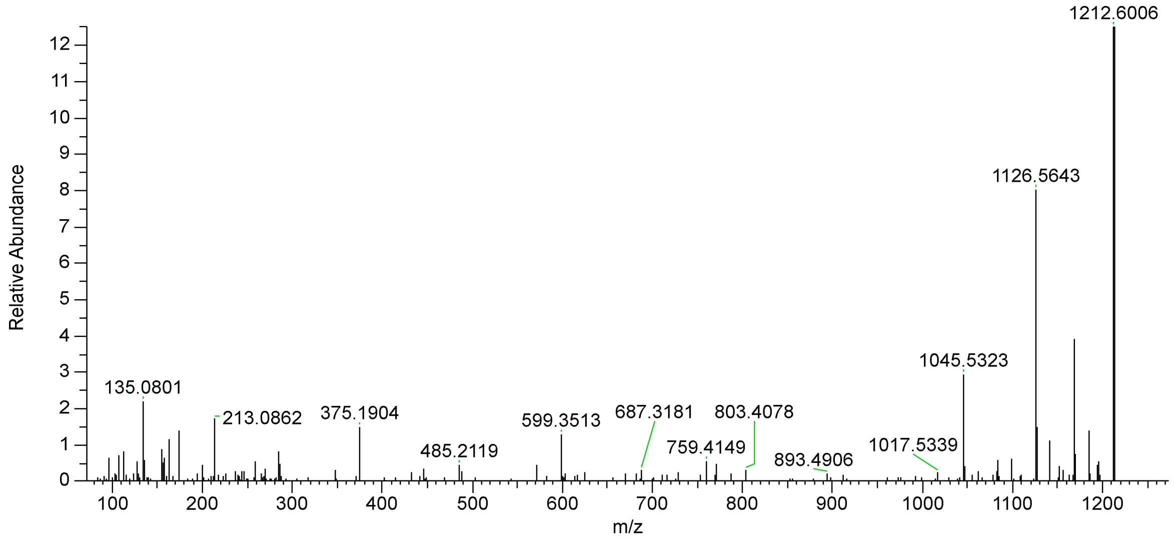

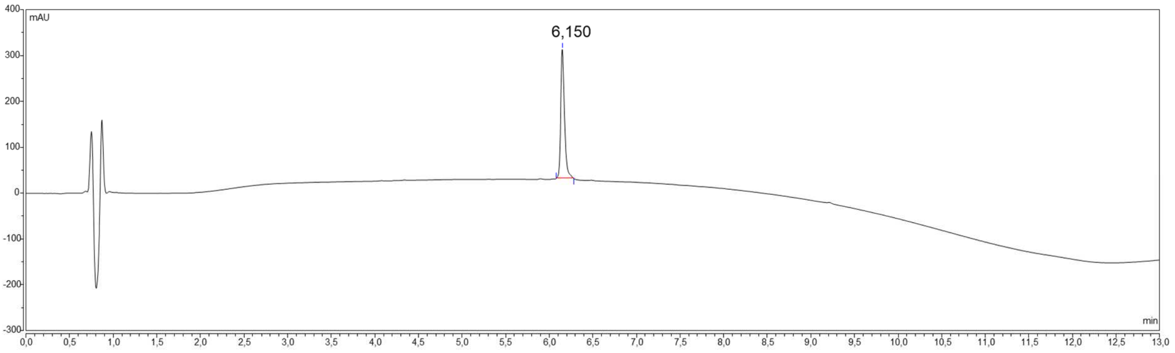

Figure S18. Structure, MS, and MS^2 data of **2o**. Key fragment b5/y5 (Mdha-Ala-PrtyrAzmepra-Masp-Arg) *m/z* 770.3531 (0.0049 Da). HPLC-DAD chromatogram at 210 nm.

Microcystin-[Propargyltyrosine | 3-(4-azido-phenyl)propanoic acid]R (2p)

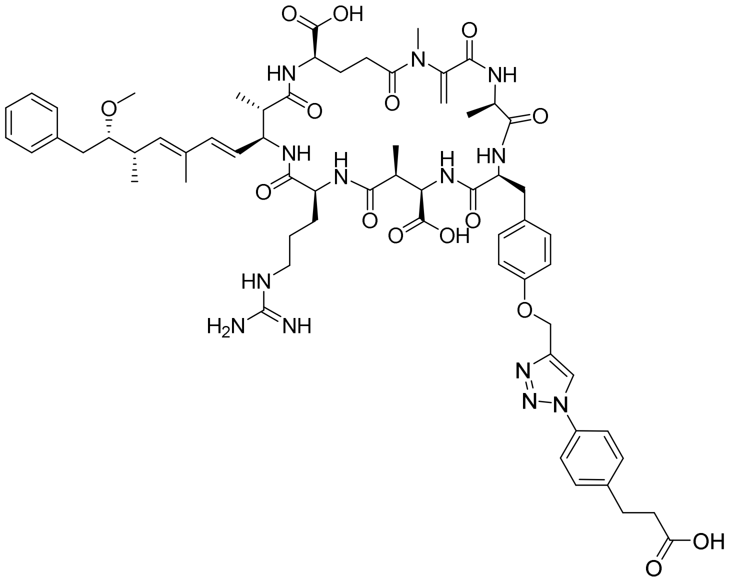

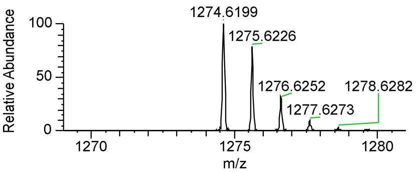

Sum formula

$C_{64}H_{83}N_{13}O_{15}$

pred. ***m/z*** **([M + H]+)**

1274.6204

meas. ***m/z*** **([M + H]+)**

1274.6199(Δ 0.4 ppm)

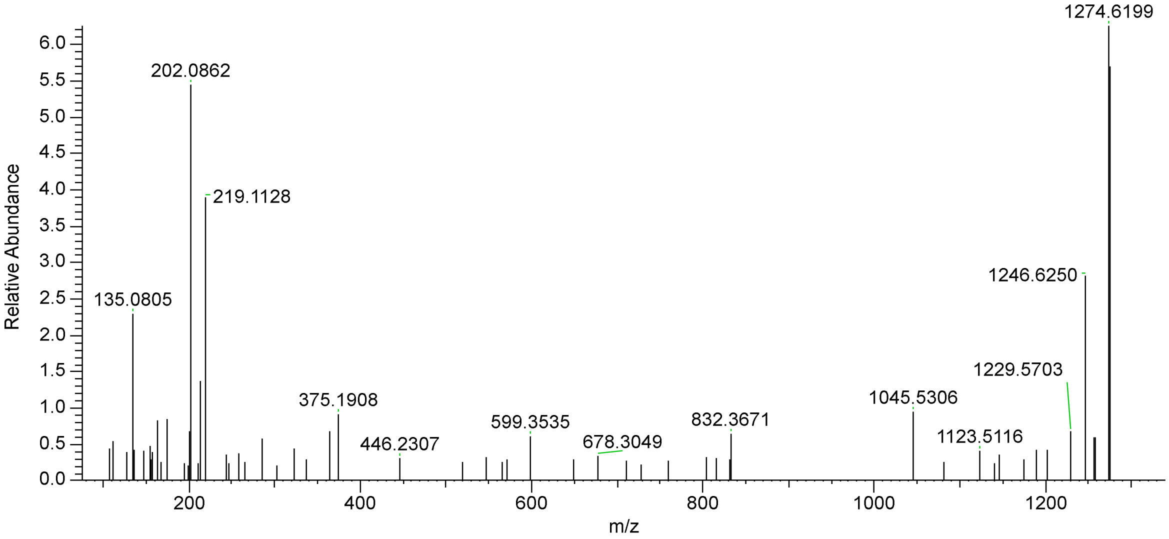

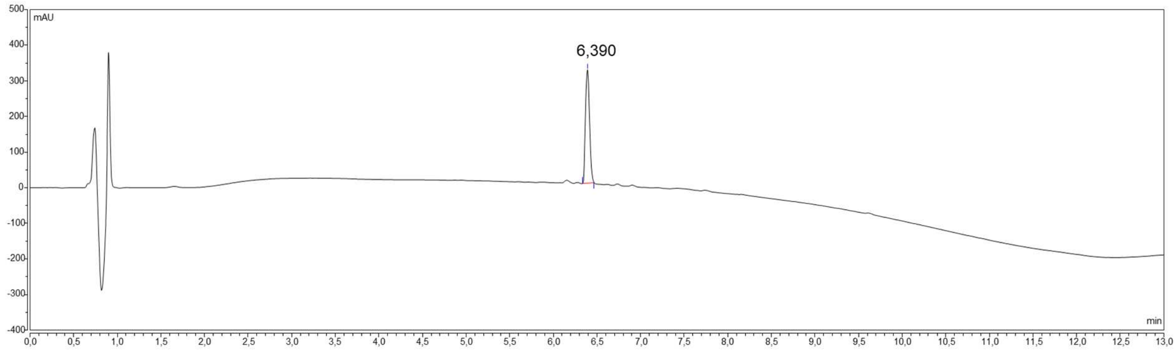

Figure S19. Structure, MS, and MS[2] data of **2p**. Key fragment b5/y5 (Mdha-Ala-PrtyrAzphepra-Masp-Arg) *m/z* 832.3671 (0.0066 Da). HPLC-DAD chromatogram at 210 nm.

Microcystin-[Propargyltyrosine|14-azidomyristic acid]R (2q)

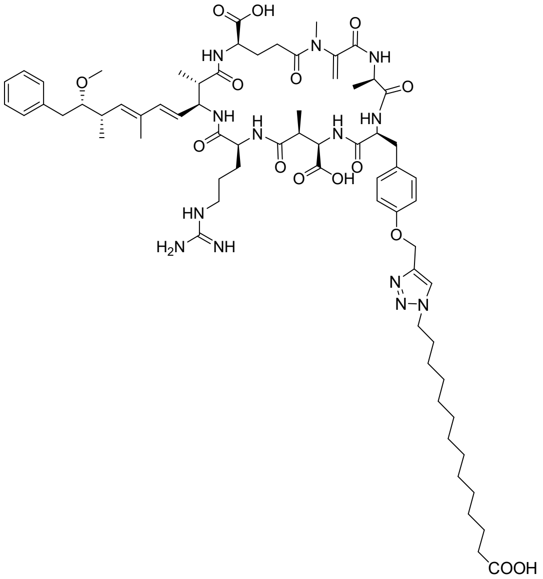

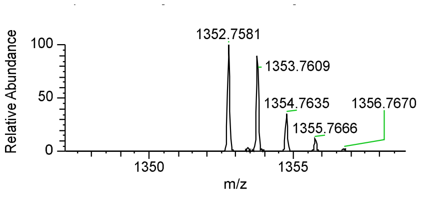

Sum formula

$C_{69}H_{101}N_{13}O_{15}$

pred. *m/z* ([M + H]⁺)

1352.7613

meas. *m/z* ([M + H]⁺)

1352.7581 (Δ 2.4 ppm)

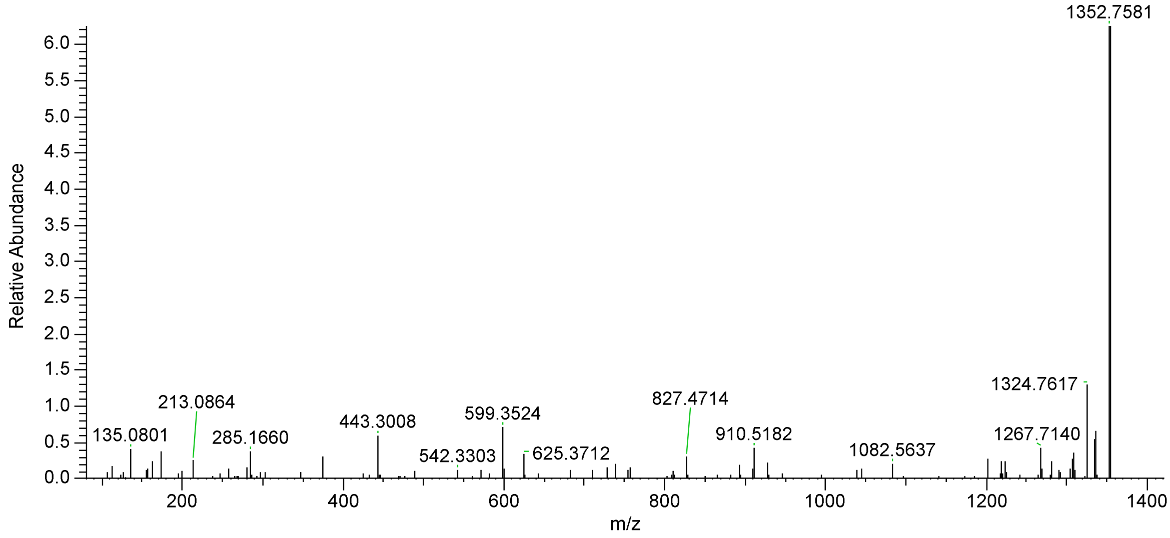

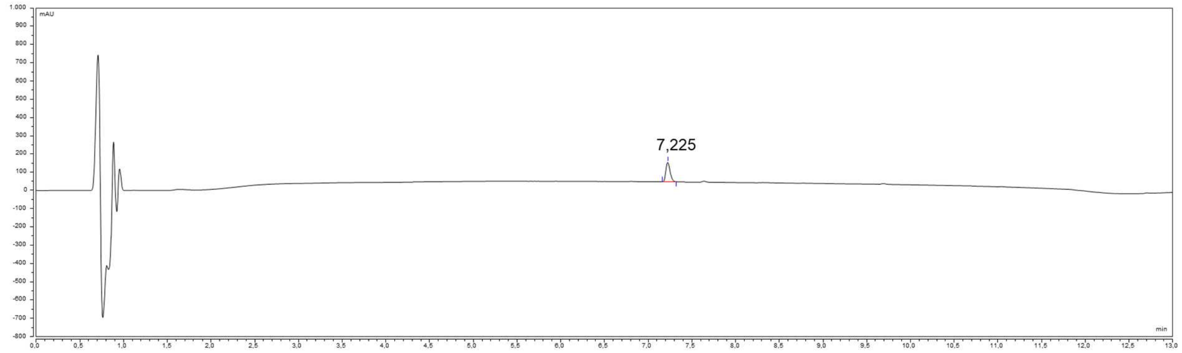

Figure S20. Structure, MS, and MS² data of **2q**. Key fragment b5/y5 (Mdha-Ala-PrtyrAzmya-Masp-Arg) *m/z* 910.5182 (0.0037 Da). HPLC-DAD chromatogram at 210 nm.

Microcystin-[Propargyltyrosine | *N*-(3-azido-propyl)formamide]R (2r)

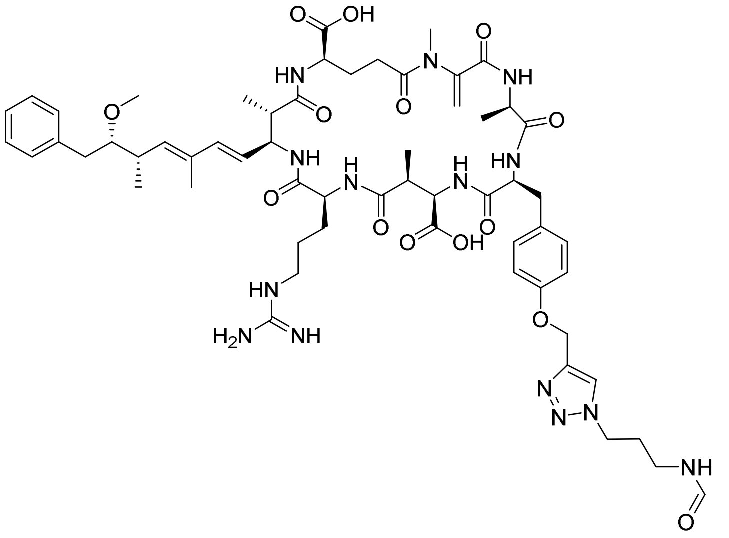

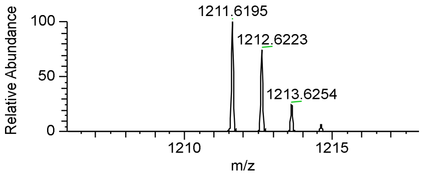

Sum formula

$C_{59}H_{82}N_{14}O_{14}$

pred. *m/z* ([M + H]$^+$)

1211.6208

meas. *m/z* ([M + H]$^+$)

1211.6198(Δ 0.8 ppm)

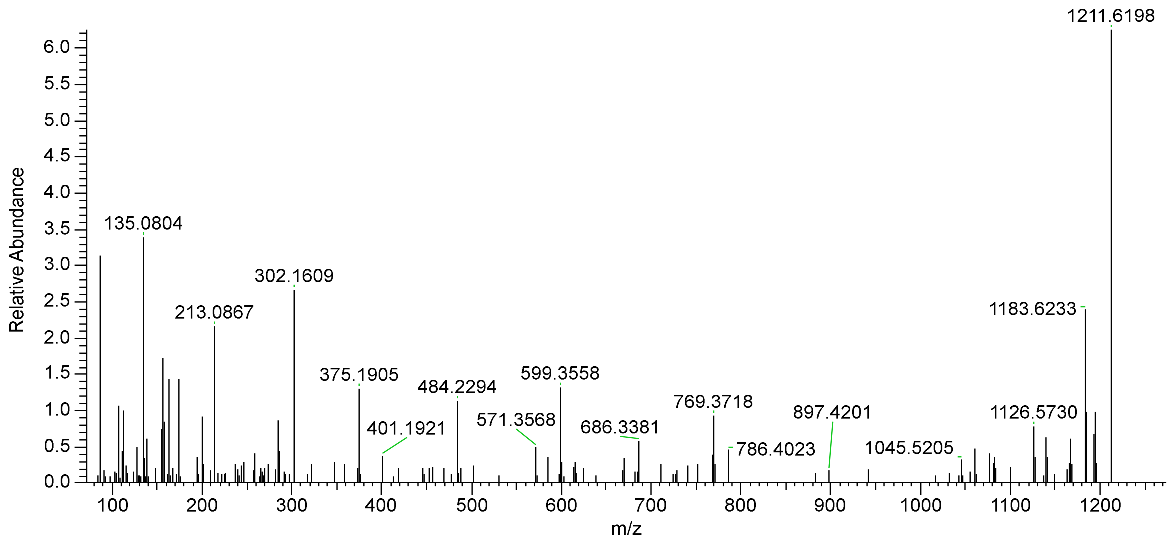

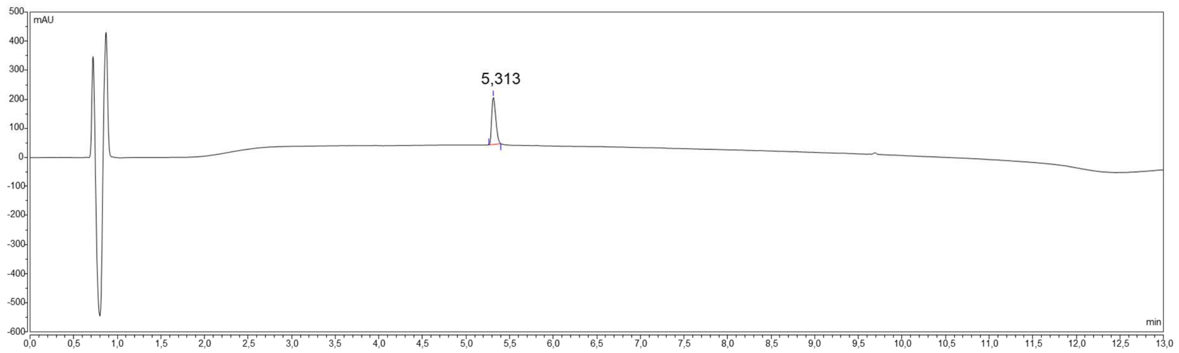

Figure S21. Structure, MS, and MS^2 data of **2r**. Key fragment b5/y5 (Mdha-Ala-PrtyrAzpfam-Masp-Arg) *m/z* 769.3718 (0.0022 Da). HPLC-DAD chromatogram at 210 nm.

Microcystin-[Propargyltyrosine | 2-azidoethanol]R (2s)

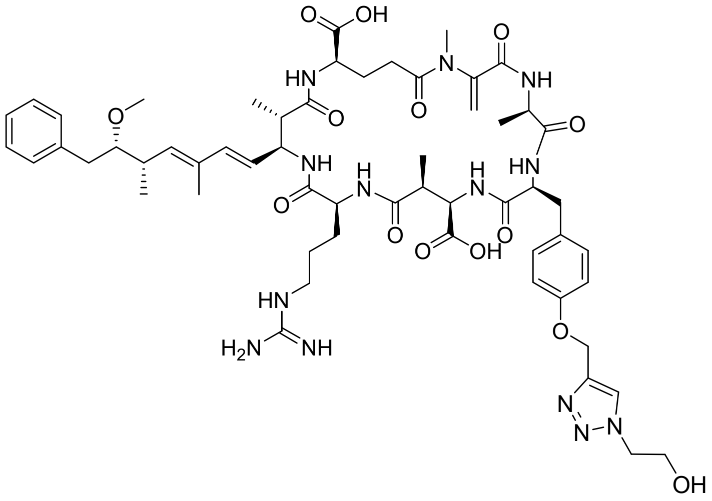

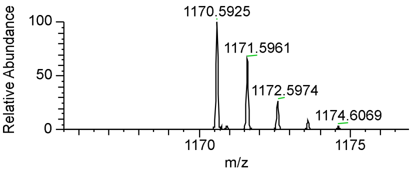

Sum formula

$C_{57}H_{79}N_{13}O_{14}$

pred. *m/z* ([M + H]$^+$)

1170.5942

meas. *m/z* ([M + H]$^+$)

1170.5925 (Δ 1.5 ppm)

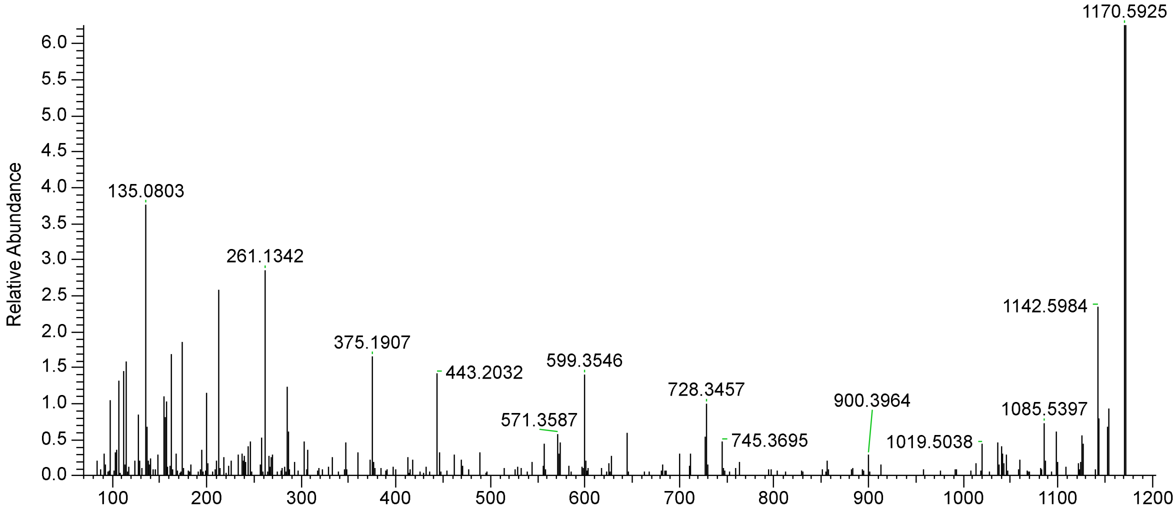

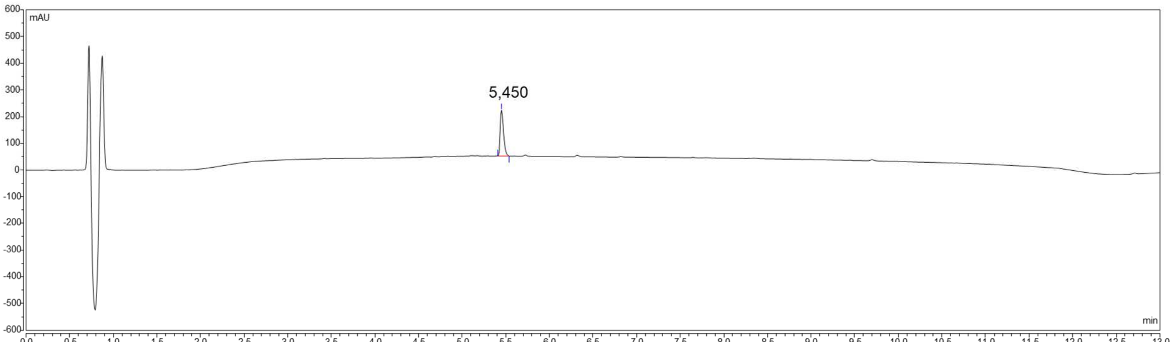

Figure S22. Structure, MS, and MS^2 data of **2s**. Key fragment b5/y5 (Mdha-Ala-PrtyrAzeol-Masp-Arg) *m/z* 728.3457 (0.0017 Da). HPLC-DAD chromatogram at 210 nm.

Microcystin-[Propargyltyrosine | 3-azidopropane-1,2-diol]R (2t)

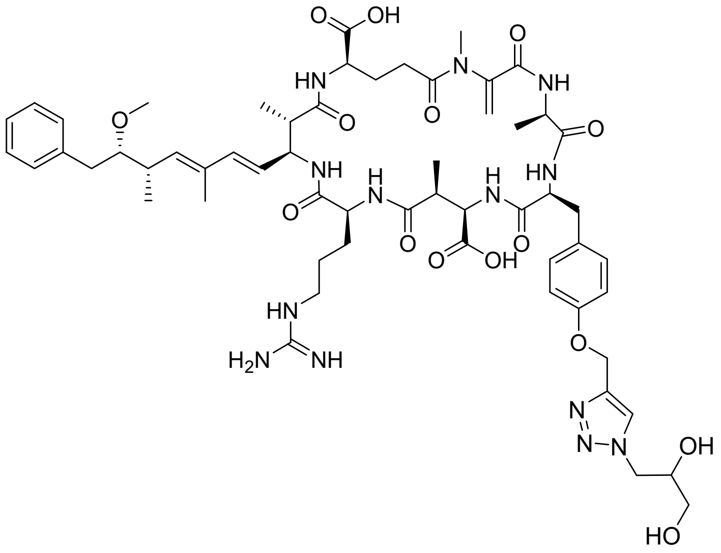

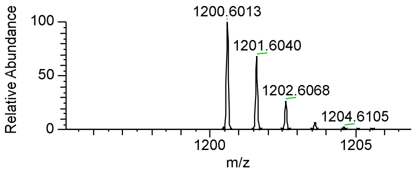

Sum formula

$C_{58}H_{81}N_{13}O_{15}$

pred. ***m/z*** **([M + H]⁺)**

1200.6048

meas. ***m/z*** **([M + H]⁺)**

1200.6013 (Δ 2.9 ppm)

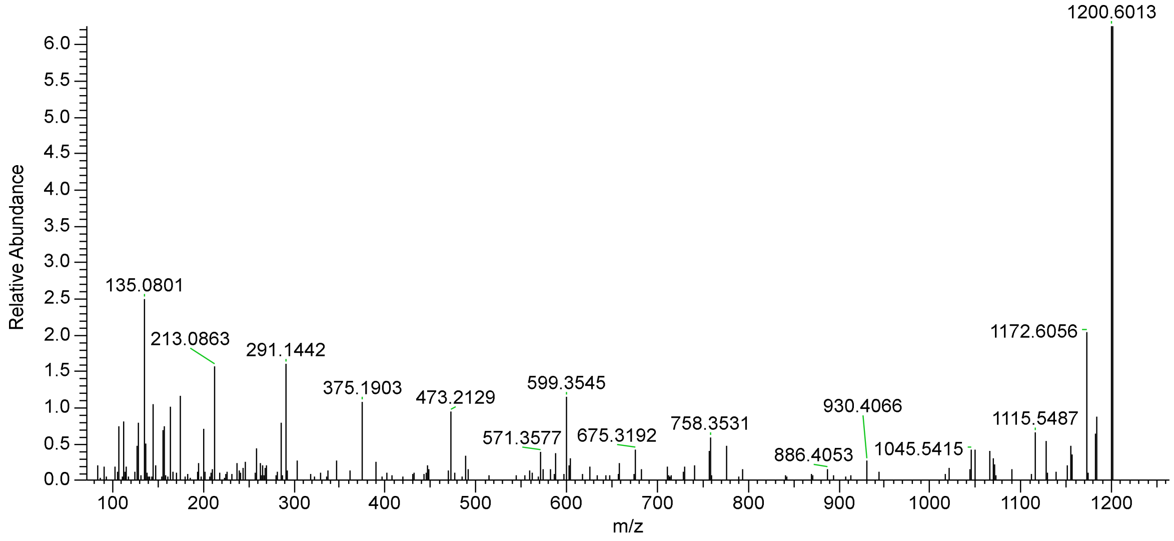

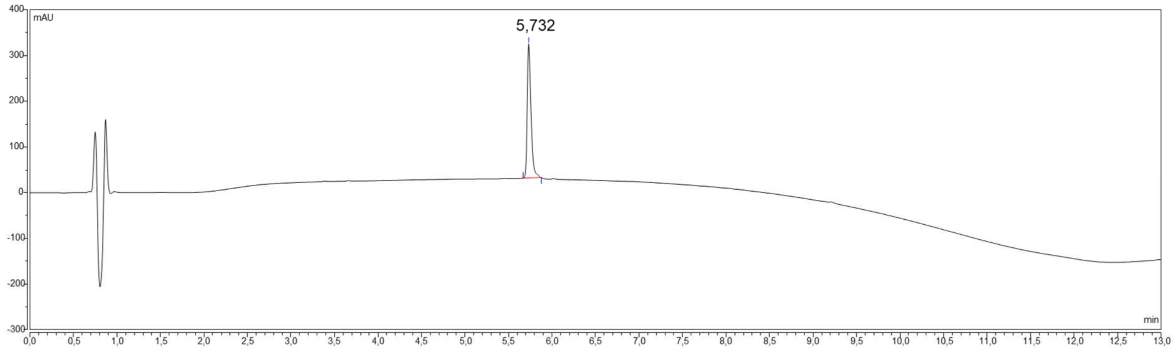

Figure S23. Structure, MS, and MS^2 data of **2t**. Key fragment b5/y5 (Mdha-Ala-PrtyrAzdiol-Masp-Arg) *m/z* 758.3531 (0.0049 Da). HPLC-DAD chromatogram at 210 nm.

Microcystin-[Propargyltyrosine | 1-azido-4-chlorbenzole]R (2u)

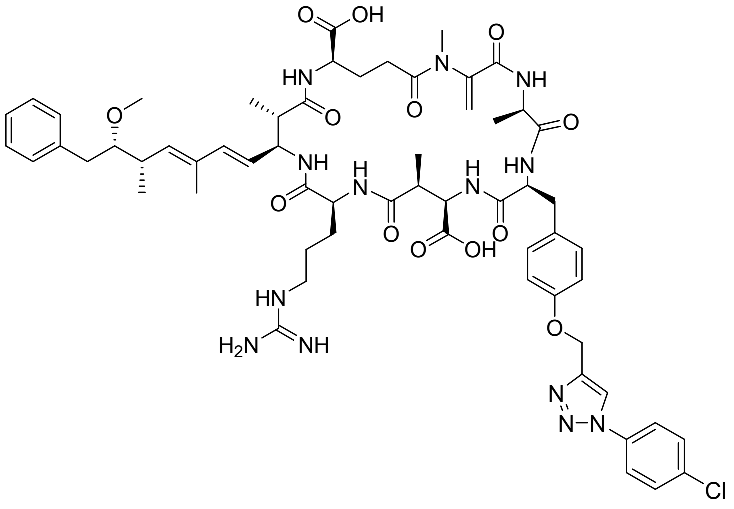

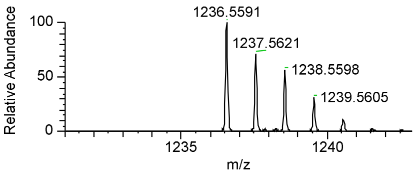

Sum formula

$C_{61}H_{78}ClN_{13}O_{13}$

pred. ***m/z*** **([M + H]+)**

1236.5603

meas. ***m/z*** **([M + H]+)**

1236.5592 (Δ 0.9 ppm)

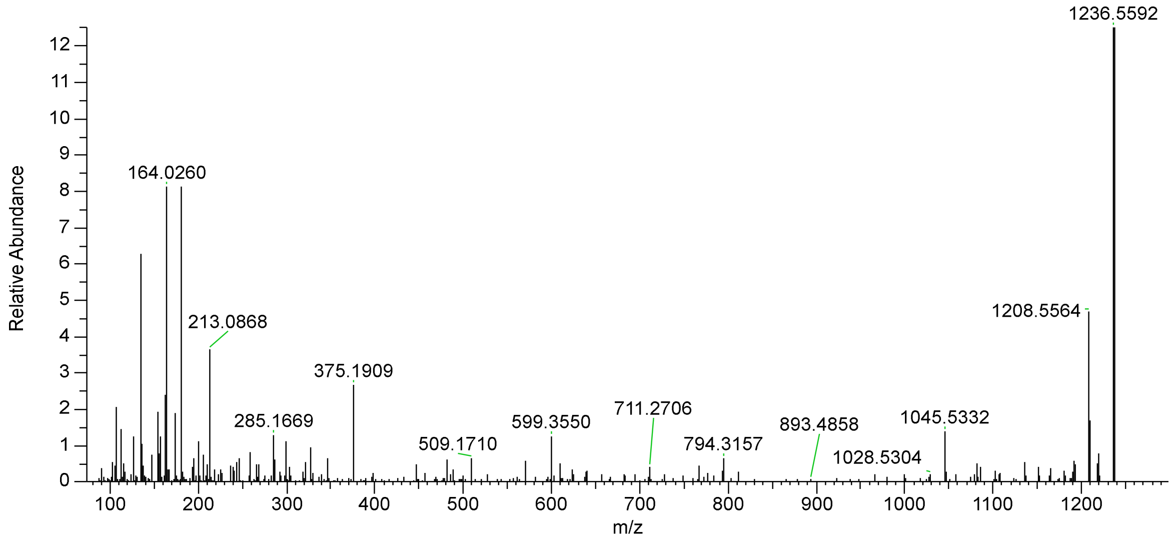

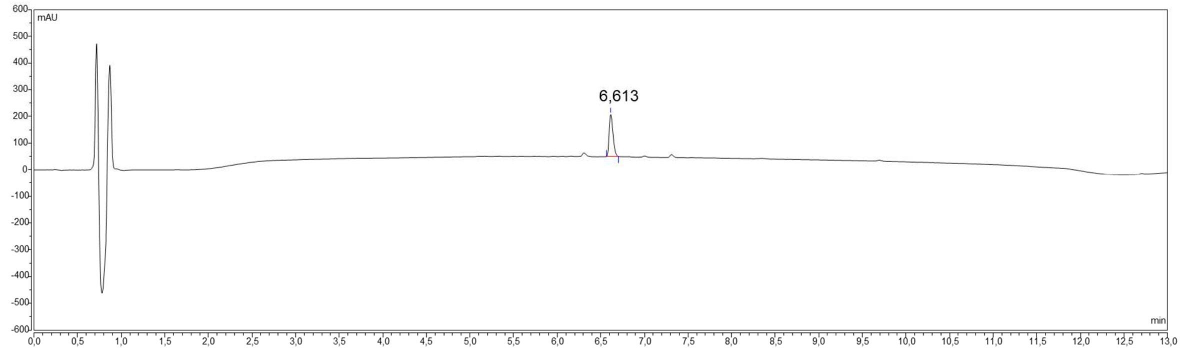

Figure S24. Structure, MS, and MS^2 data of **2u**. Key fragment b5/y5 (Mdha-Ala-PrtyrAzclob-Masp-Arg) *m/z* 794.3157 (0.0021 Da). HPLC-DAD chromatogram at 210 nm.

Microcystin-[Propargyltyrosine | biotin-PEG3-azide]R (2v)

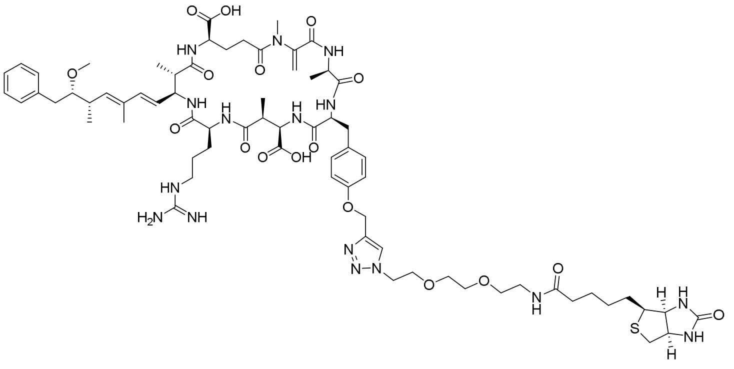

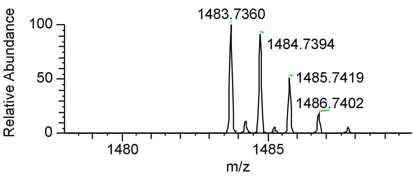

Sum formula

$C_{71}H_{102}N_{16}O_{17}S$

pred. ***m/z*** **([M + H]$^+$)**

1483.7402

meas. ***m/z*** **([M + H]$^+$)**

1483.7360 (Δ 2.8 ppm)

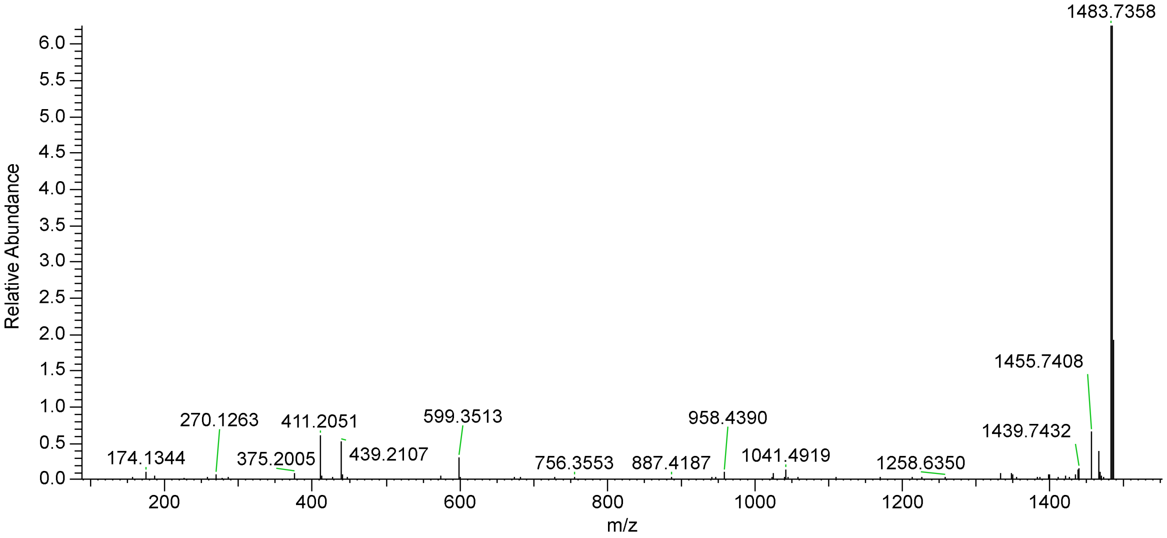

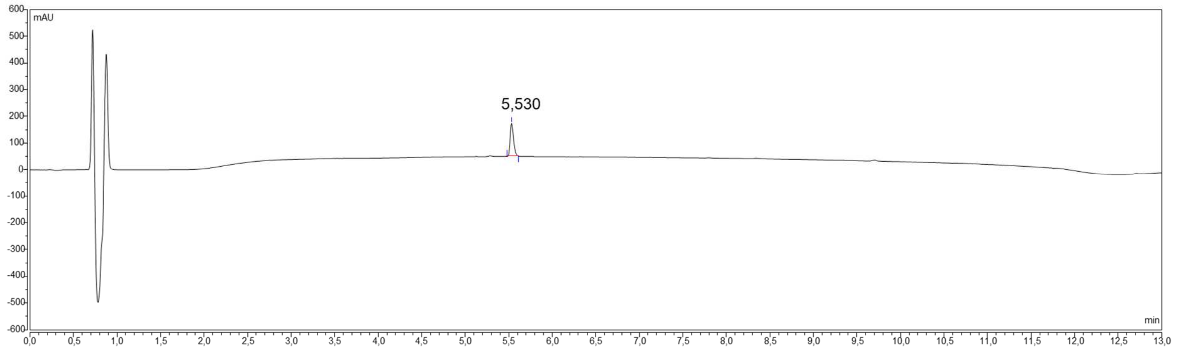

Figure S25. Structure, MS, and MS^2 data of **2v**. Key fragment b5/y5 (Mdha-Ala-PrtyrAzbio-Masp-Arg) *m/z* 1041.4919 (0.0015 Da). HPLC-DAD chromatogram at 210 nm.

Microcystin-[Azidonorvaline]R (3)

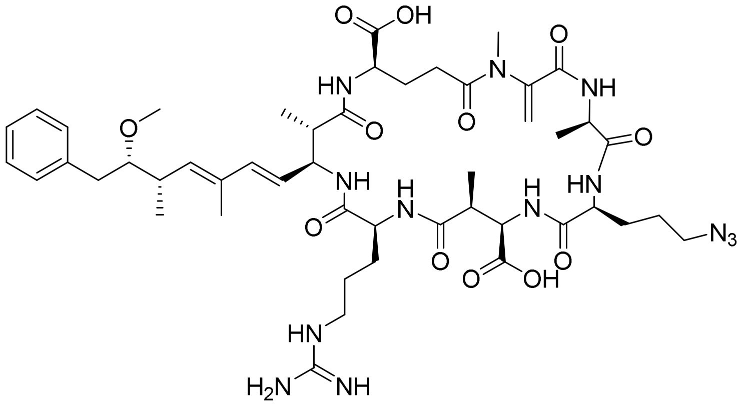

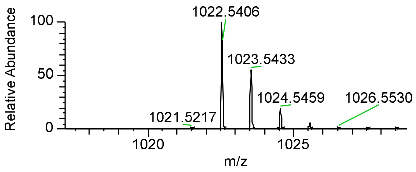

Sum formula

$C_{48}H_{71}N_{13}O_{12}$

pred. ***m/z*** **([M + H]+)**

1022.5418

meas. ***m/z*** **([M + H]+)**

1022.5406 (Δ 1.2 ppm)

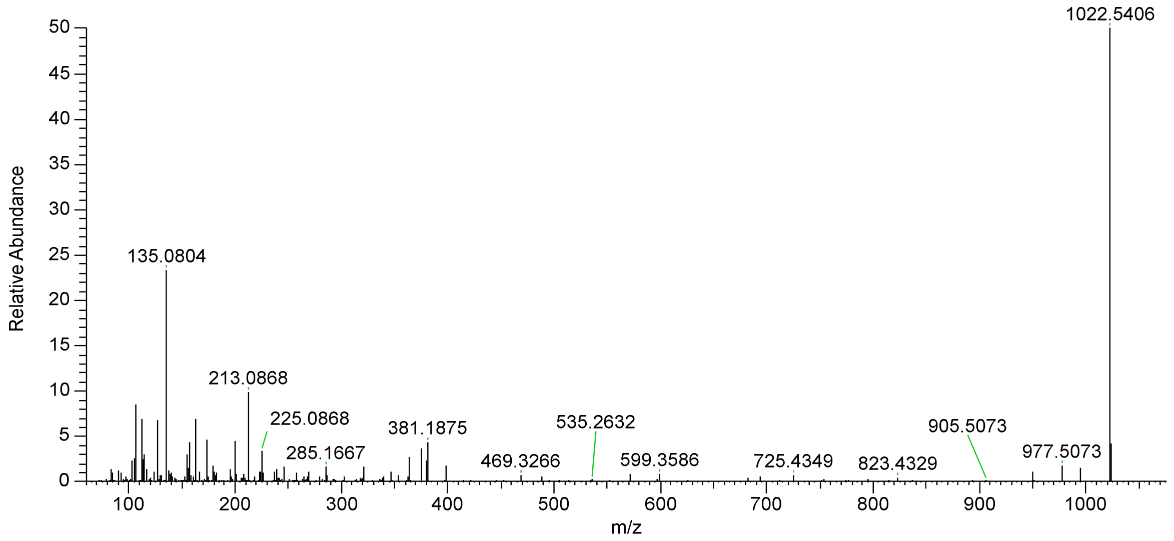

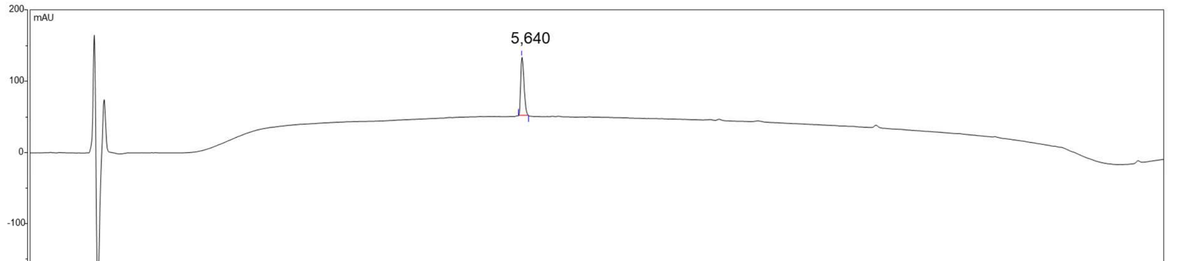

Figure S26. Structure, MS, and MS^2 data of **3**. Key fragment b5/y5 – N_2 (Mdha-Ala-Aznva-Masp-Arg) *m/z* 552.2902 (0.0014 Da). HPLC-DAD chromatogram at 210 nm.

Table S5. Detailed evaluation of the MS/MS spectrum of **3**. Fragments indicated in bold are only explainable if the clickable amino acid is incorporated in the postulated position of the MC core structure.

m/z	**annotation**	**ion**	**difference [Da]**
1022.5406	M + H^+	-	0.0012
994.5359	M + H^+ - N_2	-	0.0003
906.4721	Aznva-Masp-Arg-Adda-Glu-Mdha + H^+ - N_2	z6	0.0001
823.4329	Masp-Arg-Adda-Glu-Mhda-Ala + H^+ - CH_5N_3	b6/y6	0.0003
823.4329	Masp-Arg-Adda-Glu-Mhda-Ala + H^+ - CN_2H_2	z6	0.0003
823.4329	Aznva-Masp-Arg-Adda-Glu + H^+ - N_2	z5	0.0020
753.4294	Arg-Adda-Glu-Mdha-Ala + H^+	b5/y5	0.0021
694.3901	Aznva-Masp-Arg-Adda + H^+ - N_2	z4	0.0022
682.3944	**Arg-Adda-Glu-Mdha + H^+**	b4/y4	0.0021
599.3586	**Arg-Adda-Glu + H^+** or<br>**Masp-Arg-Adda + H^+**	b3/y3	0.0035
552.2844	Mdha-Ala-Aznva-Masp-Arg + H^+ - N_2	b5/y5	0.0045
535.2632	Mdha-Ala-Azna-Masp-Arg + H^+ - N_2	z5	0.0009
398.2140	Aznva-Masp-Arg + H^+ - N_2	b3/y3	0.0006
381.1875	Aznva-Masp-Arg + H^+ - N_2	z3	0.0006
295.1489	**Mdha-Ala-Aznva + H^+**	b3/y3	0.0024
286.1525	Masp-Arg + H^+	b2/y2	0.0015
284.1257	Glu-Mdha-Ala + H^+	b3/y3	0.0016
269.1253	Masp-Arg + H^+ - NH_3	b2/y2	0.0009
266.1161	Glu-Mdha-Ala + H^+ - H_2O	b3/y3	0.0025
244.1298	Masp-Arg + H^+ - CN_2H_2	b2/y2	0.0006
242.1136	Aznva-Masp + H^+ - N_2	b2/y2	0.0001
227.1034	Masp-Arg + H^+ - CH_5N_3	b2/y2	0.0007
227.1034	Masp-Arg + H^+ - CN_2H_2	z2	0.0007
225.0868	Masp-Arg + H^+ - CHN_3H_6	b2/y2	0.0002
225.0868	Aznva-Masp + H^+ - N_2	z2	0.0002
213.0868	Glu-Mdha + H^+	b2/y2	0.0002
196.0602	Glu-Mdha + H^+	z2	0.0003
195.0759	Glu-Mdha + H^+ - H_2O	b2/y2	0.0006
167.0818	**Ala-Aznva + H^+ - N_2**	z2	0.0003
157.1084	Arg + H^+	y1	0.0001
155.0816	Mdha-Ala + H^+	b2/y2	0.0001
140.0822	Arg + H^+ - NH_3	y1	0.0004

140.0822	Arg + H^+	z1	0.0004
138.0546	Mdha-Ala + H^+	z2	0.0003
130.0505	Glu + H^+ or<br>Masp + H^+	y1	0.0007
115.0867	Arg + H^+ - CN_2H_2	y1	0.0001
113.0712	Aznva + H^+ - N_2	y1	0.0003
112.0395	Glu + H^+ - H_2O	y1	0.0002
84.0448	Mdha + H^+	y1	0.0004

Microcystin-[Azidonorvaline | carboxamide-propargylbiotin]R (3w)

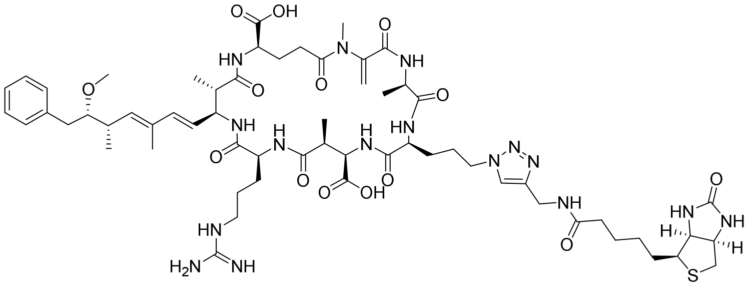

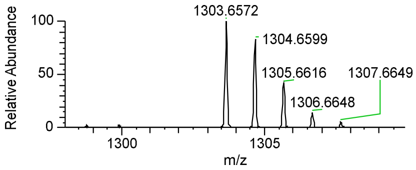

Sum formula

$C_{61}H_{90}N_{16}O_{14}S$

pred. ***m/z*** **([M + H]⁺)**

1303.6616

meas. ***m/z*** **([M + H]⁺)**

1303.6572 (Δ 3.4 ppm)

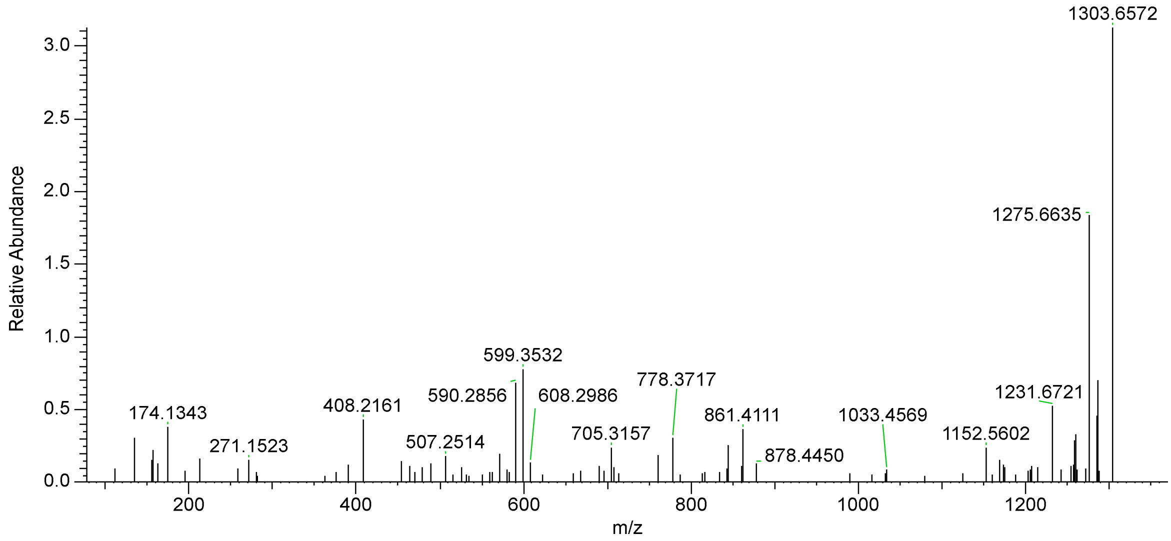

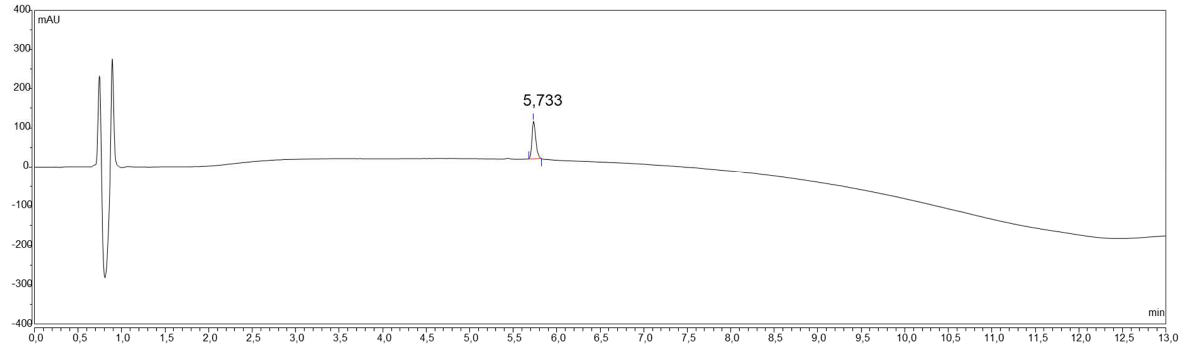

Figure S27. Structure, MS, and MS² data of **3w**. Key fragment b5/y5 (Mdha-Ala-Aznvaprbio-Masp-Arg) *m/z* 861.4111 (0.0037 Da). HPLC-DAD chromatogram at 210 nm.

Microcystin-[Azidonorvaline | 5-chloro-1-pentyne]R (3x)

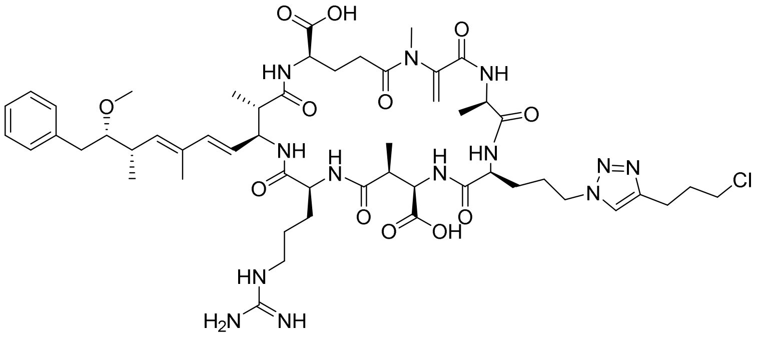

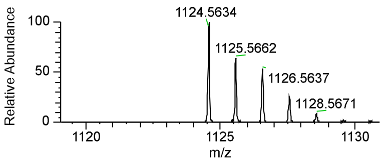

Sum formula

$C_{53}H_{78}ClN_{13}O_{12}$

pred. ***m/z*** **([M + H]$^+$)**

1124.5654

meas. ***m/z*** **([M + H]$^+$)**

1124.5634 (Δ 1.8 ppm)

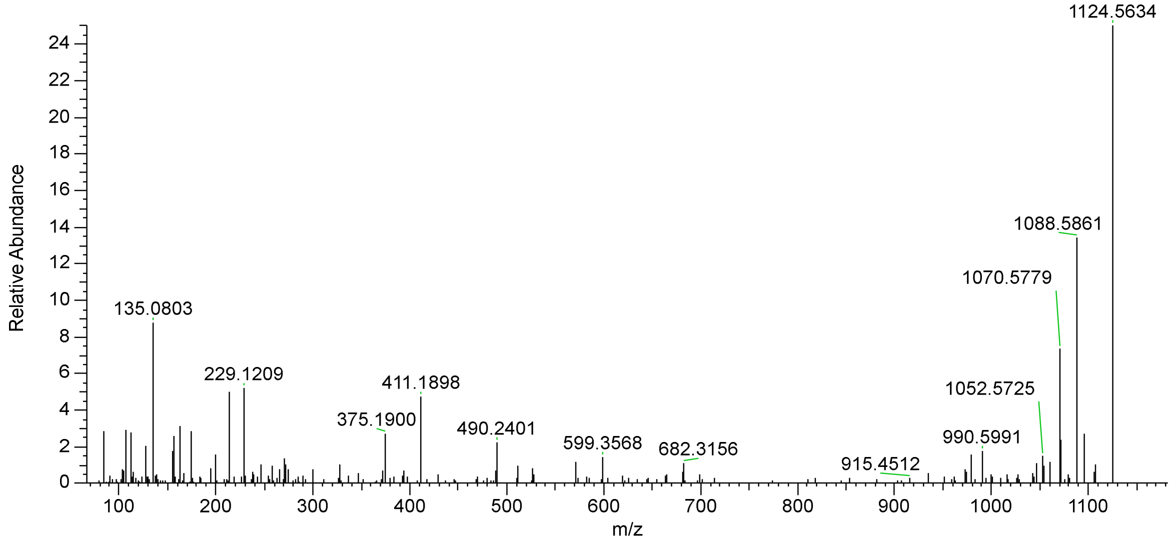

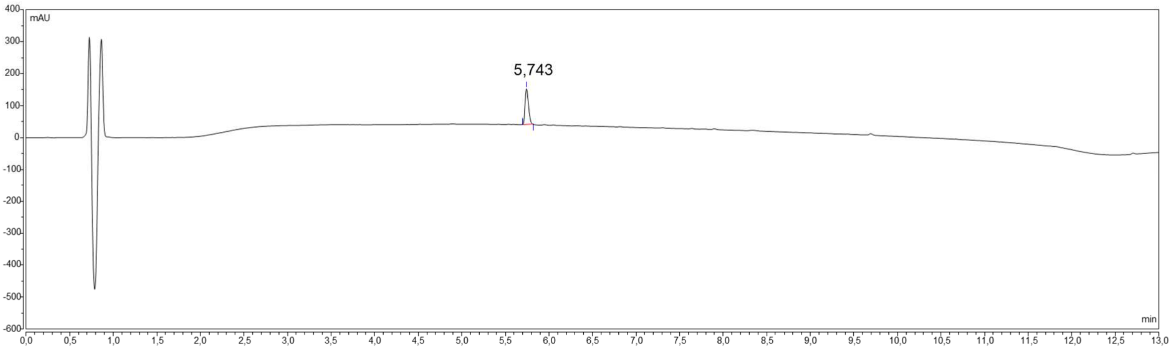

Figure S28. Structure, MS, and MS^2 data of **3x**. Key fragment b5/y5 (Mdha-Ala-Aznvachlopyne-Masp-Arg) *m/z* 682.3156 (0.0030 Da). HPLC-DAD chromatogram at 210 nm.

Microcystin-[Azidonorvaline | propargylamine]R (3y)

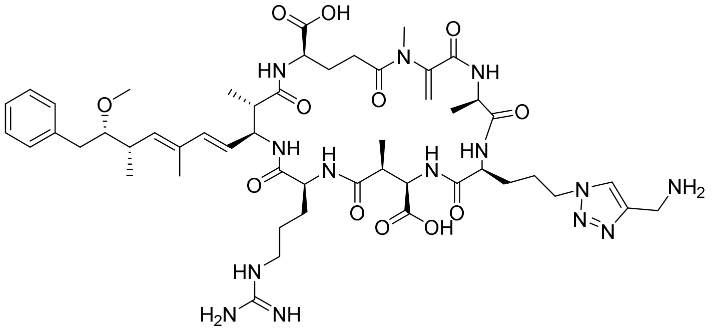

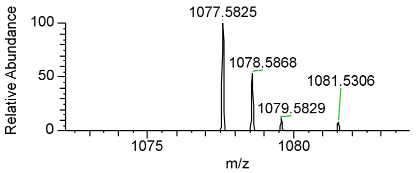

Sum formula
$C_{51}H_{76}N_{14}O_{12}$
pred. *m/z* ([M + H]$^+$)
1077.5840
meas. *m/z* ([M + H]$^+$)
1077.5825 (Δ 1.4 ppm)

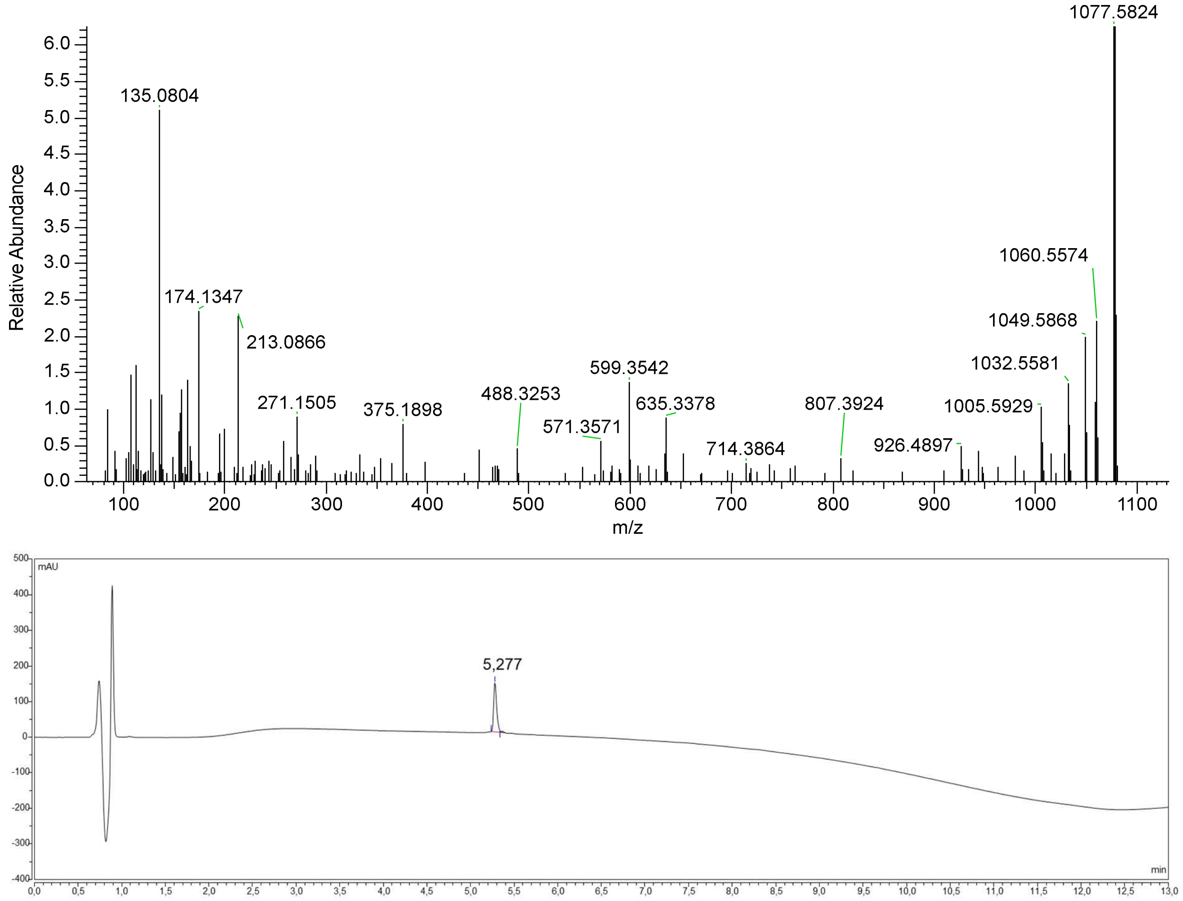

Figure S29. Structure, MS, and MS[2] data of **3y**. Key fragment b5/y5 (Mdha-Ala-Aznvapram-Masp-Arg) *m/z* 635.3378 (0.0006 Da). HPLC-DAD chromatogram at 210 nm.

Microcystin-[Azidonorvaline | propargyllysine]R (3z)

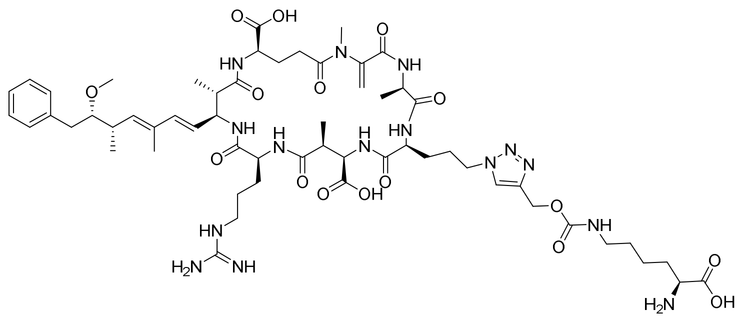

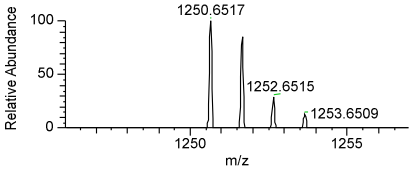

Sum formula

$C_{58}H_{87}N_{15}O_{16}$

pred. ***m/z*** **([M + H]$^+$)**

1250.6528

meas. ***m/z*** **([M + H]$^+$)**

1250.6517 (Δ 0.9 ppm)

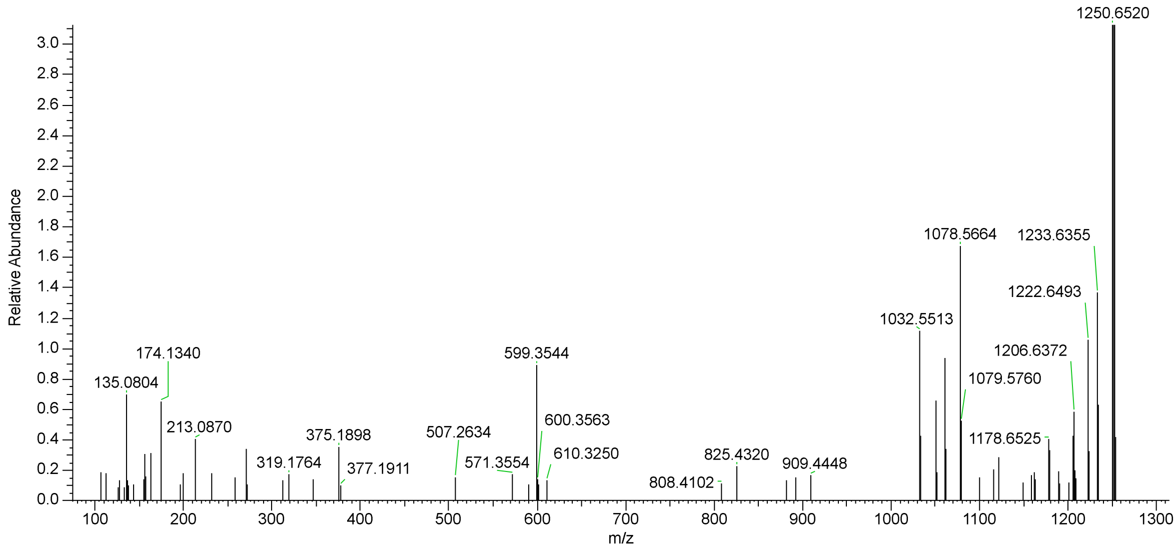

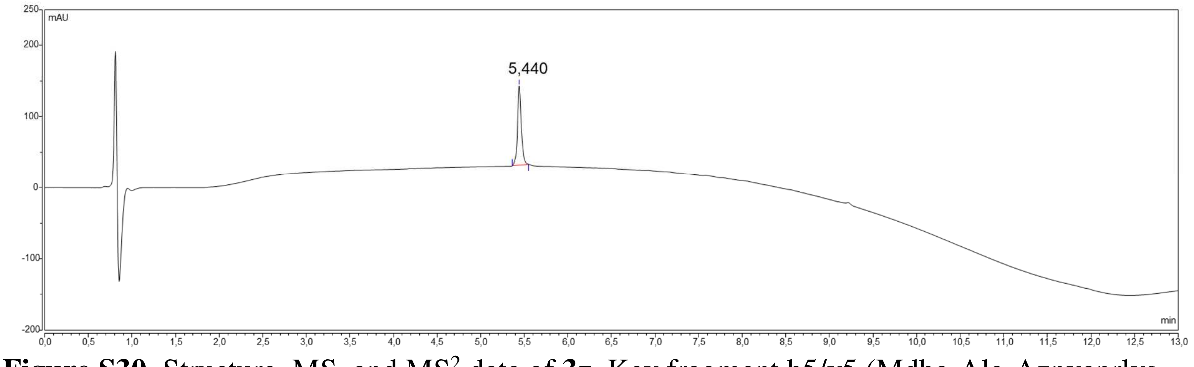

Figure S30. Structure, MS, and MS[2] data of **3z**. Key fragment b5/y5 (Mdha-Ala-Aznvaprlys-Masp-Arg) *m/z* 808.4102 (0.0042 Da). HPLC-DAD chromatogram at 210 nm.

Microcystin-[Azidonorleucine]R (4)

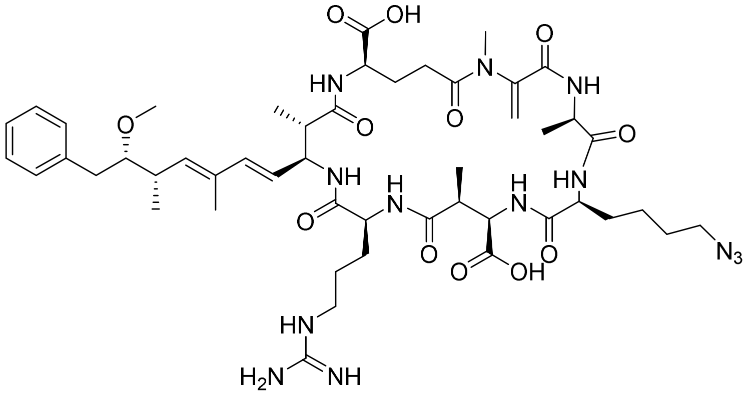

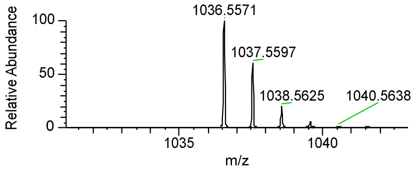

Sum formula

$C_{49}H_{73}N_{13}O_{11}$

pred. *m/z* ([M + H]$^+$)

1036.5574

meas. *m/z* ([M + H]$^+$)

1036.5571 (Δ 0.3 ppm)

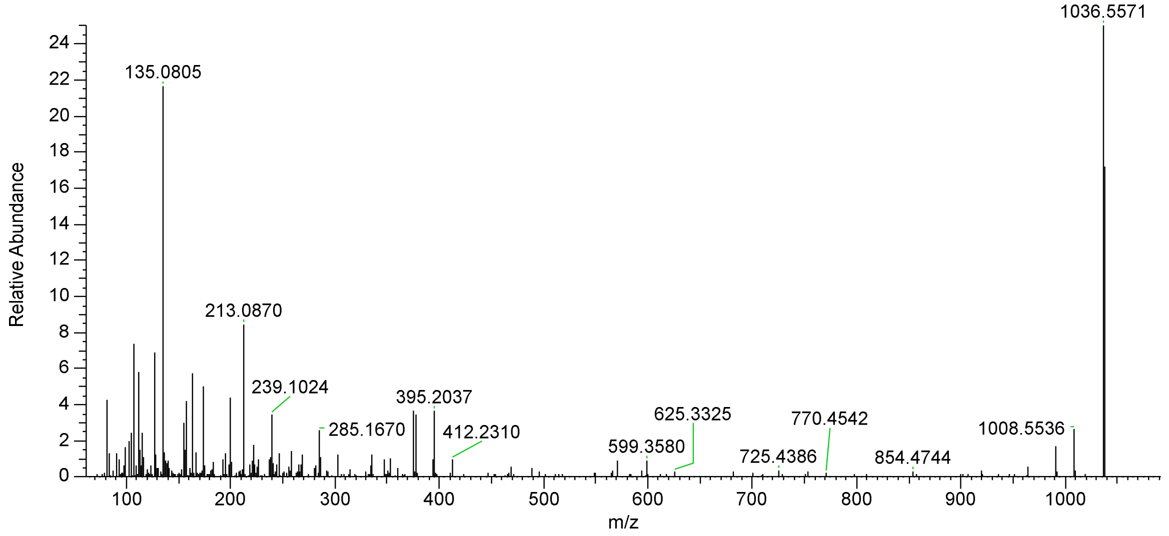

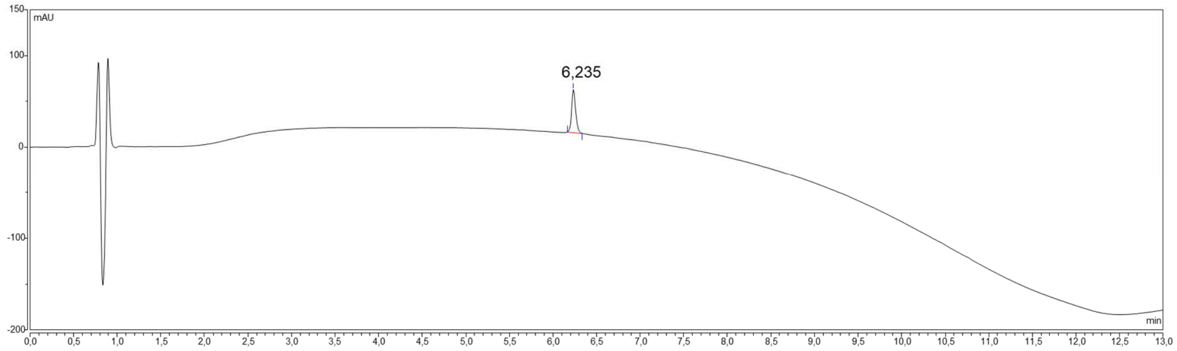

Figure S31. Structure, MS, and MS^2 data of **4**. Key fragment b5/y5 (Mdha-Ala-Aznle-Masp-Arg) *m/z* 594.3151 (0.0045 Da). HPLC-DAD chromatogram at 210 nm.

Table S6. Detailed evaluation of the MS/MS spectrum of **4**. Fragments indicated in bold are only explainable if the clickable amino acid is incorporated in the postulated position of the MC core structure.

m/z	**annotation**	**ion**	**difference [Da]**
1036.5571	M + H^+	-	0.0003
1008.5536	M + H^+ - N_2	-	0.0023
920.4850	Aznle-Masp-Arg-Adda-Glu-Mdha + H^+	z6	0.0026
854.4744	Aznle-Masp-Arg-Adda-Glu + H^+ – N_2	b5/y5	0.0027
753.4315	Arg-Adda-Glu-Mdha-Ala + H^+	b5/y5	0.0021
751.4160	Adda-Glu-Mdha-Ala-Aznle + H^+	b5/y5	0.0022
728.3986	**Masp-Arg-Adda-Glu + H^+**	b4/y4	0.0009
725.4386	Aznle-Masp-Arg-Adda + H^+ – N_2	b4/y4	0.0041
708.4092	Aznle-Masp-Arg-Adda + H^+ – N_2	z4	0.0013
682.3939	**Arg-Adda-Glu-Mdha + H^+**	b4/y4	0.0016
599.3580	**Arg-Adda-Glu + H^+** or<br>**Masp-Arg-Adda + H^+**	b3/y3	0.0028
594.3151	Mdha-Ala-Aznle-Masp-Arg + H^+	b5/y5	0.0045
582.3320	**Arg-Adda-Glu + H^+ - NH_3** or<br>**Masp-Arg-Adda + H^+ - NH_3**	b3/y3	0.0034
582.3320	**Arg-Adda-Glu + H^+** or<br>**Masp-Arg-Adda + H^+**	z3	0.0034
566.3057	Mdha-Ala-Aznle-Masp-Arg + H^+ – N_2	b5/y5	0.0012
549.2773	Mdha-Ala-Aznle-Masp-Arg + H^+ – N_2	z5	0.0007
511.2733	Ala-Aznle-Masp-Arg + H^+	b4/y4	0.0002
466.2450	Ala-Aznle-Masp-Arg + H^+ – N_2	z4	0.0041
453.2879	**Arg-Adda + H^+ - NH_3**	b2/y2	0.0019
453.2879	**Arg-Adda + H^+**	z2	0.0019
423.2098	Aznle-Masp-Arg + H^+ - NH_3	b3/y3	0.0001
423.2098	Aznle-Masp-Arg + H^+	z3	0.0001
412.2310	Aznle-Masp-Arg + H^+ - N_2	b3/y3	0.0007
410.2048	**Mdha-Ala-Aznle-Masp + H^+ - N_2** or<br>**Glu-Mdha-Ala-Aznle + H^+ - N_2**	b4/y4	0.0014
395.2037	Aznle-Masp-Arg + H^+ - N_2	z3	0.0001
309.1641	**Mdha-Ala-Aznle + H^+**	b3/y3	0.0028
297.1838	Adda + H^+	z1	0.0011
286.1501	Masp-Arg + H^+	b2/y2	0.0009
284.1246	Glu-Mdha-Ala + H^+	b3/y3	0.0005
281.1618	**Mdha-Ala-Aznle + H^+ - N_2**	b3/y3	0.0001

269.1256	Masp-Arg + H^+ - NH_3	b2/y2	0.0012
269.1256	Masp-Arg + H^+	z2	0.0012
266.1154	Glu-Mdha-Ala + H^+- H_2O	b3/y3	0.0018
264.1349	**Mdha-Ala-Aznle + H^+ - N_2**	z3	0.0007
256.1288	Aznle-Masp + H^+ - N_2	b2/y2	0.0004
244.1288	Masp-Arg + H^+ - CN_2H_2	b2/y2	0.0004
239.1024	Aznle-Masp + H^+ - N_2	z2	0.0002
227.1029	Masp-Arg + H^+ - CH_5N_3	b2/y2	0.0002
227.1029	Masp-Arg + H^+ - CN_2H_2	z2	0.0002
213.0870	Glu-Mdha + H^+	b2/y2	0.0001
198.1234	**Ala-Aznle + H^+ - N_2**	b2/y2	0.0003
196.0608	Glu-Mdha + H^+	z2	0.0004
195.0763	Glu-Mdha + H^+-H_2O	b2/y2	0.0001
181.0983	**Ala-Aznle + H^+ - N_2**	z2	0.0011
157.1085	Arg + H^+	y1	0.0001
155.0816	Mdha-Ala + H^+	b2/y2	0.0001
140.0817	Arg + H^+	z1	0.0002
140.0817	Arg + H^+ - NH_3	y1	0.0002
138.0550	Mdha-Ala + H^+	z2	0.0001
130.0498	Glu + H^+ or<br>Masp + H^+	y1	0.0001
127.0867	Aznle + H^+ - N_2	y1	0.0001
115.0869	Arg + H^+ - CN_2H_2	y1	0.0003
112.0394	Glu + H^+-H_2O	y1	0.0001
110.0604	Aznle + H^+ - N_2	z1	0.0004
98.0604	Arg + H^+ - CH_5N_3	y1	0.0004
98.0604	Arg + H^+ - CN_2H_2	z1	0.0004
84.0449	Mdha + H^+	y1	0.0005

Microcystin-[Azidonorleucine | carboxamide-propargylbiotin]R (4w)

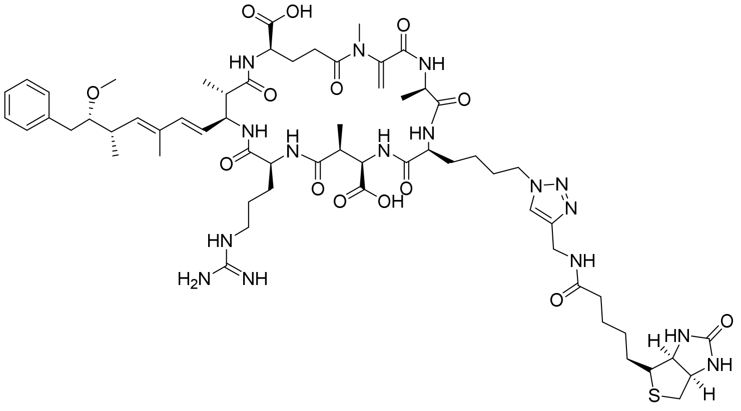

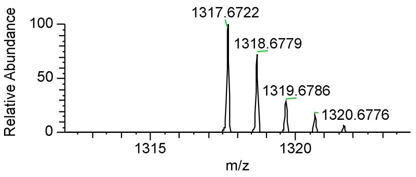

Sum formula

$C_{62}H_{92}N_{16}O_{14}S$

pred. ***m/z*** **([M + H]$^+$)**

1317.6772

meas. ***m/z*** **([M + H]$^+$)**

1317.6722 (Δ 3.8 ppm)

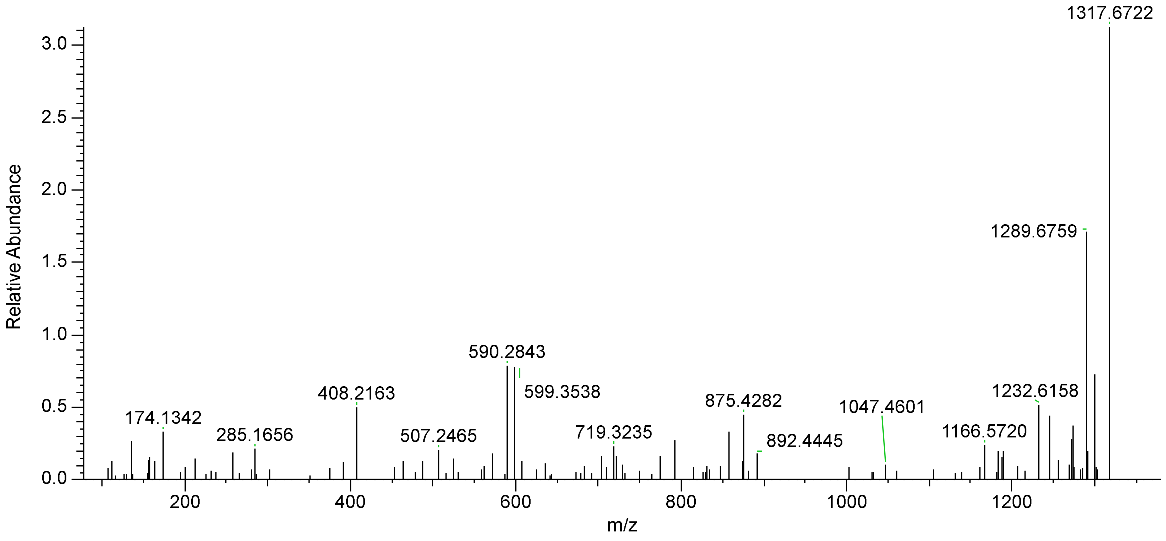

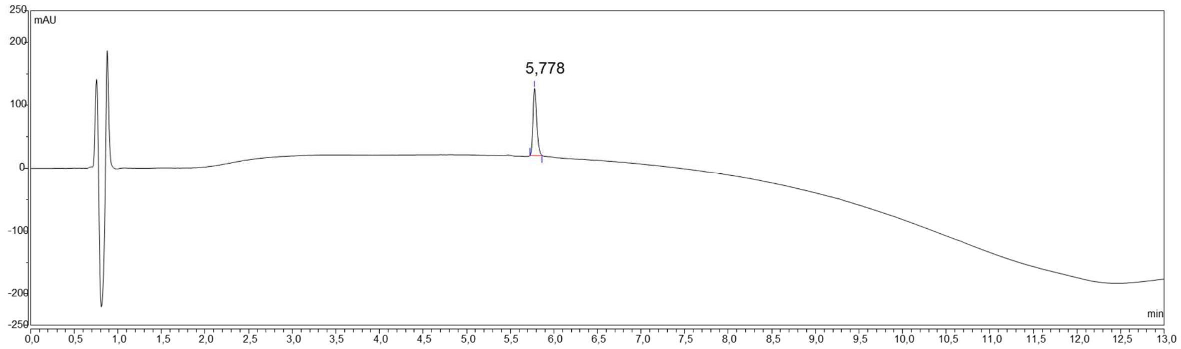

Figure S32. Structure, MS, and MS^2 data of **4w**. Key fragment b5/y5 (Mdha-Ala-AznlePrbio-Masp-Arg) *m/z* 875.4282 (0.0022 Da). HPLC-DAD chromatogram at 210 nm.

Microcystin-[Azidonorleucine | chlorpentyne]R (4x)

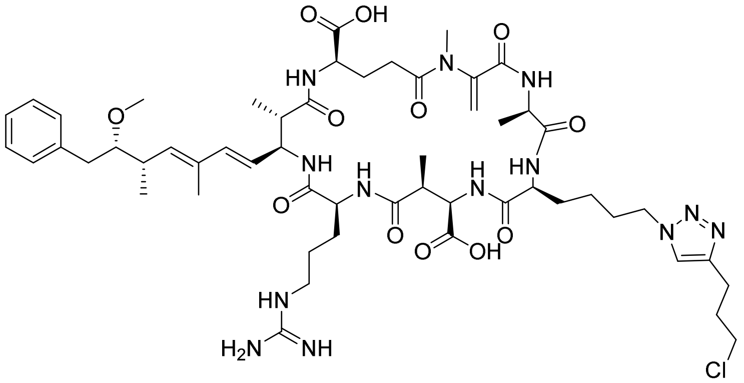

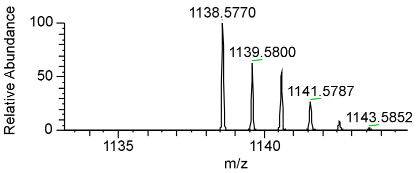

Sum formula

$C_{54}H_{80}N_{13}O_{12}Cl$

pred. ***m/z*** **([M + H]⁺)**

1138.5811

meas. ***m/z*** **([M + H]⁺)**

1138.5770 (Δ 3.6 ppm)

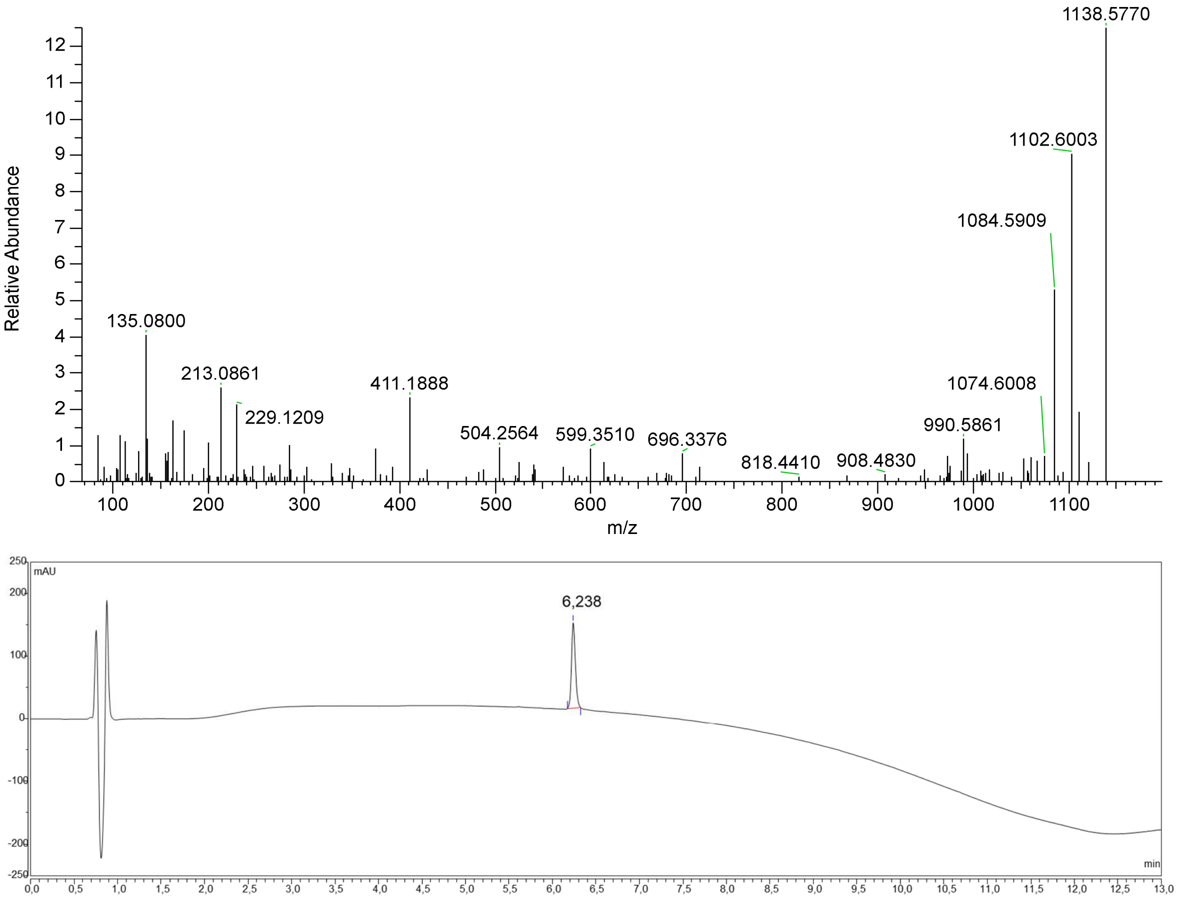

Figure S33. Structure, MS, and MS^2 data of **4x**. Key fragment b5/y5 (Mdha-Ala-AznleChlopyne-Masp-Arg) *m/z* 696.3376 (0.0033 Da). HPLC-DAD chromatogram at 210 nm.

Microcystin-[Azidonorleucine | propargylamine]R (4y)

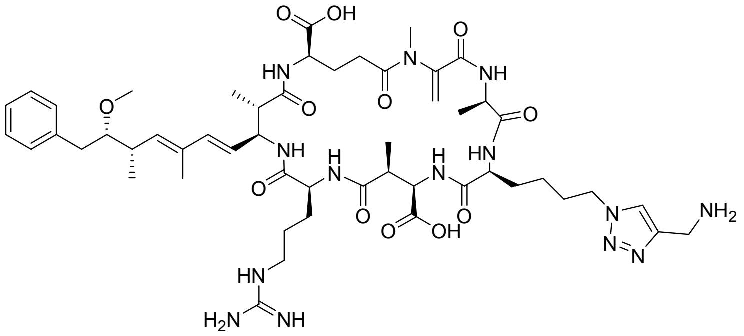

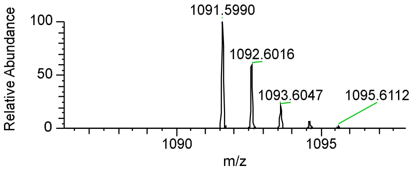

Sum formula

$C_{52}H_{78}N_{14}O_{12}$

pred. ***m/z*** **([M + H]+)**

1091.5996

meas. ***m/z*** **([M + H]+)**

1091.5990 (Δ 0.5 ppm)

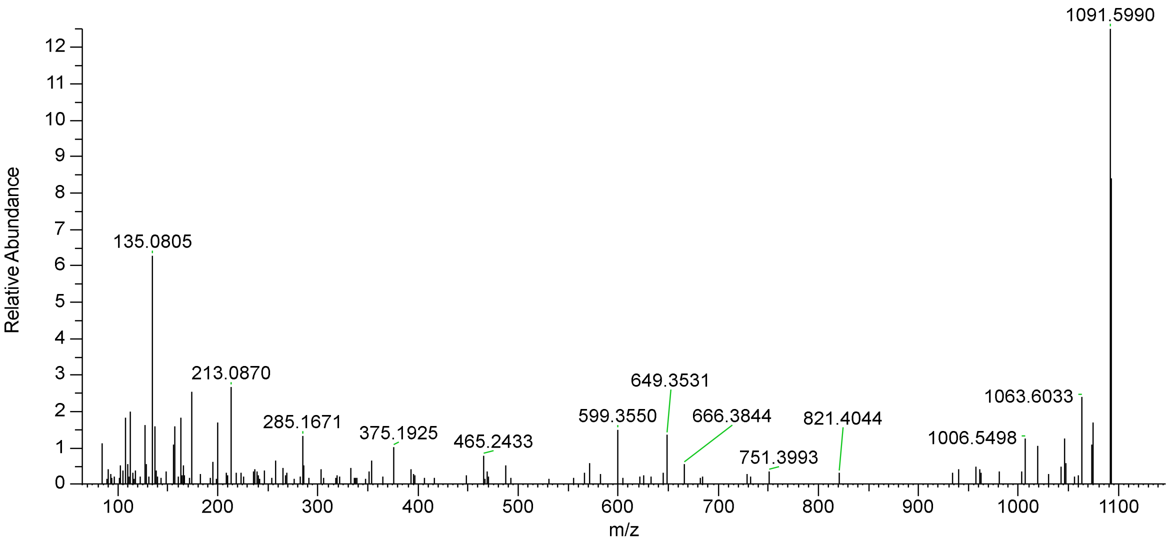

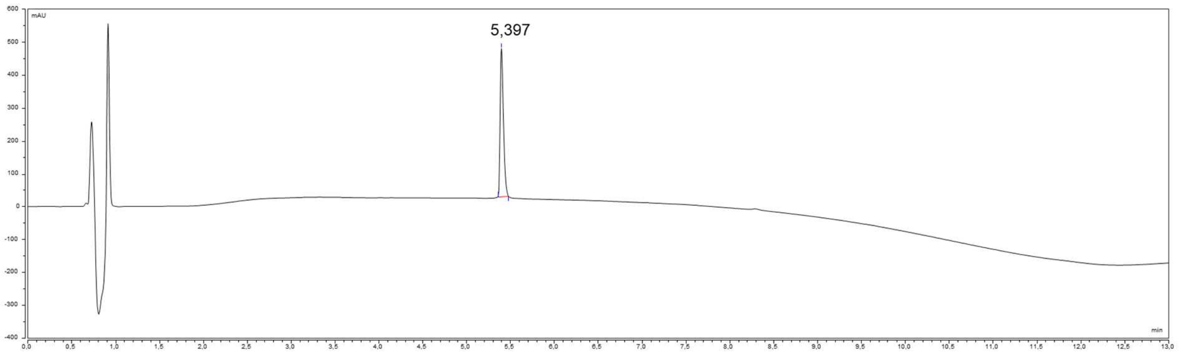

Figure S34. Structure, MS, and MS[2] data of **4y**. Key fragment b5/y5 (Mdha-Ala-AznlePram-Masp-Arg) *m/z* 649.3531 (0.0056 Da). HPLC-DAD chromatogram at 210 nm.

Microcystin-[Azidonorleucine | *N*6-[(2-propyn-1-yloxy)carbonyl]-L-lysine]R (4z)

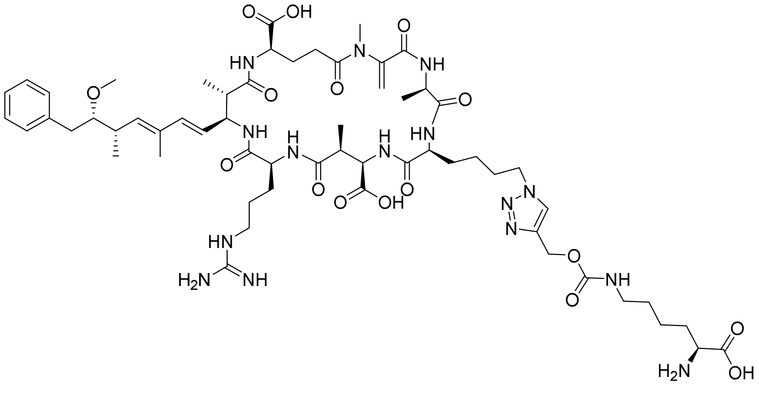

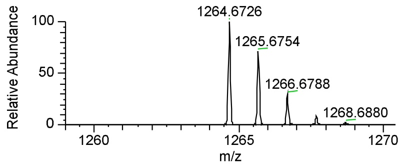

Sum formula

$C_{59}H_{89}N_{15}O_{16}$

pred. *m/z* ([M + H]$^+$)

1264.6684

meas. *m/z* ([M + H]$^+$)

1264.6726 (Δ 3.3 ppm)

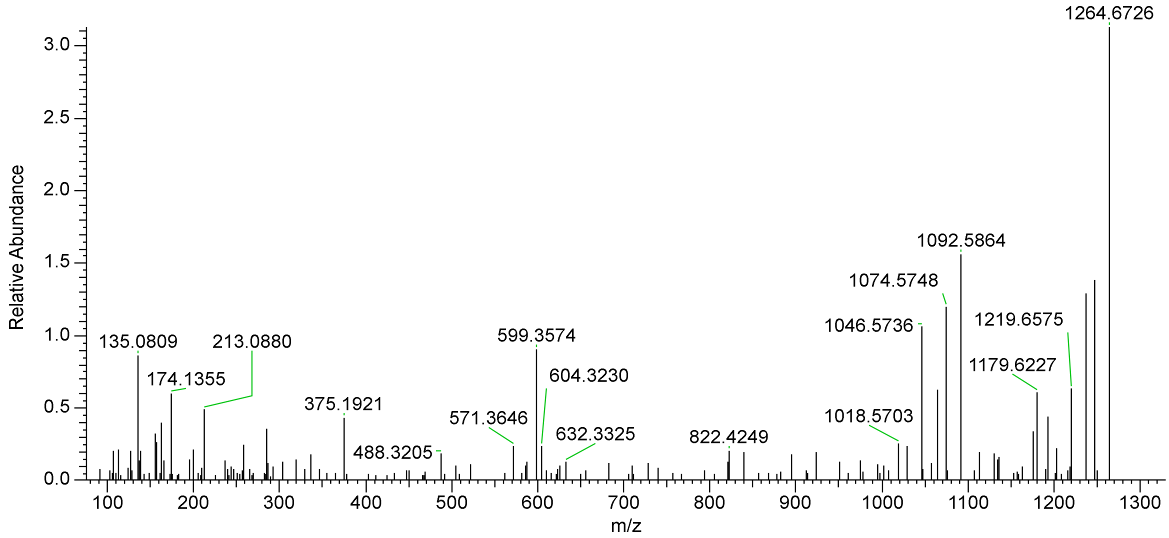

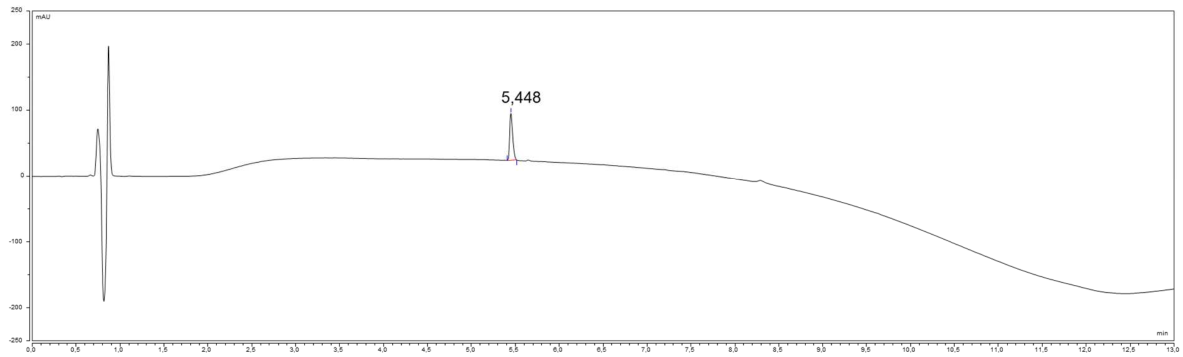

Figure S35. Structure, MS, and MS^2 data of **4z**. Key fragment b5/y5 (Mdha-Ala-AznlePrlys-Masp-Arg) *m/z* 822.4296 (0.0080 Da). HPLC-DAD chromatogram at 210 nm.

Microcystin-L[Azidonorleucine] (5)

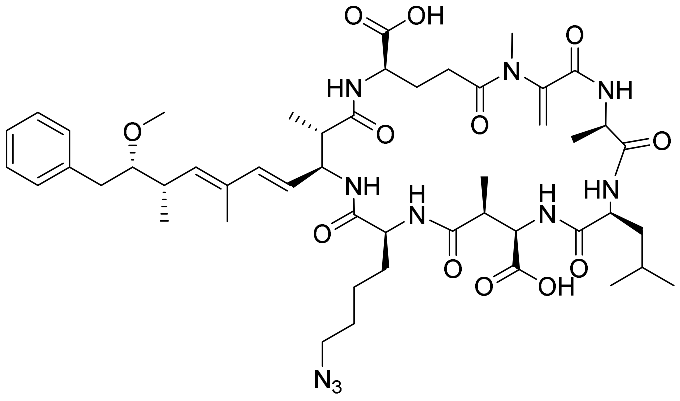

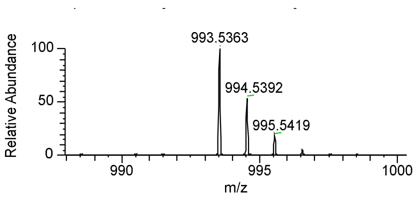

Sum formula

$C_{49}H_{72}N_{10}O_{12}$

pred. *m/z* ([M + H]$^+$)

993.5404

meas. *m/z* ([M + H]$^+$)

993.5363 (Δ 4.1 ppm)

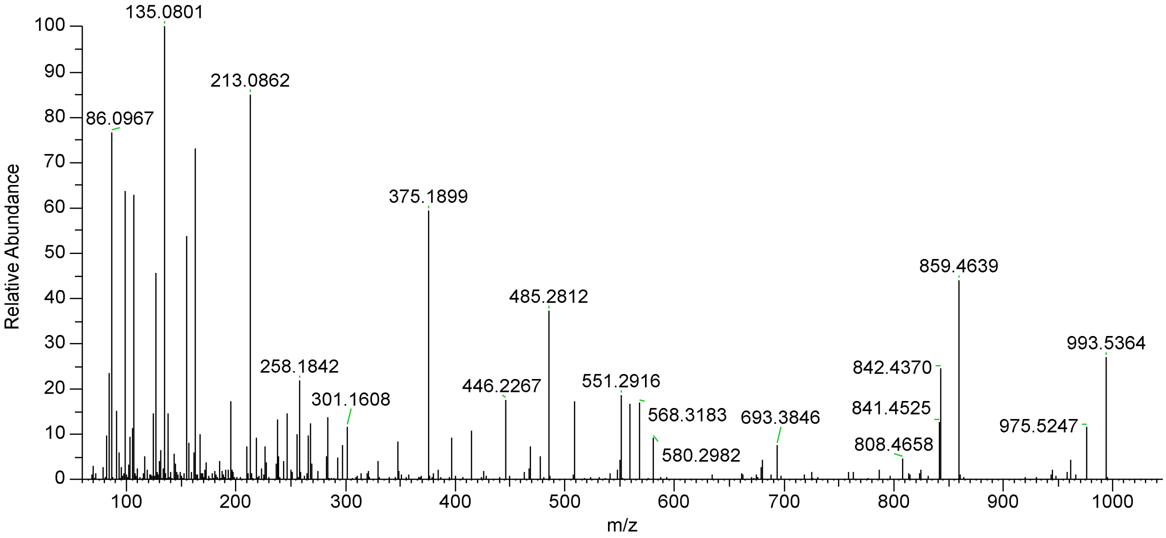

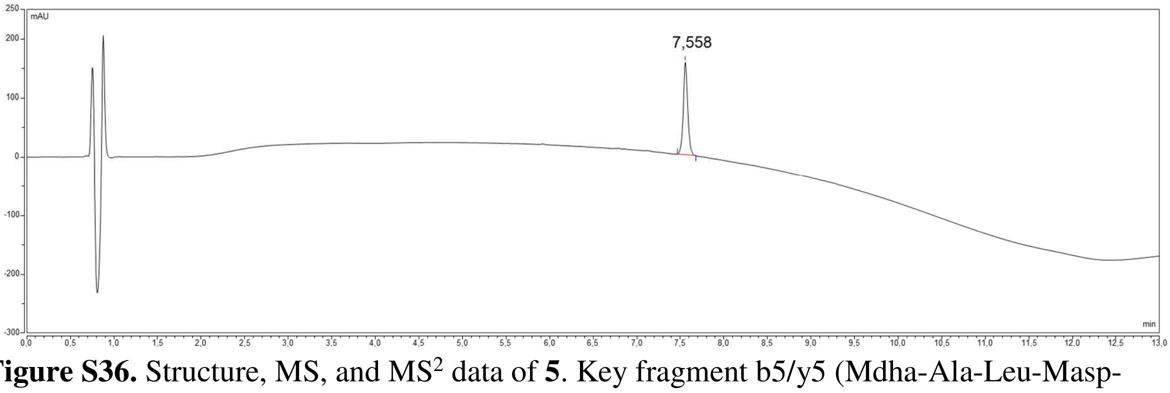

Figure S36. Structure, MS, and MS^2 data of **5**. Key fragment b5/y5 (Mdha-Ala-Leu-Masp-Aznle) *m/z* 551.2916 (0.0020 Da). HPLC-DAD chromatogram at 210 nm.

Table S7. Detailed evaluation of the MS/MS spectrum of **5**. Fragments indicated in bold are only explainable if the clickable amino acid is incorporated in the postulated position of the MC core structure.

m/z	**annotation**	**ion**	**difference [Da]**
993.5364	Ala-Leu-Masp-Aznle-Adda-Glu-Mdha + H^+	-	0.0040
975.5247	Ala-Leu-Masp-Aznle-Adda-Glu-Mdha + H^+ - H_2O	-	0.0052
965.5416	Ala-Leu-Masp-Aznle-Adda-Glu-Mdha + H^+ - N_2	-	0.0073
726.0046	**Masp-Aznle-Adda-Glu + H^+**	b4/y4	0.0046
693.3846	Adda-Glu-Mdha-Ala-Leu + H^+	z5	0.0011
680.3370	**Glu-Mdha-Ala-Leu-Masp-Aznle + H^+**	b6/y6	0.0008
662.3198	**Glu-Mdha-Ala-Leu-Masp-Aznle + H^+ - H_2O**	b6/y6	0.0058
580.2982	Adda-Glu-Mdha-Ala + H^+	z4	0.0036
551.2916	Mdha-Ala-Leu-Masp-Aznle + H^+	b5/y5	0.0020
509.2627	Adda-Glu-Mdha + H^+	z3	0.0019
508.2373	**Glu-Mdha-Ala-Leu-Masp + H^+ - H_2O**	b5/y5	0.0029
468.2547	Ala-Leu-Masp-Aznle + H^+	b4/y4	0.0018
426.2260	Adda-Glu + H^+	z2	0.0008
397.2091	**Mdha-Ala-Leu-Masp + H^+** or<br>**Glu-Mdha-Ala-Leu + H^+**	b4/y4	0.0015
379.1951	**Glu-Mdha-Ala-Leu + H^+ - H_2O**	b4/y4	0.0025
314.2129	Adda + H^+	y1	0.0015
314.1728	**Ala-Leu-Masp + H^+**	b3/y3	0.0017
297.1836	Adda + H^+	z1	0.0013
284.1346	Masp-Aznle + H^+	b2/y2	0.0007
268.1645	**Mdha-Ala-Leu + H^+**	b3/y3	0.0011
266.1137	Glu-Mdha-Ala + H^+ - H_2O	b3/y3	0.0002
243.1342	Leu-Masp + H^+	b2/y2	0.0002
213.0862	Glu-Mdha + H^+	b2/y2	0.0008
196.0599	Glu-Mdha + H^+	z2	0.0006
195.0757	Glu-Mdha + H^+ - H_2O	b2/y2	0.0007
185.1281	**Ala-Leu + H^+**	b2/y2	0.0004
155.0810	Mdha-Ala + H^+	b2/y2	0.0005
138.0545	Mdha-Ala + H^+	z2	0.0005
130.0495	Glu + H^+ or<br>Masp + H^+	y1	0.0004
127.0863	Aznle + H^+ - N_2	y1	0.0003
114.0916	Leu + H^+	y1	0.0003

112.0388	Glu + H^+ - H_2O	y1	0.0005
110.0602	Aznle + H^+ - N_2	z1	0.0002
84.0447	Mdha + H^+	y1	0.0003

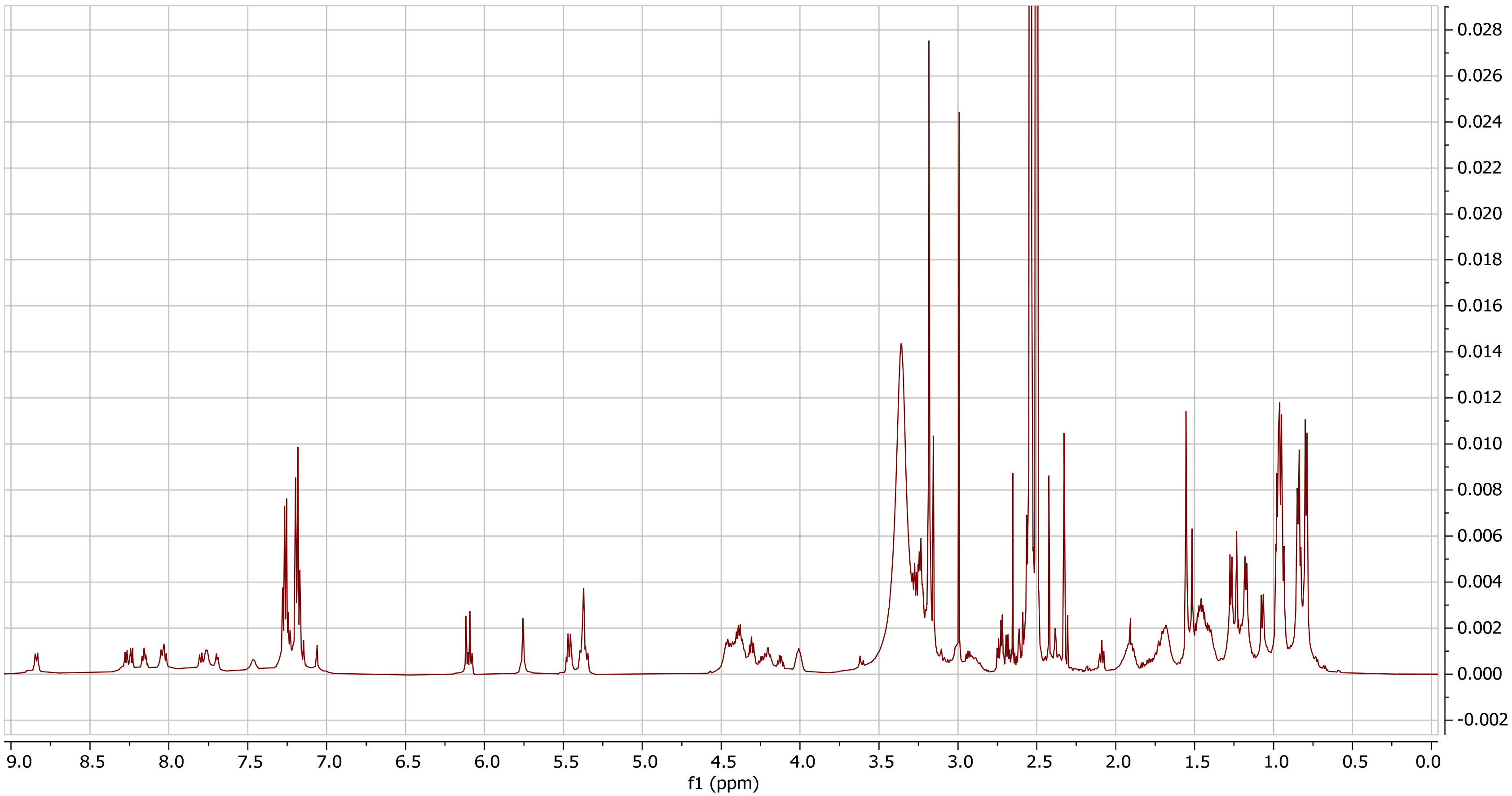

Figure S37. ^{1}H NMR spectrum of **5** at 600 MHz (DMSO).

Microcystin-L[Azidonorleucine | carboxamide-propargylbiotin] (5w)

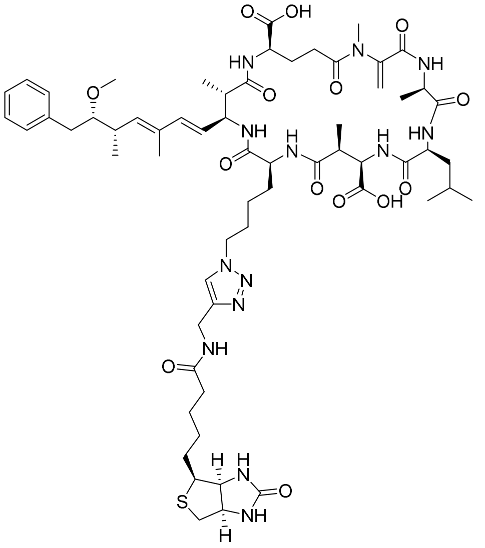

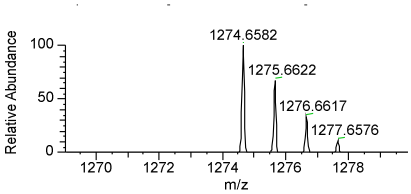

Sum formula
$C_{62}H_{91}N_{13}O_{14}S$
pred. *m/z* ([M + H]⁺)
1274.6602
meas. *m/z* ([M + H]⁺)
1274.6582 (Δ 1.6 ppm)

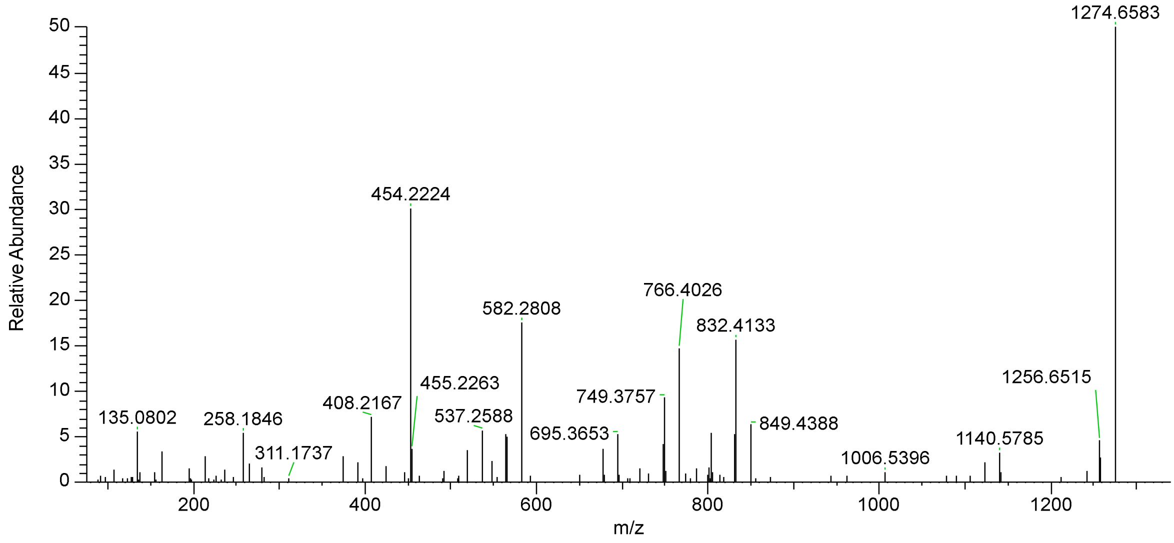

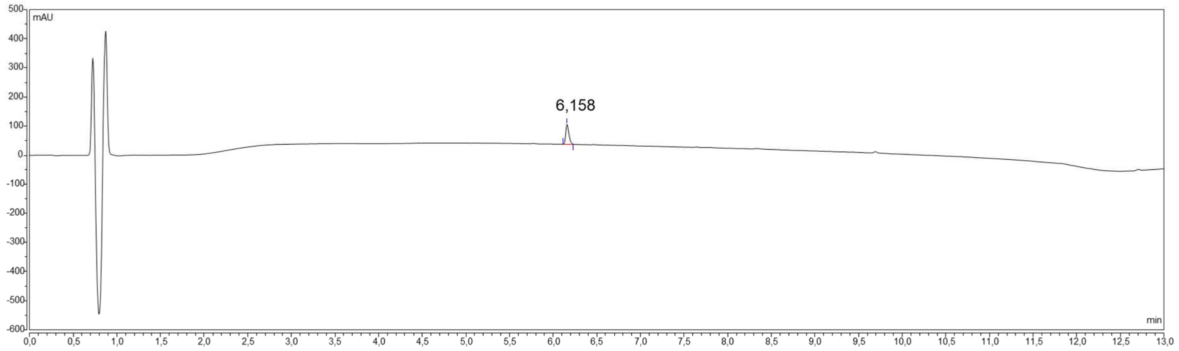

Figure S38. Structure, MS, and MS² data of **5w**. Key fragment b5/y5 (Mdha-Ala-Leu-Masp-AznlePrbio) *m/z* 832.4133 (0.0001 Da). HPLC-DAD chromatogram at 210 nm.

Microcystin-L[Azidonorleucine | chlorpentyne] (5x)

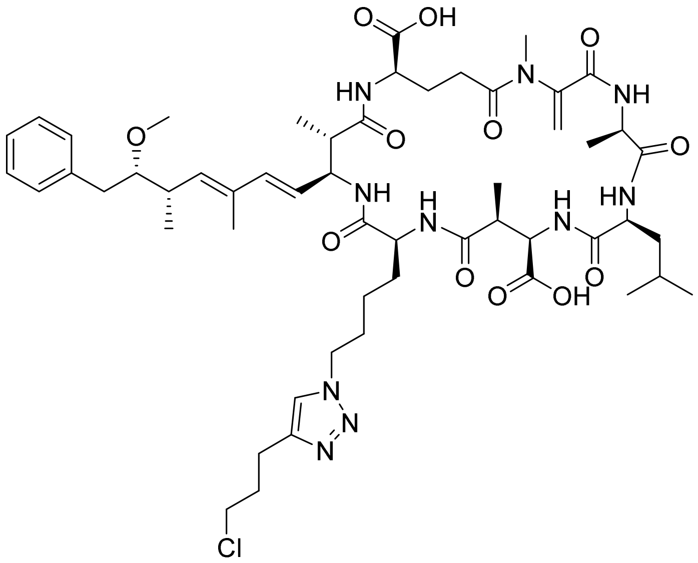

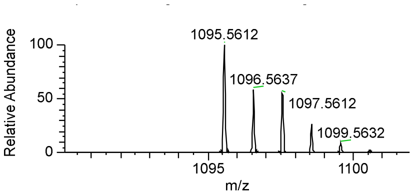

Sum formula

$C_{54}H_{79}ClN_{10}O_{12}$

pred. ***m/z*** **([M + H]⁺)**

1095.5640

meas. ***m/z*** **([M + H]⁺)**

1095.5612 (Δ 2.6 ppm)

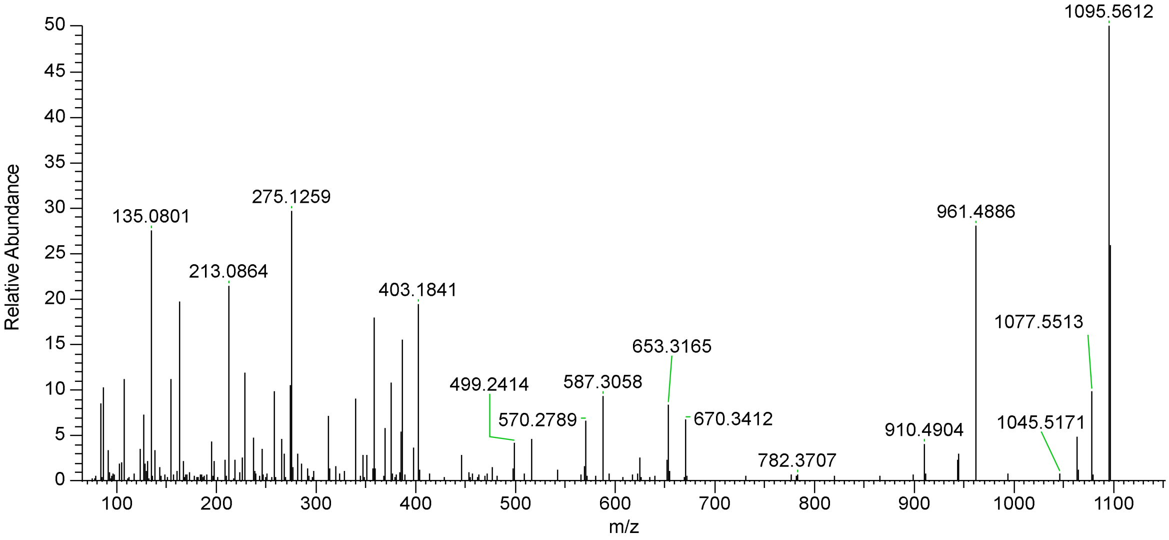

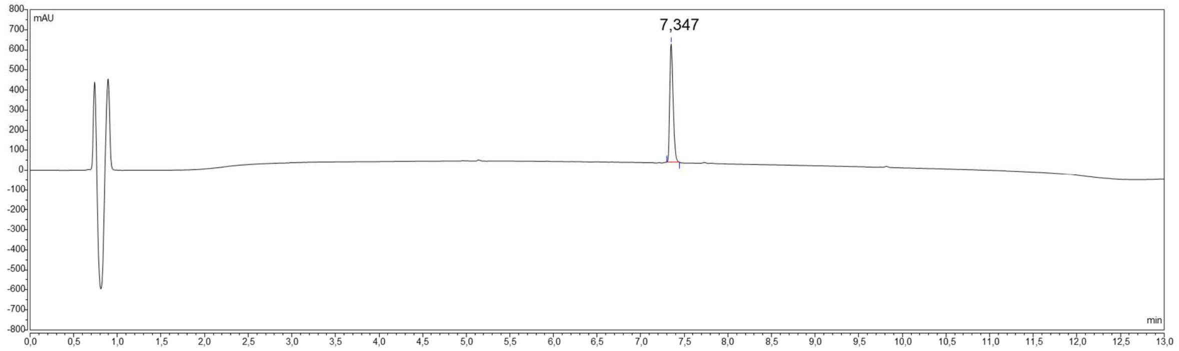

Figure S39. Structure, MS, and MS² data of **5x**. Key fragment b5/y5 (Mdha-Ala-Leu-Masp-AznleChlopyne) *m/z* 653.3165 (0.0008 Da). HPLC-DAD chromatogram at 210 nm.

Microcystin-L-[Azidonorleucine | propargylamine] (5y)

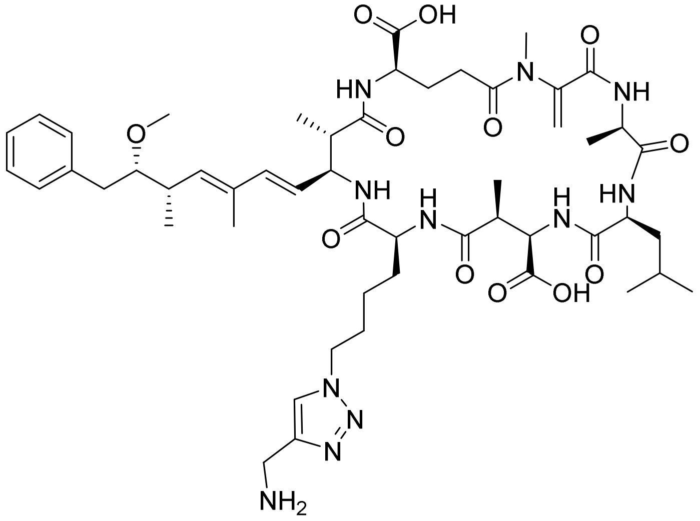

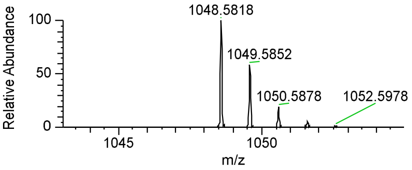

Sum formula

$C_{52}H_{77}N_{11}O_{12}$

pred. ***m/z*** **([M + H]$^+$)**

1048.5826

meas. ***m/z*** **([M + H]$^+$)**

1048.5818 (Δ 0.8 ppm)

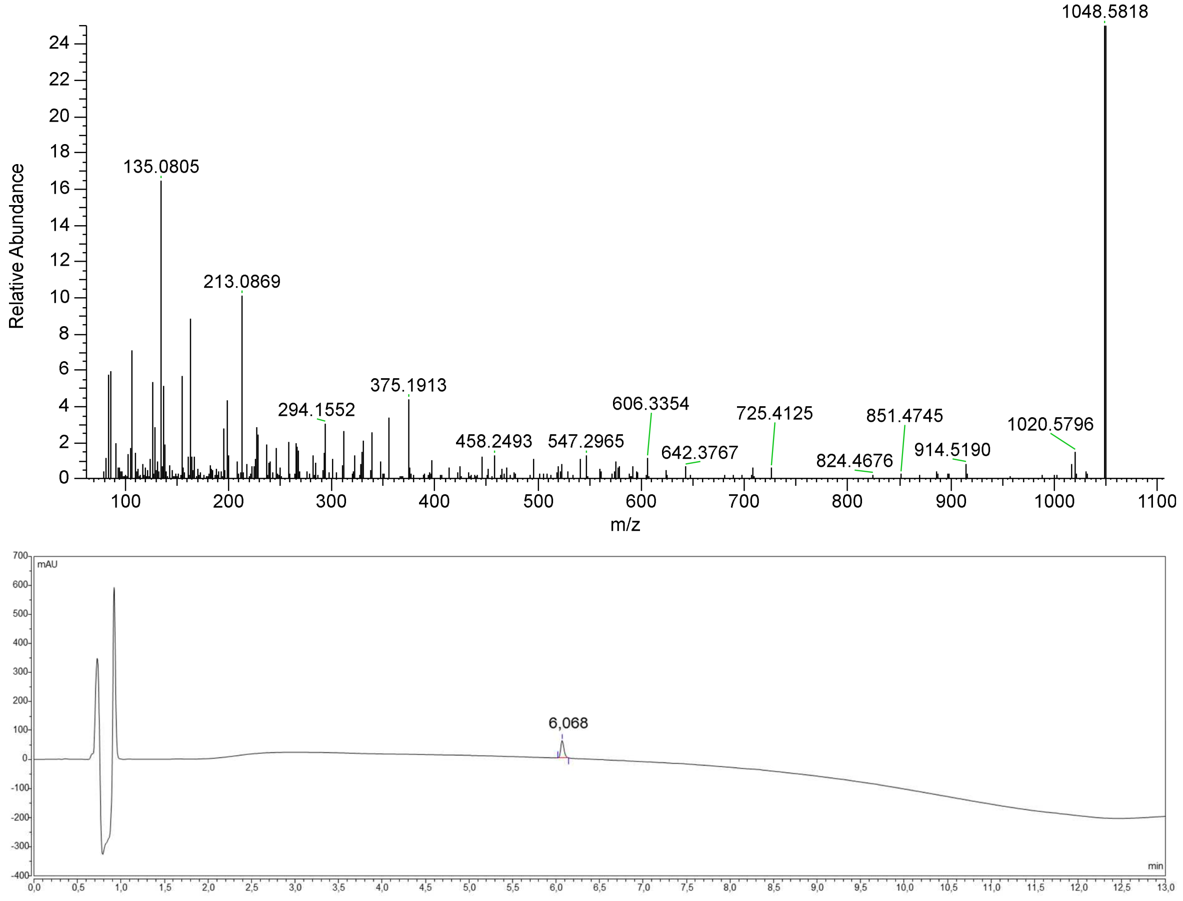

Figure S40. Structure, MS, and MS^2 data of **5y**. Key fragment b5/y5 (Mdha-Ala-Leu-Masp-AznlePram) *m/z* 606.3354 (0.0004 Da). HPLC-DAD chromatogram at 210 nm.

Microcystin-L[Azidonorleucine|*N*6-[(2-propyn-1-yloxy)carbonyl)]-L-lysine] (5z)

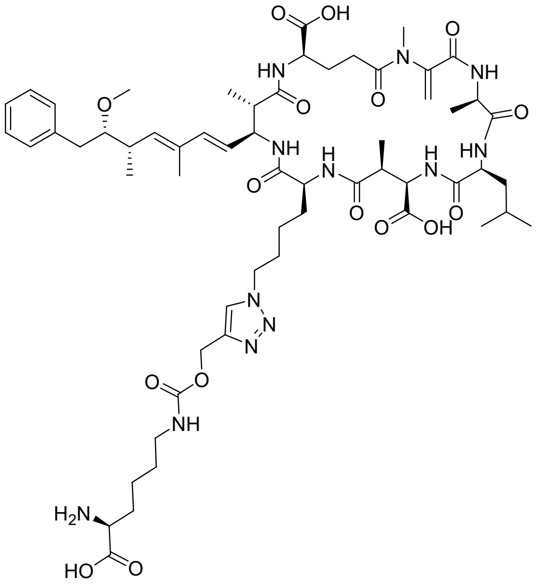

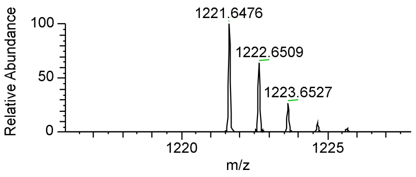

Sum formula

$C_{59}H_{88}N_{12}O_{16}$

pred. *m/z* ([M + H]⁺)

1221.6514

meas. *m/z* ([M + H]⁺)

1221.6476 (Δ 3.1 ppm)

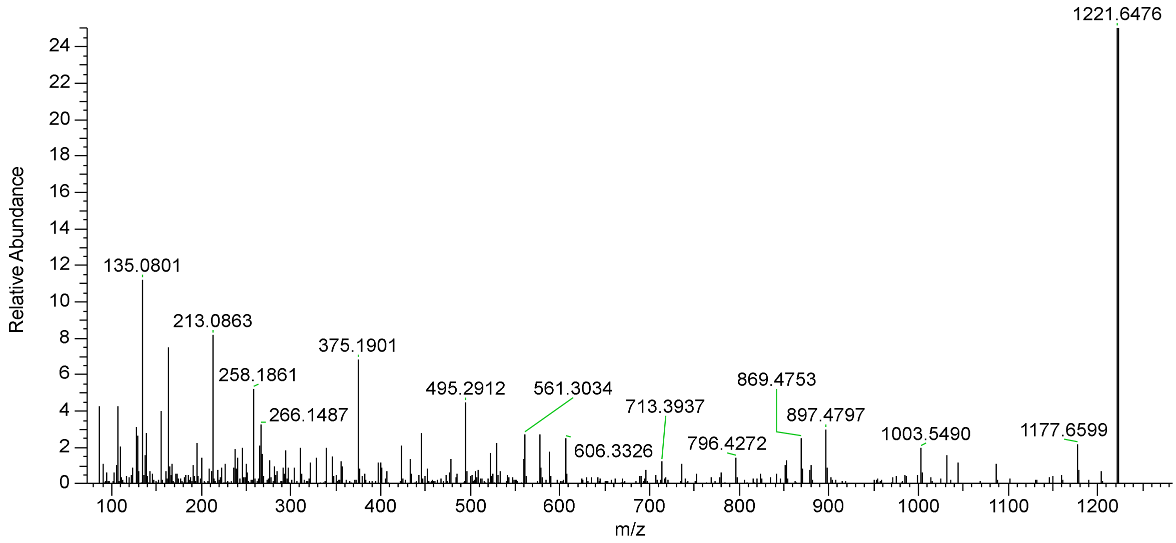

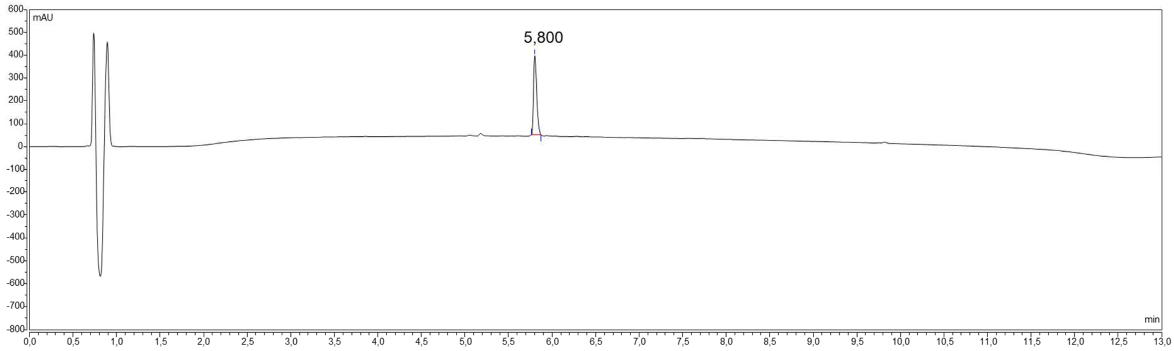

Figure S41. Structure, MS, and MS^2 data of **5z**. Key fragment b5/y5 (Mdha-Ala-Leu-Masp-AznlePrlys) *m/z* 696.3661 (0.0014 Da). HPLC-DAD chromatogram at 210 nm.
